# Mild Photochemical Reduction of Alkenes and Heterocycles via Thiol-Mediated Formate Activation

**DOI:** 10.1021/acs.orglett.4c01894

**Published:** 2024-06-25

**Authors:** Carter U. Brzezinski, Andrew R. LeBlanc, Madeline G. Clerici, William M. Wuest

**Affiliations:** Department of Chemistry, Emory University, Atlanta, Georgia 30322, United States

## Abstract

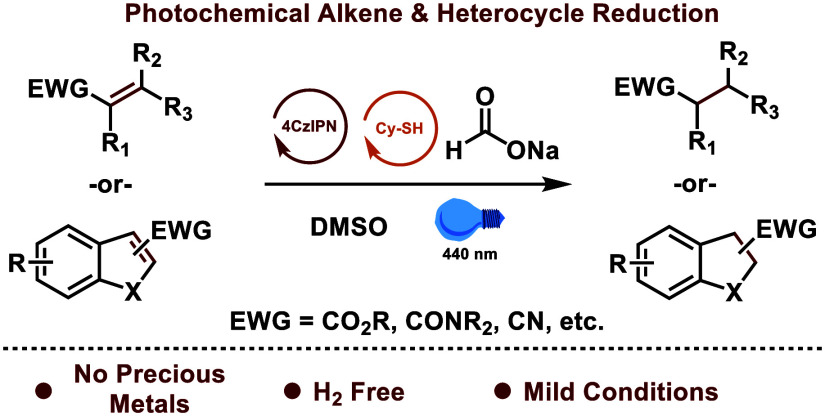

The reduction of alkenes to their respective alkanes is one of the most important transformations in organic chemistry, given the abundance of natural and commercial olefins. Metal-catalyzed hydrogenation is the most common way to reduce alkenes; however, the use of H_2_ gas in combination with the precious metals required for these conditions can be impractical, dangerous, and expensive. More complex substrates often require extremely high pressures of H_2_, further emphasizing the safety concerns associated with these hydrogenation reactions. Here we report a safe, cheap, and practical photochemical alkene reduction using a readily available organophotocatalyst, catalytic thiol, and formate. These conditions reduce a variety of di-, tri-, and tetra-substituted alkenes in good yield as well as dearomatize pharmaceutically relevant heterocycles to generate sp^3^-rich isosteres of benzofurans and indoles. These formal-hydrogenation conditions tolerate a broad range of functionalities that would otherwise be sensitive to typical hydrogenations and are likely to be important for industry applications.

Given the abundance of natural and commercial alkenes as well as the many types of olefination transformations used for C–C bond formation, alkene reduction is one of the most important reactions across the fields of complex molecule synthesis, polymer chemistry, and other chemical manufacturing processes.^1^ Hydrogenations using precious metals such as palladium, platinum, rhodium, etc. and gaseous H_2_ is the most common strategy used for these reductions (Scheme 1A); however, these systems can be impractical due to safety concerns stemming from the pyrophoric nature of these metal adducts and the flammable nature of H_2_ gas.^2^ Many pharmaceutically relevant substrates for metal-catalyzed hydrogenation require extremely high-pressures of H_2_ gas, further reducing the functional group tolerance of these reactions. Performing these high-pressure hydrogenations requires expensive, specialized reaction vessels that may be unavailable in certain academic settings and requires significantly higher safety precautions. Metal-catalyzed hydrogenation can also be costly and has a significant environmental impact, particularly for asymmetric hydrogenation, due to the frequent use of nonabundant metals such as rhodium, iridium, and ruthenium.^3^ For context, the environmental burden of palladium and rhodium exceeds 3,500 and 35,000 kg of CO_2_/kg, respectively.^4^ For these reasons, there is a need for new reactivity modes to perform alkene reductions in a safer, greener, and operationally simpler manner with an improved functional group tolerance. Metal-hydride atom transfer (MHAT) is a well-developed alternative for many types of alkene reductions and functionalizations and often favors the formation of the thermodynamic product rather than the kinetic one typically obtained by metal-catalyzed hydrogenation.^5,6^ Many electrochemical alkene^7,8^ and alkyne^9^ reductions have also been developed, as well as the electrochemical Birch-type reductions from the Baran group^10,11^ and others.^12^

**Scheme 1:**
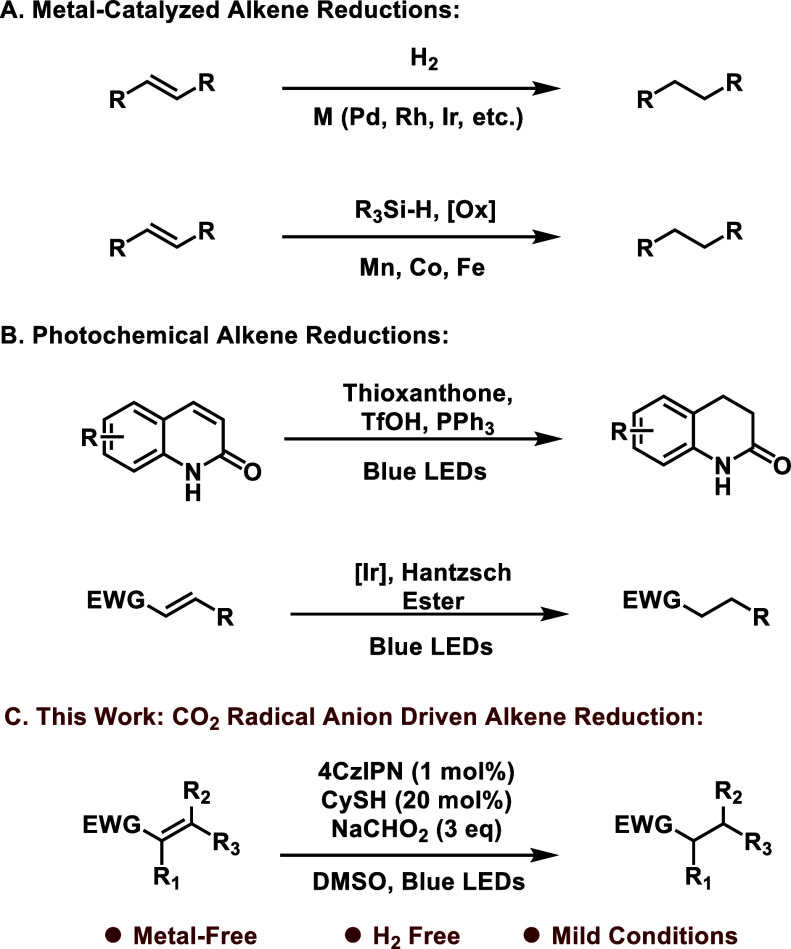
Catalytic Strategies for Alkene Reduction

Photochemical reduction of alkenes to their respective radical anions via single-electron transfer is well precedented, and frequently used for further transformations including hydrofunctionalization,^13^ cyanation,^14^ carboxylation,^15^ aminocarboxylation,^16^ cyclizations,^17^ [2 + 2]^18,19^ and [3 + 2]^20^ cycloadditions, and others.^21^ Photochemical hydrogenation via alkene and arene single-electron reductions has more recently emerged (Scheme 1B) and offers many advantages over metal-catalyzed hydrogenation. Kang and Guo et al. recently reported the reduction of a variety of quinolones and unsaturated esters using a thioxanthone photocatalyst, PPh_3_, and H_2_O as hydrogen-atom-transfer (HAT) agents.^22^ Larionova et al. also reported the reduction of a variety of cinnamate esters and other activated alkenes using an iridium photocatalyst and Hantzsch ester as hydrogen atom donors.^23^ While both conditions are excellent examples of safer reaction conditions for alkene reductions, the former suffers from the generation of P(O)Ph_3_ as a byproduct and the necessity of additive TfOH, and the latter requires a prohibitively expensive iridium photocatalyst and has a poor tolerance of heterocyclic functionality. Hendy et al. previously demonstrated a radical chain mechanism for the formation of the highly reducing CO_2_^•–^ (*E*_v_ = −2.2 V) from formate via HAT from a thiyl radical, which then reduces a variety of activated chlorides, trialkylammonium salts, and sulfonamides.^24^ Additionally, the Jui and Wickens^25−27^ group each demonstrated that alkenes and heteroarenes could intercept this radical to affect a carboxylation reaction, whereas a pyridyl-cinnamate was instead reduced. This cinnamate reduction was proposed to operate via single-electron-transfer (SET); however, the exact mechanism that terminates this process was not elucidated. Chiba et al. also recently demonstrated a variety of Birch-type reductions using a highly reducing polysulfide dianion (*E*_v_ = −2.5 V) and formate system.^28^ Herein we report a convenient, cost-effective, safe, and practical protocol for alkene and heterocycle reduction using the widely available organophotocatalyst 4CzIPN, catalytic thiol, and formate.

Related to our own lab’s interest in medicinal chemistry and target identification of novel small-molecule antibiotics, we found a need for the generation of a library of diaryl propanoic acid fragments (Table 1). Hydrogenation of the easily accessible unsaturated ester with either atmospheric hydrogen or metal-catalyzed conjugate addition strategies was unsuccessful with heterocyclic fragments. We wondered if a photochemical reduction of this alkene via SET from formate might be a more effective strategy and could lead to the optimization of simple, hydrogen-free reduction of these highly conjugated substrates. Informed by our group’s previous expertise using formate in photoredox catalysis,^29^ we screened a library of additives and found that the combination of the inexpensive organophotocatalyst 4CzIPN, catalytic cyclohexane thiol (Cy-SH), and 3 equiv of sodium formate in DMSO under 440 nm LED irradiation affected clean conversion of **1** to the reduced product (entry 1). Alternative additives such as thiophenol and PhN(Tf)_2_ were also effective for this reduction; however, we chose to continue using CySH due to its low cost and more consistent conversion of **1**. Further optimization studies of these conditions showed that 1 mol % of 4CzIPN was necessary for full conversion, and that solvent concentrations as high as 1.0 M were tolerated. We generally found that sparging the DMSO solvent with argon yielded the most consistent conversions of **1**, and that the inclusion of solvent quantities of water had minimal effect on the performance of this reduction. Control experiments without 4CzIPN, NaCHO_2_, or 440 nm LEDs exclusively returned SM. This reduction is operable in the absence of CySH, though this is likely due to background decomposition of DMSO and is consistent with our group’s previous work.^29^

**Table 1:**
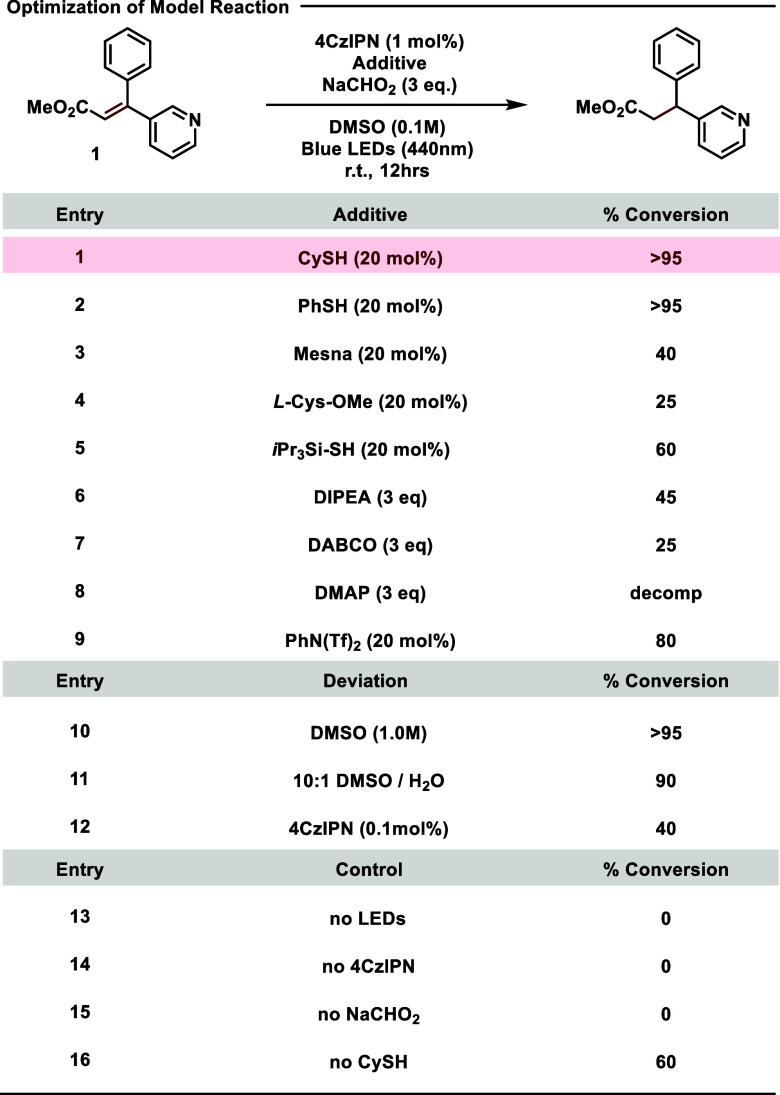
Optimization of Alkene Reduction^a^

a All screening reactions were performed on a 0.10 mmol scale. Conversion determined via ^1^H NMR.

With these optimized conditions in hand, we first investigated the scope of the alkene reductions (Table 2). Benzyl cinnamate ester **2** was readily reduced under these conditions, highlighting the compatibility of benzyl-protecting groups that would otherwise be labile under hydrogenation conditions. Reduced amide **3** and ester **4** demonstrate the potential for alternative electron withdrawing groups and alpha-substitution, as does tetrasubstituted cyano-acrylate **5**. Carbamates **6** and **7** were also cleanly reduced without the removal of the H_2_-labile benzyl carbamate. Trisubstituted alkenes **7**–**13** demonstrate the compatibility of these reactions with a variety of functionality, including electron donating, electron withdrawing, heterocyclic, and fluorinated containing moieties. The presence of an aromatic substitution is not strictly necessary as demonstrated by ketal **15**, and **16** was obtained via reduction of the Wieland–Miescher-ketone-derived ketal with a slight preference for the thermodynamically favored trans-decalin.

**Table 2:**
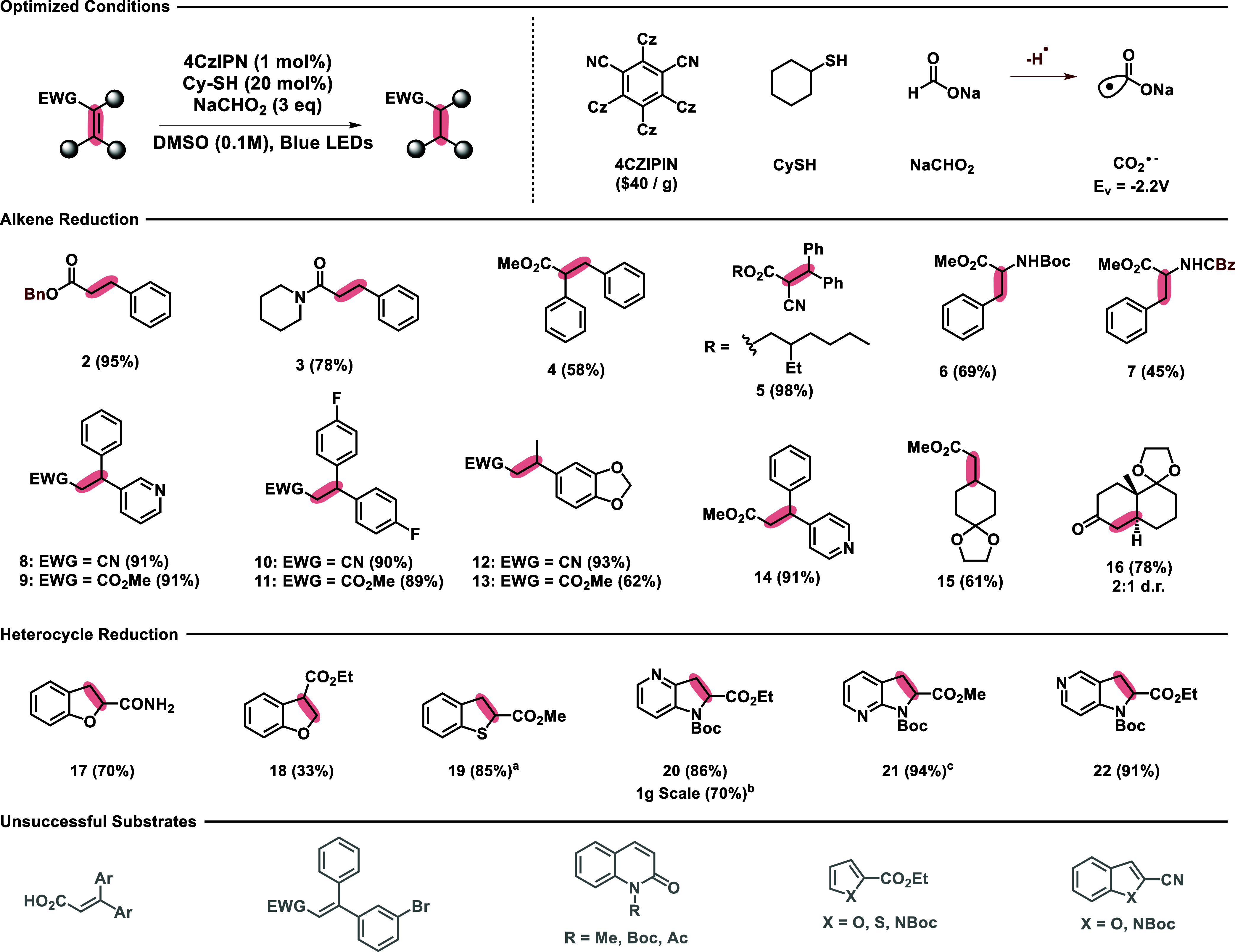
Scope of Photochemical Alkene Reduction^a^

a All yields unless stated otherwise are isolated. (a) NMR yield calculated using dibromomethane as an internal standard. PhSH was used instead of CySH. (b) Performed with 0.25 M DMSO. (c) Performed on 0.16 mmol scale.

Given our success of reducing highly conjugated tri- and tetra-substituted alkenes, we wondered if these conditions could be applied toward the reduction of heterocyclic substrates. We imagined that such reductions could be particularly advantageous for the synthesis of sp^3^-rich isosteres of benzofuran and indole building blocks, as these electron rich heterocycles can be problematic in medicinal chemistry settings due to their metabolic liabilities.^30^ Excitingly, benzofurans **17** and **18** were reduced to their respective dihydrobenzofurans in moderate yield. Benzothiophene **19** was also efficiently reduced, though interestingly we observed cleaner reaction profiles using PhSH instead of CySH. Thiophene containing substrates can be especially problematic for metal-catalyzed hydrogenation due to catalyst poisoning, demonstrating the advantages of these conditions. Aza-indoles **20**–**22** were also successfully reduced to their respective aza-indolines in high yield and performed well on a 1 g scale. We note that furans and pyrroles tended to decompose via polymerization under these reaction conditions and that many carboxylic acids and aryl-bromides were not tolerated.

To further demonstrate the potential applications and functional-group tolerance of these conditions, we prepared two representative amide-coupled substrates to yield the reduced amido-quinoline **23** and dihydrobenzofuran **24** (Figure 1A). We also highlight the advantages of this photochemical reduction over reported patent and literature examples of comparable heterogeneous hydrogenations and show that these conditions can perform reductions inaccessible by hydrogenation (Figure 1B).^31−33^ In each case, our conditions yield the desired products (**25**–**27**) in good yield without the need for high pressures of H_2_. Product **28** emphasizes the advantageous selectivity these conditions offer, as heterogeneous hydrogenation with H_2_ and Pd/C typically dehalogenates aryl systems.

**Figure 1:**
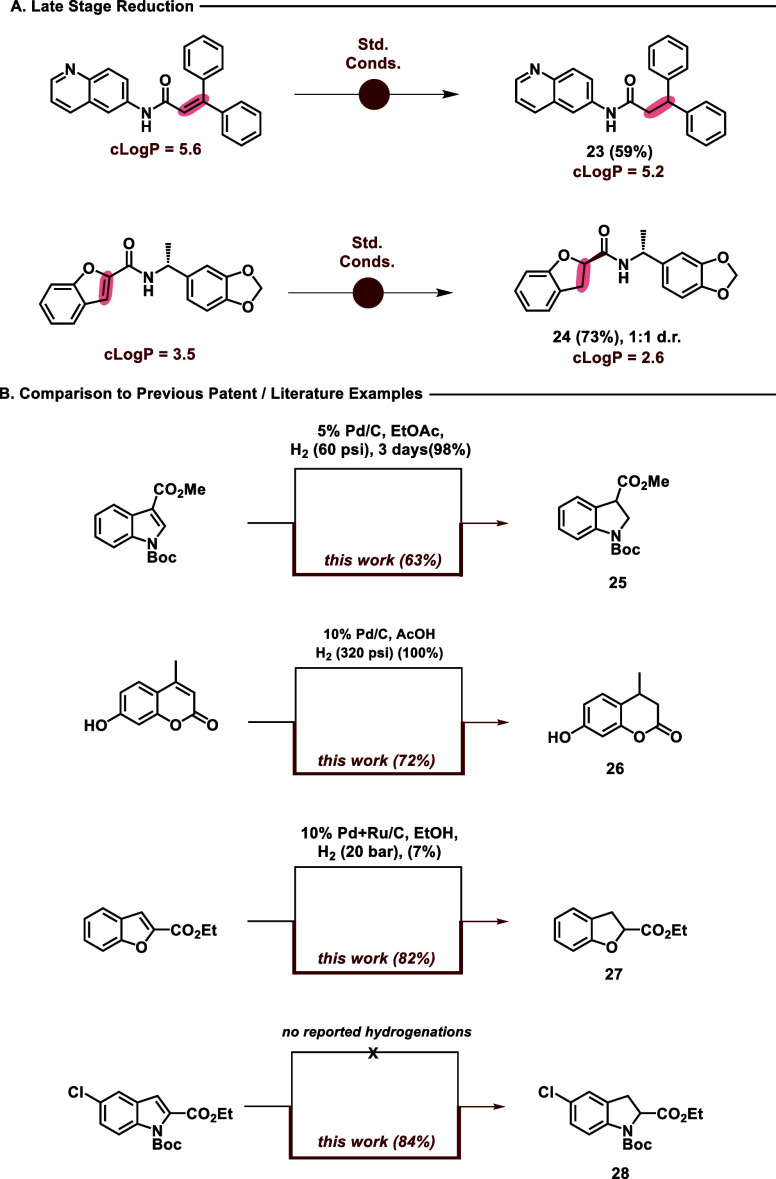
(A) Late-stage reduction of amide-coupled intermediates. (B) Comparison to patents (**25**, Gruenenthal and **26**, Unilever) and literature reported heterogeneous hydrogenation conditions.

Based on precedent from the Jui and Wickens group, we propose that this alkene reduction operates via SET from CO_2_^•–^ through a radical anion intermediate. Generation of this reducing radical anion likely occurs via 4CzIPN oxidation of Cy-SH to form a thiyl radical (Scheme 2), which can abstract hydrogen from sodium formate. Further mechanistic experiments to understand the nature of these radical-anion intermediates and implement them for other transformations are underway.

**Scheme 2:**
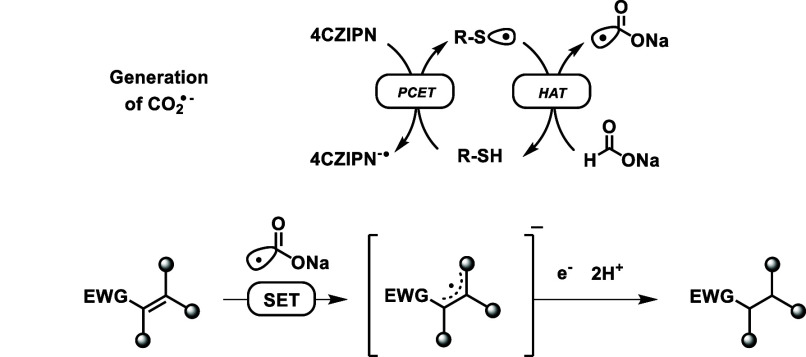
Proposed Mechanism of Alkene Reduction

In summary, we have developed a practical protocol for the reduction of di-, tri-, and tetra-substituted alkenes as well as the dearomatization of indoles and benzofurans. We demonstrate that these conditions outperform many typical heterogeneous hydrogenation conditions with respect to safety, functional group tolerance, and selectivity and can be applied to a variety of complex substrates. We expect that this transformation will find broad utility in medicinal and process chemistry and in the total synthesis community toward the practical synthesis of pharmaceuticals, agrochemicals, and other complex molecules of interest.

## Data Availability

The data underlying this study are available in the published article and its Supporting Information.

## Supporting Information Available

General procedures and spectra are provided. (PDF)

## Dedication

Dedicated to Prof. Amos B. Smith, III, founding Editor-in-Chief of *Organic Letters*, on the occasion of his 80th birthday.
