# Supplementary material for: Mild Photochemical Reduction of Alkenes and Heterocycles via Thiol-Mediated Formate Activation
Source: Org Lett. 2024 Jun 25;26(26):5534–8. doi: 10.1021/acs.orglett.4c01894 (PMC11232005; doi:10.1021/acs.orglett.4c01894)
Supplement: Supplementary file 1 — ol4c01894_si_001.pdf [file ol4c01894_si_001.pdf]

Mild Photochemical Reduction of Alkenes and Heterocycles via Thiol-mediated Formate Activation

Carter U. Brzezinski[‡], Andrew R. LeBlanc[‡], Madeline G. Clerici, William M. Wuest*

Department of Chemistry, Emory University, Atlanta, GA 30322

Email: william.wuest@emory.edu

Table of Contents

General Information 2
Abbreviations 2
General Photoredox Reaction Set Up 2
General Procedure for Optimization of Alkene Reductions 3
General Procedure for Substrate Scope 3
Emission Spectrum 4
Preparation of Products 4
Preparation of Starting Material/Reagents 19
Spectra 33
Citation 78

1) General Information

Nuclear magnetic resonance (^{1}H, ^{13}C NMR) spectra were recorded using the following spectrometers: Varian INOVA400, Varian INOVA500, Varian INOVA600, VNMR400, Bruker 400 (400 MHz or Bruker Avance III HD 600 equipped with cryo-probe. Chemical shifts are reported in parts per million (ppm) relative to the reference residual solvent peak. The following abbreviations are used to describe splitting: br (broad), s (singlet), d (doublet), t (triplet), q (quartet), p (pentet), m (multiplet), dd (doublet of doublets), dt (doublet of triplets), etc. Reactions were monitored by thin layer chromatography (TLC) using EMD Millipore Silica Gel 60 F_{254} precoated plates and visualized using UV and/or $KMnO_4$, Vanillin or 2,4-dinitrophenyl-hydrazine (DNP) stains. Brine refers to a saturated solution of sodium chloride. Column chromatography was performed using a Biotage® flash chromatography purification system or via a traditional hand column. Both methods used Siliaflash silica gel (40-63 μm). Chemicals were used as received from Oakwood, Sigma-Aldrich, Alfa Aesar, or AK Scientific unless stated otherwise. All reactions were carried out on the bench top. Photochemical reactions were subjected to blue LEDs (LEDwholesalers PAR38 Indoor Outdoor 16-Watt LED Flood Light Bulb, Blue; or Hydrofarm® PPB1002 PowerPAR LED Bulb-Blue 15W/E27 (available from Amazon).

2) Abbreviations

DMSO = Dimethyl sulfoxide
4-CzIPN =1,2,3,5-Tetrakis(carbazol-9-yl)-4,6-dicyanobenzene
EtOAc = Ethyl acetate
THF = Tetrahydrofuran
DBU= 1,8-Diazabicyclo[5.4.0]undec-7-ene
DMAP= 4-Dimethylaminopyr
EDC = 1-Ethyl-3-(3-dimethylaminopropyl)carbodiimide

3) General Photoredox Reaction Set Up

A 3D-printed carousel was used to run multiple (up to 12) reactions at a time. Reactions were run in a 1–3-dram vials. A 15 W blue LED (described above) was place ~6 inches away from the vials.

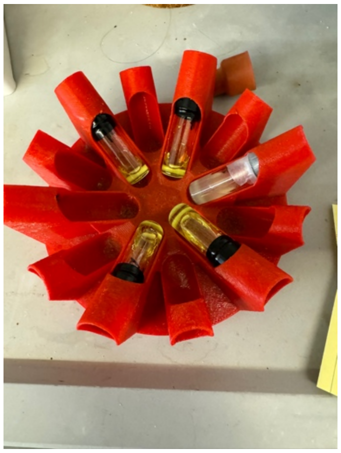

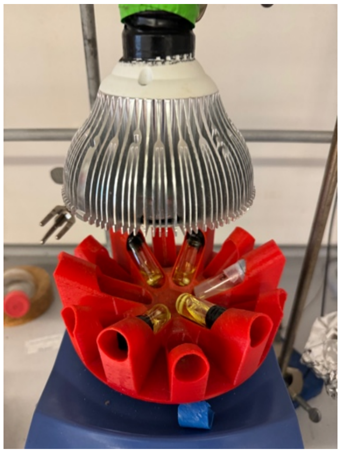

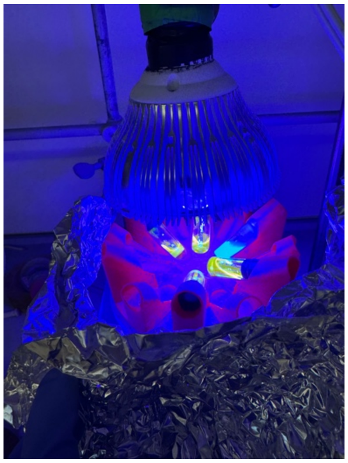

4) General Procedure for Optimization of Alkene Reduction

A 1-dram vial was charged with 2,4,5,6-tetra(9H-carbazol-9-yl)isophthalonitrile (789 µg, .01 Eq, 1.00 µmol) , sodium formate (20.4 mg, 3 Eq, 300 µmol), methyl (E)-3-phenyl-3-(pyridin-3-yl)acrylate (23.9 mg, 1 Eq, 0.100 mmol), and **additive, if solid (20 mol%, .02 mmol).** The vial was backfilled 3x with argon on a schlenck line, then DMSO (1.00 mL), degassed via Ar sparge for at least 15 minutes prior, was added followed by **additive, if liquid (20 mol%, .02 mmol).** The vial was capped, sealed with parafilm, placed under 440nm LEDs cooled by a fan, and stirred for 12 hours. The reaction was then diluted with EtOAc and water, the organic layer was separated, concentrated in vacuo, and analyzed by 1H-NMR for conversion.

5) General Procedure for Substrate Scope

A 1-dram vial was charged with **substrate, if solid (0.200 mmol, 1 equiv)**, sodium formate (40.8 mg, 3 Eq, 600 µmol), and 2,4,5,6-tetra(9H-carbazol-9-yl)isophthalonitrile (1.58 mg, .01 Eq, 2.00 µmol. The vial was backfilled 3x with argon on a schlenck line, then DMSO (2.00 mL), degassed via Ar sparge for at least 15 minutes prior, was added followed by cyclohexanethiol (4.65 mg, 4.9 µL, 0.2 Eq, 40.0 µmol) and **substrate, if liquid (0.200 mmol, 1 equiv)**. The vial was capped, sealed with parafilm, placed under 440nm LEDs cooled by a fan, and stirred for 12 hours. The reaction was then diluted with EtOAc and water, the organic layer was separated and washed with brine (3x 10ml), dried on sodium sulfates, and concentrated in vacuo. The crude reaction material was purified via normal-phase silica gel column chromatography.

Unless otherwise noted (see specific product entries), all substrates were tested at 0.200 mmol scale.

6) Emission Spectrum

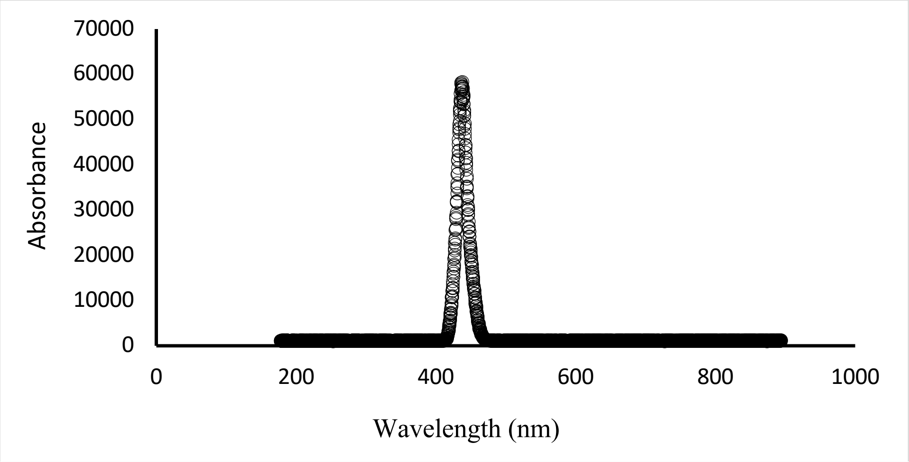

λ max = 440nm

7) Preparation of Products

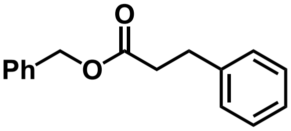

Benzyl 3-phenylpropanoate (2): Prepared according to general procedure (SI, Section 5). Benzyl cinnamate (50.0 mg, 0.21 mmol), 4CzIPN (3.3 mg, 2 mol%), sodium formate (42.8 mg, 3 equiv.), cyclohexanethiol (20 mol%, 0.042 mmol, 5.1 µL), DMSO (2mL, 0.1 M). The crude reaction was purified by silica chromatography (0--50% EtOAc/Hexanes gradient). 48.0 mg of the title compound was isolated (95% yield) as a clear oil. The physical and spectral data are consistent with reported values.[1]

^{1}H NMR (400 MHz, $CDCl_3$) δ 7.41 – 7.23 (m, 10H), 5.14 (s, 2H), 3.00 (t, *J* = 7.8 Hz, 2H), 2.72 (t, *J* = 7.8 Hz, 2H).

^{13}C NMR (101 MHz, $CDCl_3$) δ 172.8, 140.5, 136.0, 128.6, 128.6, 128.4, 128.3, 126.4, 66.4, 36.0, 31.0.

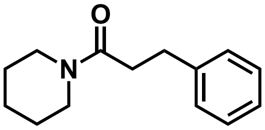

3-phenyl-1-(piperidin-1-yl)propan-1-one (3): Prepared according to general procedure (SI, Section 5). (E)-3-phenyl-1-(piperidin-1-yl)prop-2-en-1-one (43 mg, 0.20 mmol), 4CzIPN (3.2 mg, 2 mol%), sodium formate (41 mg, 3 equiv.), cyclohexanethiol (20 mol%, 0.040 mmol, 4.9 µL), DMSO (2mL, 0.1 M). The crude reaction was purified by silica chromatography (0—100% EtOAc/Hexanes gradient) to give 34 mg of an off colored solid (78% yield). The physical and spectral data are consistent with reported values.[2]

^{1}H NMR (400 MHz, $CDCl_3$) δ 7.36 – 7.18 (m, 5H), 3.62 – 3.54 (m, 2H), 3.39 – 3.32 (m, 2H), 3.03 – 2.96 (m, 2H), 2.69 – 2.60 (m, 2H), 1.64 (td, *J* = 6.8, 3.8 Hz, 2H), 1.57 – 1.52 (m, 2H), 1.52 – 1.43 (m, 2H).

^{13}C NMR (101 MHz, $CDCl_3$) δ 170.5, 141.6, 128.5, 128.5, 126.1, 46.7, 42.8, 35.3, 31.7, 26.5, 25.6, 24.6.

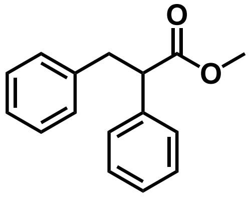

methyl 2,3-diphenylpropanoate (4):

Prepared according to general procedure (SI, Section 5). methyl (E)-2,3-diphenylacrylate (48 mg, 0.20 mmol), 4CzIPN (1.58 mg, 1 mol%), sodium formate (41 mg, 3 equiv.), cyclohexanethiol (20 mol%, 0.040 mmol, 4.9 µL), DMSO (2mL, 0.1 M). The crude reaction was purified by silica chromatography (0—30% Et_2O/Hexanes gradient) to give 28 mg (58% yield). The physical and spectral data are consistent with reported values.[3]
^{1}H NMR (400 MHz, $CDCl_3$) δ 7.37 – 7.10 (m, 10H), 3.89 (dd, *J* = 8.9, 6.7 Hz, 1H), 3.63 (s, 3H), 3.45 (dd, *J* = 13.7, 8.8 Hz, 1H), 3.06 (dd, *J* = 13.7, 6.7 Hz, 1H).
^{13}C NMR (101 MHz, $CDCl_3$) δ 173.9, 139.1, 138.7, 129.0, 128.7, 128.4, 128.0, 127.5, 126.5, 53.7, 52.1, 39.9.

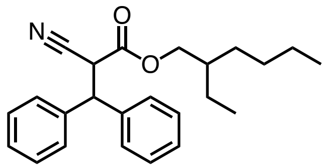

2-ethylhexyl 2-cyano-3,3-diphenylpropanoate (5): Prepared according to general procedure (SI, Section 5). Octocrylene (70 mg, 0.19 mmol, 67 µL), 4CzIPN (3.1 mg, 2 mol%), sodium formate (40 mg, 3 equiv.), cyclohexanethiol (20 mol%, 0.039 mmol, 4.97µL), DMSO (2mL, 0.1 M). The crude reaction was purified by silica chromatography (0—100% EtOAc/Hexanes gradient) to give 69 mg of an oil (98% yield). The physical and spectral data are consistent with reported values.[4]
^{1}H NMR (400 MHz, $CDCl_3$) δ 7.48 – 7.23 (m, 10H), 4.77 (d, *J* = 8.5 Hz, 1H), 4.29 (d, *J* = 8.5 Hz, 1H), 4.07 – 3.91 (m, 2H), 1.53 – 1.41 (m, 1H), 1.37 – 1.15 (m, 8H), 0.95 – 0.90 (t, 3H), 0.83 (t, *J* = 7.5 Hz, 3H).
^{13}C NMR (101 MHz, $CDCl_3$) δ 165.3, 139.5, 138.9, 129.0, 128.3, 127.9, 127.8, 127.7, 115.8, 69.2, 51.1, 43.7, 38.6, 30.1, 28.8, 23.5, 22.9, 14.1, 10.9.

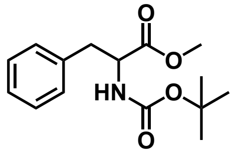

Methyl (*tert*-butoxycarbonyl)phenylalaninate (6): Prepared according to general procedure (SI, Section 5). Methyl-2-((tert-butoxycarbonyl)amino)-3-phenylacrylate (55.5 mg, 0.20 mmol), 4CzIPN (1.58 mg, 1 mol%), sodium formate (41 mg, 3 equiv.), cyclohexanethiol (20 mol%, 0.040 mmol, 4.9 µL), DMSO (2mL, 0.1 M). The crude reaction was purified by silica chromatography (0—100% EtOAc/Hexanes gradient) to give 38.2 mg (69% yield). The physical and spectral data are consistent with reported values.[5]
^{1}H NMR (400 MHz, $CDCl_3$) δ 7.35-7.23 (m, 3H), 7.18 – 7.12 (m, 2H), 4.99 (d, *J* = 8.3 Hz, 1H), 4.61 (q, *J* = 6.7 Hz, 1H), 3.74 (s, 3H), 3.11 (qd, *J* = 13.8, 6.0 Hz, 2H), 1.44 (s, 9H).
^{13}C NMR (101 MHz, $CDCl_3$) δ 172.5, 155.2, 136.1, 129.4, 128.6, 127.1, 80.0, 54.5, 52.3, 38.5, 28.4.

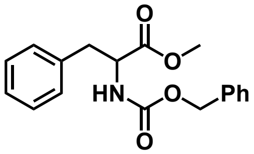

methyl ((benzyloxy)carbonyl)phenylalaninate (7): Prepared according to general procedure (SI, Section 5). Methyl-2-(((benzyloxy)carbonyl)amino)-3-phenylacrylate (62 mg, 0.20 mmol), 4CzIPN (1.58 mg, 1 mol%), sodium formate (41 mg, 3 equiv.), cyclohexanethiol (20 mol%, 0.040 mmol, 4.9 μL), DMSO (2mL, 0.1 M). The crude reaction was purified by silica chromatography (0—100% EtOAc/Hexanes gradient) to give 28 mg (45% yield). The physical and spectral data are consistent with reported values.[6]
^{1}H NMR (400 MHz, CDCl$_3$) δ 7.42 – 7.25 (m, 9H), 7.18 – 7.08 (m, 2H), 5.25 (d, *J* = 8.3 Hz, 1H), 5.12 (d, *J* = 3.0 Hz, 2H), 4.69 (dt, *J* = 8.4, 5.9 Hz, 1H), 3.75 (s, 3H), 3.14 (qd, *J* = 13.9, 5.9 Hz, 2H)
^{13}C NMR (101 MHz, CDCl$_3$) δ 172.1, 155.7, 136.3, 135.8, 129.4, 128.7, 128., 128.33, 128.2, 127.2, 67.1, 54.9, 52.4, 38.3.

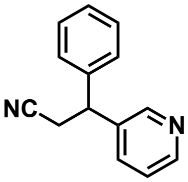

3-phenyl-3-(pyridin-3-yl)propanenitrile (8): Prepared according to general procedure (SI, Section 5). 3-phenyl-3-(pyridin-3-yl)acrylonitrile (41.3 mg, 0.20 mmol), 4CzIPN (1.58 mg, 1 mol%), sodium formate (41 mg, 3 equiv.), cyclohexanethiol (20 mol%, 0.040 mmol, 4.9 μL), DMSO (2mL, 0.1 M). The crude reaction was purified by silica chromatography (0—30% EtOAc/Hexanes gradient) to give 38 mg of an yellow oil (91% yield). The physical and spectral data are consistent with reported values.[7]
^{1}H NMR (400 MHz, CDCl$_3$) δ 8.62 – 8.55 (m, 2H), 7.64 (dt, *J* = 8.0, 1.8 Hz, 1H), 7.43 – 7.21 (m, 6H), 4.46 (t, *J* = 7.6 Hz, 1H), 3.10 (d, *J* = 7.6 Hz, 2H).
^{13}C NMR (101 MHz, CDCl$_3$) δ 148.5, 148.2, 139.7, 137.1, 135.7, 129.2, 127.9, 127.4, 123.9, 117.7, 44.8, 24.0.

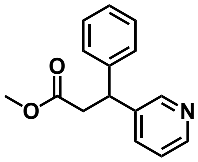

methyl 3-phenyl-3-(pyridin-3-yl)propanoate (9): Prepared according to general procedure (SI, Section 5). methyl (Z)-3-phenyl-3-(pyridin-2-yl)acrylate (48 mg, 0.20 mmol), 4CzIPN (1.58 mg, 1 mol%), sodium formate (41 mg, 3 equiv.), cyclohexanethiol (20 mol%, 0.040 mmol, 4.9 μL), DMSO (2mL, 0.1 M). The crude reaction was purified by silica chromatography (0—100% EtOAc/Hexanes gradient) to give 44 mg (91% yield).
^{1}H NMR (400 MHz, CDCl$_3$) δ 8.54 (d, *J* = 2.3 Hz, 1H), 8.45 (dd, *J* = 4.8, 1.6 Hz, 1H), 7.52 (dt, *J* = 7.9, 2.0 Hz, 1H), 7.33 – 7.24 (m, 2H), 7.24 – 7.17 (m, 4H), 4.58 (t, *J* = 8.0 Hz, 1H), 3.59 (s, 3H), 3.08 (dd, *J* = 8.0, 1.6 Hz, 2H).
^{13}C NMR (101 MHz, CDCl$_3$) δ 171.8, 149.4, 148.1, 142.3, 138.9, 135.2, 128.9, 127.6, 127.1, 123.6, 51.9, 44.7, 40.2.
HRMS (APCI) *m/z:* [M+H]+ calculated for $C_{15}H_{16}O_2N$, 242.1175, found 242.1171.

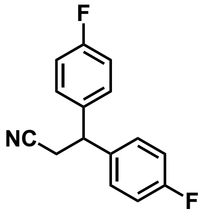

3,3-bis(4-fluorophenyl)propanenitrile (10):
Prepared according to general procedure (SI, Section 5). 3,3-bis(4-fluorophenyl)acrylonitrile (42.2 mg, 0.20 mmol), 4CzIPN (1.58 mg, 1 mol%), sodium formate (41 mg, 3 equiv.), cyclohexanethiol (20 mol%, 0.040 mmol, 4.9 μL), DMSO (2mL, 0.1 M). The crude reaction was purified by silica chromatography (0—70% EtOAc/Hexanes gradient) to give 44 mg of a colorless oil (90% yield).
^{1}H NMR (400 MHz, CDCl$_3$) δ 7.27 – 7.16 (m, 4H), 7.11 – 7.00 (m, 4H), 4.39 (t, *J* = 7.6 Hz, 1H), 3.02 (d, *J* = 7.6 Hz, 2H).
^{13}C NMR (101 MHz, CDCl$_3$) δ 162.1 (d, *J* = 246.8 Hz), 136.9 (d, *J* = 3.3 Hz), 129.2 (d, *J* = 8.1 Hz), 118.1, 116.2 (d, *J* = 21.6 Hz), 45.8, 24.6.
^{19}F NMR (376 MHz, CDCl$_3$) δ -114.68 (tt, *J* = 8.4, 5.1 Hz).
HRMS (APCI) *m/z:* [M+H]+ calculated for $C_{15}H_{12}NF_2$, 244.0932, found 244.0930

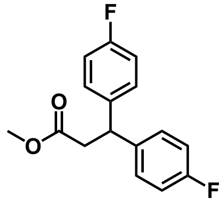

Methyl 3,3-bis(4-fluorophenyl)propanoate (11): Prepared according to general procedure (SI, Section 5). methyl 3,3-bis(4-fluorophenyl)acrylate (55 mg, 0.20 mmol), 4CzIPN (1.58 mg, 1 mol%), sodium formate (41 mg, 3 equiv.), cyclohexanethiol (20 mol%, 0.040 mmol, 4.9 μL), DMSO (2mL, 0.1 M). The crude reaction was purified by silica chromatography (0—20% EtOAc/Hexanes gradient) to give 49 mg of a colorless oil (89% yield). The physical and spectral data are consistent with reported values.[8]
^{1}H NMR (400 MHz, CDCl$_3$) δ 7.24 – 7.14 (m, 4H), 7.05 – 6.95 (m, 4H), 4.55 (t, *J* = 8.0 Hz, 1H), 3.61 (s, 3H), 3.03 (d, *J* = 8.0 Hz, 2H).
^{13}C NMR (101 MHz, CDCl$_3$) δ 171.9, 161.5 (d, *J* = 245.3 Hz), 139.0 (d, *J* = 3.3 Hz), 129.0 (d, *J* = 8.0 Hz), 115.5 (d, *J* = 21.3 Hz), 51.8, 45.4, 40.7.

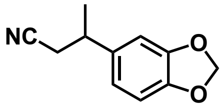

3-(benzo[*d*][1,3]dioxol-5-yl)butanenitrile (12): Prepared according to general procedure (SI, Section 5). 3-(benzo[d][1,3]dioxol-5-yl)but-2-enenitrile (38 mg, 0.20 mmol), 4CzIPN (1.58 mg, 1 mol%), sodium formate (41 mg, 3 equiv.), cyclohexanethiol (20 mol%, 0.040 mmol, 4.9 μL), DMSO (2mL, 0.1 M). The crude reaction was purified by silica chromatography (0—100% EtOAc/Hexanes gradient) to give 35.2 mg of a slight yellow oil (93% yield).
^{1}H NMR (400 MHz, CDCl$_3$) δ 6.83 – 6.68 (m, 3H), 5.97 (s, 2H), 3.11 (h, *J* = 7.0 Hz, 1H), 2.64 – 2.47 (m, 2H), 1.43 (d, *J* = 7.0 Hz, 3H).
^{13}C NMR (101 MHz, CDCl$_3$) δ 148.1, 146.8, 137.1, 119.8, 118.6, 108.6, 106.9, 101.2, 36.4, 26.7, 20.9.
HRMS (APCI) *m/z:* [M+H]+ calculated for $C_{11}H_{12}O_2N$, 190.0862, found 190.0860

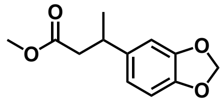

Methyl 3-(benzo[*d*][1,3]dioxol-5-yl)butanoate (13): Prepared according to general procedure (SI, Section 5). methyl (E)-3-(benzo[d][1,3]dioxol-5-yl)but-2-enoate (44 mg, 0.20 mmol), 4CzIPN (1.58 mg, 1 mol%), sodium formate (41 mg, 3 equiv.), cyclohexanethiol (20 mol%, 0.040 mmol, 4.9 μL), DMSO (2mL, 0.1 M). The crude reaction was purified by silica chromatography (0—100% EtOAc/Hexanes gradient) to give 23.3 mg of an oil (62% yield).
^{1}H NMR (400 MHz, CDCl$_3$) δ 6.82 – 6.65 (m, 3H), 5.92 (s, 2H), 3.62 (s, 3H), 3.21 (h, *J* = 7.2 Hz, 1H), 2.53 (qd, *J* = 15.1, 7.5 Hz, 2H), 1.25 (d, *J* = 7.0 Hz, 3H).
^{13}C NMR (101 MHz, CDCl$_3$) δ 172.9, 147.7, 146.0, 139.8, 119.7, 108.3, 107.2, 100.9, 51.6, 43.0, 36.3, 22.1.
HRMS (APCI) *m/z:* [M+H]+ calculated for $C_{12}H_{14}O_4$, 222.0886, found 222.088

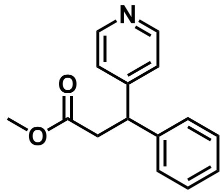

methyl 3-phenyl-3-(pyridin-4-yl)propanoate (14):
Prepared according to general procedure (SI, Section 5 methyl-3-phenyl-3-(pyridin-4-yl)acrylate (48 mg, 0.20 mmol), 4CzIPN (1.58 mg, 1 mol%), sodium formate (41 mg, 3 equiv.), cyclohexanethiol (20 mol%, 0.040 mmol, 4.9 μL), DMSO (2mL, 0.1 M). The crude reaction was purified by silica chromatography (0—80% EtOAc/Hexanes gradient) to give 44 mg of a yellow oil (91% yield).
^{1}H NMR (400 MHz, CDCl$_3$) δ 8.53 – 8.47 (m, 2H), 7.31 (tt, *J* = 6.9, 1.1 Hz, 2H), 7.26 – 7.18 (m, 3H), 7.17 – 7.12 (m, 2H), 4.53 (t, *J* = 7.9 Hz, 1H), 3.60 (s, 3H), 3.14 – 2.99 (m, 2H).
^{13}C NMR (101 MHz, CDCl$_3$) δ 171.8, 152.3, 150.1, 141.8, 129.0, 127.8, 127.3, 123.0, 52.0, 46.4, 39.8.
HRMS (APCI) *m/z:* [M+H]+ calculated for $C_{15}H_{16}O_2N$, 242.1175, found 242.1175.

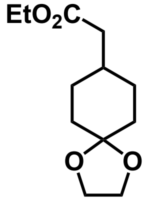

ethyl 2-(1,4-dioxaspiro[4.5]decan-8-yl)acetate (15): Prepared according to general procedure (SI, Section 5). Ethyl 2-(1,4-dioxaspiro[4.5]decan-8-ylidene)acetate (45 mg, 0.20 mmol), 4CzIPN (1.58 mg, 1 mol%), sodium formate (41 mg, 3 equiv.), cyclohexanethiol (20 mol%, 0.040 mmol, 4.9 μL), DMSO (2mL, 0.1 M). The crude reaction was purified by silica chromatography (0—50% EtOAc/Hexanes gradient) to give 28 mg of a slight yellow oil (61% yield). An analytical sample was obtained by preparative TLC (30% EtOAc in hexanes). The physical and spectral data are consistent with reported values.[9]
^{1}H NMR (400 MHz, CDCl$_3$) δ 4.11 (q, *J* = 7.2 Hz, 2H), 3.92 (d, *J* = 2.1 Hz, 4H), 2.21 (d, *J* = 7.1 Hz, 2H), 1.83 (tdd, *J* = 13.7, 6.6, 2.7 Hz, 1H), 1.75 – 1.69 (m, 4H), 1.63 – 1.49 (m, 2H), 1.36 – 1.27 (m, 2H), 1.24 (t, *J* = 7.1 Hz, 3H).
^{13}C NMR (101 MHz, CDCl$_3$) δ 173.0, 108.7, 64.3, 60.3, 41.1, 34.3, 33.5, 30.0, 14.4.

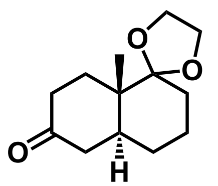

(4a*S*,8a*S*)-8a-methylhexahydro-2*H*-spiro[naphthalene-1,2'-[1,3]dioxolan]-6(5*H*)-one (16): Prepared according to general procedure (SI, Section 5). Ethyl 2-(1,4-dioxaspiro[4.5]decan-8-ylidene)acetate (44.5 mg, 0.20 mmol), 4CzIPN (1.58 mg, 1 mol%), sodium formate (41 mg, 3 equiv.), cyclohexanethiol (20 mol%, 0.040 mmol, 4.9 μL), DMSO (2mL, 0.1 M). The crude reaction was purified by silica chromatography (0—100% EtOAc/Hexanes gradient) to give 34.5 mg (78% yield) of 16 with a d:1 d.r. The physical and spectral data are consistent with reported values.[10]

^{1}H NMR (400 MHz, CDCl$_3$) δ 4.04 – 3.86 (m, 4H), 2.51 – 2.30 (m, 2H), 2.28 – 2.11 (m, 2H), 2.06 (ddt, *J* = 13.3, 11.3, 4.7 Hz, 1H), 1.98 – 1.88 (m, 1H), 1.84 – 1.64 (m, 2H), 1.62 – 1.45 (m, 2H), 1.38 – 1.24 (m, 2H), 1.24 – 1.17 (m, 3H). Only major diastereomer annotated.

^{13}C NMR (101 MHz, CDCl$_3$) δ 211.6, 112.4, 65.3, 65.1, 44.2, 42.7, 41.8, 41.4, 37.9, 30.2, 28.2, 22.8, 13.1.

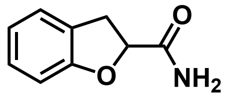

2,3-dihydrobenzofuran-2-carboxamide (17): Prepared according to general procedure (SI, Section 5). benzofuran-2-carboxamide (32.2 mg, 0.20 mmol), 4CzIPN (1.58 mg, 1 mol%), sodium formate (41 mg, 3 equiv.), cyclohexanethiol (20 mol%, 0.040 mmol, 4.9 μL), DMSO (2mL, 0.1 M). The crude reaction was purified by silica chromatography (0—100% EtOAc/Hexanes gradient) to give 23 mg (70% yield). The physical and spectral data are consistent with reported values.[11]

^{1}H NMR (400 MHz, CDCl$_3$) δ 7.24 – 7.11 (m, 2H), 6.92 (td, *J* = 7.5, 1.0 Hz, 1H), 6.86 (d, *J* = 8.0 Hz, 1H), 6.59 (s, 1H), 6.02 (s, 1H), 3.59 (dd, *J* = 16.1, 10.8 Hz, 1H), 3.43 (dd, *J* = 16.1, 6.6 Hz, 1H).

^{13}C NMR (151 MHz, CDCl$_3$) δ 174.5, 158.1, 128.1, 125.1, 125.0, 121.7, 109.5, 80.1, 33.7.

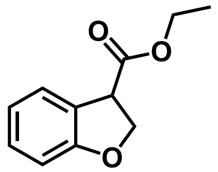

ethyl 2,3-dihydrobenzofuran-3-carboxylate (18): Prepared according to general procedure (SI, Section 5). ethyl benzofuran-3-carboxylate (38 mg, 0.20 mmol), 4CzIPN (1.58 mg, 1 mol%), sodium formate (41 mg, 3 equiv.), cyclohexanethiol (20 mol%, 0.040 mmol, 4.9 μL), DMSO (2mL, 0.1 M). The crude reaction was purified by silica chromatography (0—100% Et_2O/Hexanes gradient) to give 12.6 mg (33% yield). The physical and spectral data are consistent with reported values.[12]

^{1}H NMR (400 MHz, CDCl$_3$) δ 7.49 – 7.37 (m, 1H), 7.21 (dddd, *J* = 8.2, 7.5, 1.5, 0.8 Hz, 1H), 6.96 – 6.81 (m, 2H), 4.96 (dd, *J* = 9.2, 6.7 Hz, 1H), 4.69 (t, *J* = 9.5 Hz, 1H), 4.40 – 4.32 (m, 1H), 4.26 (qq, *J* = 7.2, 3.7 Hz, 2H), 1.33 (t, *J* = 7.1 Hz, 3H).

^{13}C NMR (101 MHz, CDCl$_3$) δ 171.2, 159.9, 129.5, 125.4, 124.4, 120.7, 110.0, 72.5, 61.6, 47.2, 14.3.

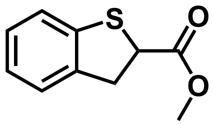

methyl 2,3-dihydrobenzo[*b*]thiophene-2-carboxylate (19): To a vial charged with a stir bar, 38 mg of methyl benzo[b]thiophene-2-carboxylate (0.20 mmol), along with, 4CzIPN (1.58 mg, 1 mol%), sodium formate (41 mg, 3 equiv.), and benzenethiol (20 mol%, 0.040 mmol, 4.1 µL). Finally, 2 mL of DMSO was added. The reaction was then run for 8 hours. We found that cyclohexane thiol resulted in substantial ring opening. However, changing the thiol to PhSH, along with decreasing the reaction time resulted in a cleaner reaction profile. NOTE: The isolated product is unstable and should be used immediately. Pure fractions from a column quickly degraded upon concentration and NMR analysis. For these reasons, we have included this substrate but only report an NMR-yield of 85%. The spectra are consistent with reported literature values.[13]

^{1}H NMR (400 MHz, $CDCl_3$) δ 7.43 – 7.37 (m, 1H), 7.34 – 7.21 (m, 2H), 7.16 (ddd, *J* = 7.4, 6.7, 1.9 Hz, 1H), 4.56 (dd, *J* = 8.8, 5.7 Hz, 1H), 3.86 (s, 3H), 3.83 – 3.76 (m, 2H), 3.64 – 3.53 (m, 1H).
^{13}C NMR (101 MHz, $CDCl_3$) δ 172.4, 139.16, 138.4, 127.78, 124.9, 124.7, 121.8, 52.9, 48.6, 38.3.

CySH Vs. PhSH

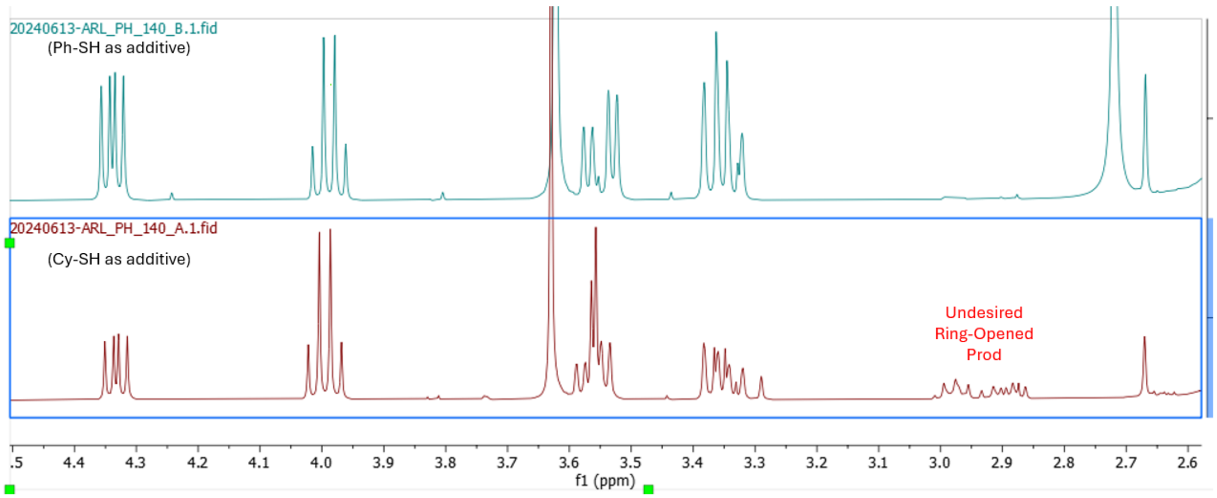

Crude NMR with NMR yield.

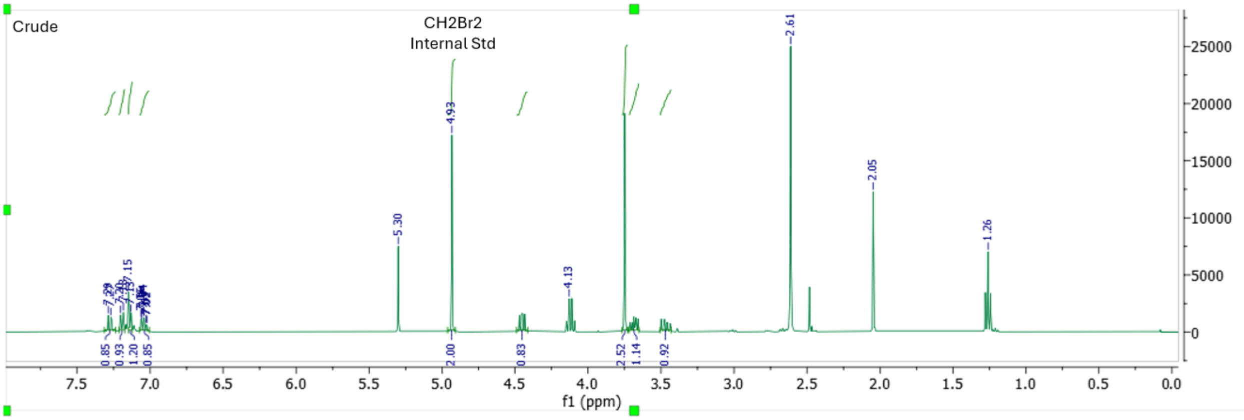

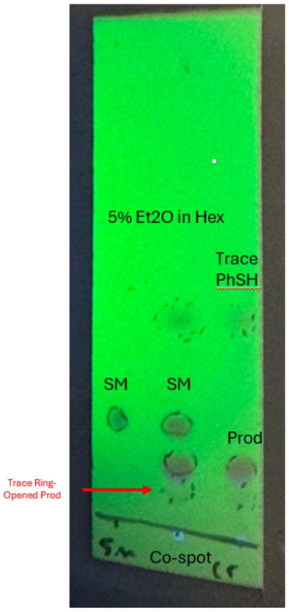

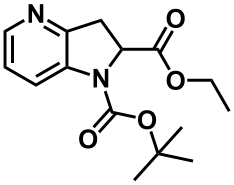

1-(*tert*-butyl) 2-ethyl 2,3-dihydro-1*H*-pyrrolo[3,2-*b*]pyridine-1,2-dicarboxylate (20):Prepared according to general procedure (SI, Section 5). 1-(tert-butyl) 2-ethyl 1H-pyrrolo[3,2-b]pyridine-1,2-dicarboxylate (51.8 mg, 0.20 mmol), 4CzIPN (1.58 mg, 1 mol%), sodium formate (41 mg, 3 equiv.), cyclohexanethiol (20 mol%, 0.040 mmol, 4.9 µL), DMSO (2mL, 0.1 M). The crude reaction was purified by silica chromatography (0—100% EtOAc/Hexanes gradient) to give 50 mg (86% yield).

1g Scale Procedure:

A 20ml scintillation vial was charged with 1-(tert-butyl) 2-ethyl 1H-pyrrolo[3,2-b]pyridine-1,2-dicarboxylate (1.000 g, 1 Eq, 3.444 mmol), sodium formate (702.8 mg, 3 Eq, 10.33 mmol), and 2,4,5,6-tetra(9H-carbazol-9-yl)isophthalonitrile (27.17 mg, .01 Eq, 34.44 µmol). The vial was backfilled with Ar via schlenck line 3x, and DMSO (13.78 mL**, 0.25M**) was added via syringe (DMSO was sparged with Argon for 15min prior to addition). cyclohexanethiol (80.06 mg, 84 µL, 0.2 Eq, 688.9 µmol) was added, and the vial was capped and sealed with parafilm. The vial was placed ~6inches from a 440nm LED and stirred for **24 hours** while being cooled by a fan.

Reaction setup:

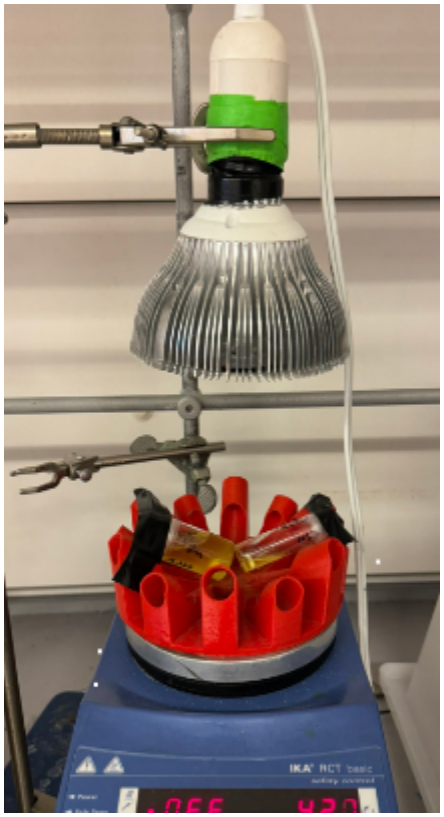

After 24 hours the reaction was diluted with EtOAc (3x20 mls) and washed with water (2x 20mls). The organic layer was separated, washed with Brine (3x20 mls), dried on sodium sulfate, and concentrated in vacuo. The crude material was loaded onto a 100g biotage column and purified with a 0-->80% EtOAc gradient over 10 column volumes. Some trace photocatalyst elutes with this product, but at maximum this corresponds to 2.7% of the isolated product. Isolated 705 mg of **19 (**70%)

TLC in 60% Et2O / Hexanes after 24 hours:

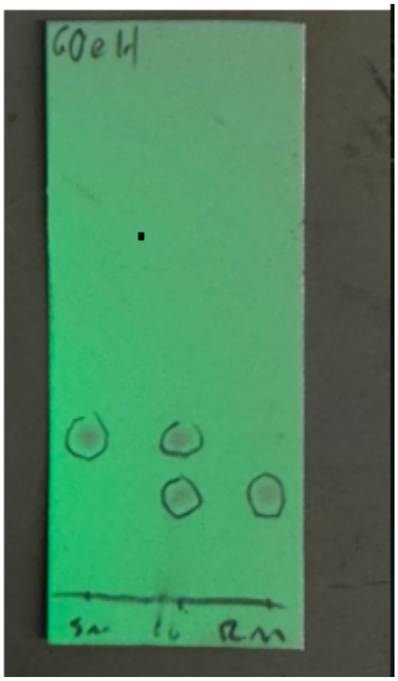

TLC of fractions from column (trace photocatalyst in fractions 36-40):

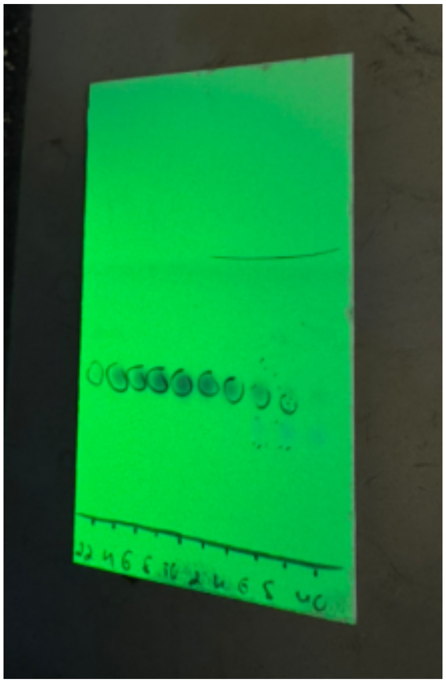

^{1}H NMR (400 MHz, DMSO) δ 8.08 (dd, *J* = 5.0, 1.4 Hz, 1H), 7.98 – 7.80 (m, 1H), 7.23 – 7.15 (m, 1H), 4.91 (dd, *J* = 11.7, 4.4 Hz, 1H), 4.18 (qd, *J* = 7.1, 2.4 Hz, 2H), 3.64 (dd, *J* = 17.5, 11.7 Hz, 1H), 3.11 (dd, *J* = 17.5, 4.5 Hz, 1H), 1.49 (d, *J* = 42.9 Hz, 9H), 1.22 (q, *J* = 5.7 Hz, 3H).
^{13}C NMR (101 MHz, DMSO) δ 171.2, 151.1, 150.9, 142.9, 136.6, 122.1, 119.7, 81.3, 61.2, 58.2, 33.7, 27.6, 14.0.
HRMS (APCI) *m/z:* [M+H]+ calculated for $C_{15}H_{21}O_4N_2$, 293.1495, found 293.1488.

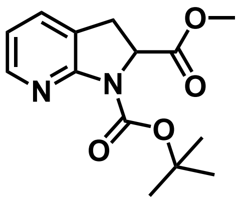

1-(*tert*-butyl) 2-methyl 2,3-dihydro-1*H*-pyrrolo[2,3-*b*]pyridine-1,2-dicarboxylate (21):Prepared according to general procedure (SI, Section 5). **1**-(*tert*-butyl) 2-methyl 1*H*-pyrrolo[2,3-*b*]pyridine-1,2-dicarboxylate (45.3 mg, 0.16 mmol), 4CzIPN (2.59 mg, 2 mol%), sodium formate (41 mg, 4 equiv.), cyclohexanethiol (25 mol%, 0.041 mmol, 5 µL), DMSO (2mL, 0.08 M). The crude reaction was purified by silica chromatography (0—100% Et_2O/Hexanes gradient) to give 43 mg (94% yield).
^{1}H NMR (400 MHz, $CDCl_3$) δ 8.26 (dd, *J* = 5.1, 1.6 Hz, 1H), 7.38 (dd, *J* = 7.4, 1.6 Hz, 1H), 6.84 (dd, *J* = 7.4, 5.1 Hz, 1H), 4.88 (dd, *J* = 11.5, 4.4 Hz, 1H), 3.74 (s, 3H), 3.44 (dd, *J* = 17.0, 11.4 Hz, 1H), 3.05 (dd, *J* = 17.0, 4.4 Hz, 1H), 1.51 (s, 9H).
^{13}C NMR (101 MHz, $CDCl_3$) δ 172.2, 156.5, 150.0, 147.7, 132.7, 121.8, 117.7, 82.2, 59.1, 52.5, 30.1, 28.2.
HRMS (APCI) m/z: [M+H]+ calculated for $C_{14}H_{19}O_4N_2$, 279.1339, found 279.1338

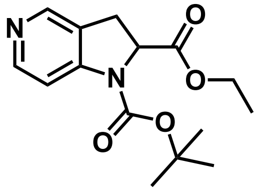

1-(tert-butyl) 2-ethyl 2,3-dihydro-1*H*-pyrrolo[3,2-*c*]pyridine-1,2-dicarboxylate (22):

Prepared according to general procedure (SI, Section 5). 1-(tert-butyl) 2-ethyl 1H-pyrrolo[3,2-c]pyridine-1,2-dicarboxylate (58 mg, 0.20 mmol), 4CzIPN (1.58 mg, 1 mol%), sodium formate (41 mg, 3 equiv.), cyclohexanethiol (20 mol%, 0.040 mmol, 4.9 µL), DMSO (2mL, 0.1 M). The crude reaction was purified by silica chromatography (0—100% EtOAc/Hexanes gradient) to give 53 mg (91% yield).
^{1}H NMR (400 MHz, DMSO) δ 8.32 (d, *J* = 5.4 Hz, 1H), 8.29 (q, *J* = 1.0 Hz, 1H), 7.61 (s, 1H), 4.92 (dd, *J* = 11.6, 4.3 Hz, 1H), 4.17 (q, *J* = 7.2 Hz, 2H), 3.57 (dd, *J* = 16.9, 11.6 Hz, 1H), 3.11 (d, *J* = 16.7 Hz, 1H), 1.49 (d, *J* = 43.8 Hz, 10H), 1.21 (t, *J* = 7.2 Hz, 4H).
^{13}C NMR (101 MHz, DMSO) δ 171.1, 150.7, 149.2, 147.9, 145.5, 124.8, 108.5, 81.7, 61.2, 60.0, 29.7, 27.64, 14.0.
HRMS (APCI)*:* [M+H]+ calculated for $C_{15}H_{21}O_4N_2$, 293.1495, found 293.1491.

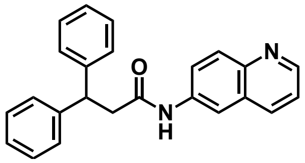

3,3-diphenyl-*N*-(quinolin-6-yl)propenamide (23): Prepared according to general procedure (SI, Section 5). **49** (30.0 mg, 0.086 mmol), 4CzIPN (0.7 mg, 1mol%), sodium formate (17 mg, 3 equiv.), cyclohexanethiol (20 mol%, 0.017 mmol, 2.1 μL), DMSO (1 mL, 0.09 M). The crude reaction was purified by silica chromatography (0--100% EtOAc/Hexanes gradient). 17.6 mg of the title compound was isolated (59% yield) as a clear oil.
^{1}H NMR (400 MHz, CDCl$_3$) δ 8.80 (d, *J* = 4.2 Hz, 1H), 8.24 – 8.18 (m, 1H), 8.06 (dd, *J* = 8.3, 1.7 Hz, 1H), 7.39 – 7.28 (m, 10H), 7.22 (ddt, *J* = 6.9, 4.8, 2.5 Hz, 3H), 4.68 (t, *J* = 7.7 Hz, 1H), 3.17 (d, *J* = 7.7 Hz, 2H).
^{13}C NMR (101 MHz, CDCl$_3$) δ 169.8, 149.5, 145.6, 143.5, 136.0, 135.5, 130.1, 128.9, 128.8, 127.8, 126.9, 123.2, 121.7, 116.4, 47.6, 44.6.
HRMS (APCI) *m/z:* [M+H]+ calculated for $C_{24}H_{21}ON_2$, 353.1643, found 353.1648

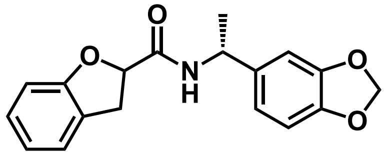

***N*-((*R*)-1-(benzo[*d*][1,3]dioxol-5-yl)ethyl)-2,3-dihydrobenzofuran-2-carboxamide (24):**Prepared according to general procedure (SI, Section 5). **52** (62 mg, 0.20 mmol), 4CzIPN (1.58 mg, 1 mol%), sodium formate (41 mg, 3 equiv.), cyclohexanethiol (20 mol%, 0.040 mmol, 4.9 μL), DMSO (2mL, 0.1 M). The crude reaction was purified by silica chromatography (0—50% Et$_2$O/Hexanes gradient) to give 45 mg (73% yield) 1:1 d.r. as a white solid. The diastereomers were then further separated through careful silica chromatography (0—30% Et$_2$O/Hexanes gradient).
^{1}H NMR (400 MHz, CDCl$_3$) δ 7.23 – 7.13 (m, 2H), 6.93 (td, *J* = 7.4, 1.0 Hz, 1H), 6.86 (d, *J* = 8.0 Hz, 1H), 6.85 – 6.74 (m, 4H), 5.96 (s, 2H), 5.10 (dd, *J* = 10.7, 7.2 Hz, 1H), 5.07 – 5.02 (m, 1H), 3.59 (dd, *J* = 16.3, 10.6 Hz, 1H), 3.44 (td, *J* = 15.8, 7.1 Hz, 1H), 1.42 (d, *J* = 6.9 Hz, 3H).
^{13}C NMR (101 MHz, CDCl$_3$) δ 170.6, 158.4, 148.0, 147.0, 136.8, 128.3, 125.6, 125.3, 121.8, 119.6, 109.8, 108.4, 106.9, 101.2, 80.6, 48.4, 34.0, 21.9.
HRMS (APCI) *m/z:* [M+H]+ calculated for $C_{18}H_{18}O_4N$, 312.1230, found 312.1226.

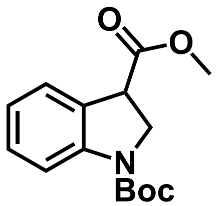

1-(*tert*-butyl) 3-methyl indoline-1,3-dicarboxylate (25):Prepared according to general procedure (SI, Section 5). 1-(*tert*-butyl) 3-methyl 1*H*-indole-1,3-dicarboxylate (55 mg, 0.20 mmol), 4CzIPN (1.58 mg, 1 mol%), sodium formate (41 mg, 3 equiv.), cyclohexanethiol (20 mol%, 0.040 mmol, 4.9 μL), DMSO (2mL, 0.1 M). The crude reaction was purified by silica chromatography (0—100% EtOAc/Hexanes gradient) to give 35 mg (63% yield). The physical and spectral data are consistent with reported values. [14]

^{1}H NMR (400 MHz, CDCl$_3$) δ 7.35 (dq, *J* = 7.5, 1.0 Hz, 1H), 7.30 – 7.19 (m, 1H), 6.96 (td, *J* = 7.5, 1.1 Hz, 1H), 4.39 (dd, *J* = 11.4, 6.0 Hz, 1H), 4.21 (dd, *J* = 10.4, 5.8 Hz, 1H), 4.10 (t, *J* = 11.0 Hz, 1H), 3.78 (s, 3H), 1.57 (s, 9H).
^{13}C NMR (101 MHz, CDCl$_3$) δ 172.0, 152.2, 142.7, 129.1, 125.1, 122.4, 115.0, 81.0, 52.7, 49.9, 28.5.

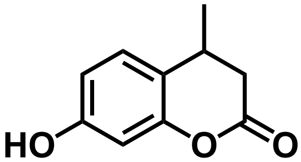

7-hydroxy-4-methylchroman-2-one (26): Prepared according to general procedure (SI, Section 5). Hymecromone (20.0 mg, 0.11 mmol), 4CzIPN (3.6 mg, 4 mol%), sodium formate (42.8 mg, 6 equiv.), cyclohexanethiol (40 mol%, 0.045 mmol, 5.6 µL), DMSO (2mL, 0.06 M). The crude reaction was purified by silica chromatography (0--100% EtOAc/Hexanes gradient). 14.5 mg of the title compound was isolated (72% yield). The physical and spectral data are consistent with reported values.[15] **Note:** Running the reaction with normal formate and photocatalyst loading still produced product but the reaction was slower.
^{1}H NMR (400 MHz, CDCl$_3$) δ 7.08 (dt, *J* = 8.1, 0.7 Hz, 1H), 6.67 – 6.59 (m, 2H), 5.39 (s, 1H), 3.11 (qd, *J* = 7.0, 5.2 Hz, 1H), 2.83 (dd, *J* = 15.8, 5.4 Hz, 1H), 2.55 (dd, *J* = 15.8, 7.5 Hz, 1H), 1.31 (d, *J* = 7.0 Hz, 3H).
^{13}C NMR (101 MHz, CDCl$_3$) δ 168.9, 155.8, 151.9, 127.3, 120.0, 111.8, 104.4, 37.2, 28.9, 20.2.

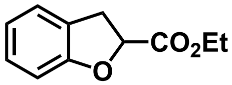

ethyl 2,3-dihydrobenzofuran-2-carboxylate (27): Prepared according to general procedure (SI, Section 5). ethyl benzofuran-2-carboxylate (36 mg, 0.20 mmol), 4CzIPN (1.58 mg, 1 mol%), sodium formate (41 mg, 3 equiv.), cyclohexanethiol (20 mol%, 0.040 mmol, 4.9 µL), DMSO (2mL, 0.1 M). The crude reaction was purified by silica chromatography (0—100% EtOAc/Hexanes gradient) to give 32 mg (82% yield). The physical and spectral data are consistent with reported values.[16]
^{1}H NMR (400 MHz, CDCl$_3$) δ 7.21 – 7.10 (m, 2H), 6.93 – 6.85 (m, 2H), 5.19 (dd, *J* = 10.5, 7.0 Hz, 1H), 4.27 (qd, *J* = 7.1, 1.1 Hz, 2H), 3.56 (ddt, *J* = 15.9, 10.6, 1.1 Hz, 1H), 3.38 (ddt, *J* = 15.9, 7.1, 1.1 Hz, 1H), 1.31 (t, *J* = 7.1 Hz, 3H).
^{13}C NMR (101 MHz, CDCl$_3$) δ 171.3, 159.1, 128.5, 124.8, 124.8, 121.3, 109.9, 79.1, 61.7, 33.9, 14.3.

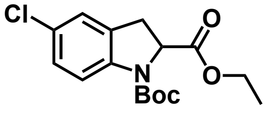

1-(*tert*-butyl) 2-ethyl 5-chloroindoline-1,2-dicarboxylate (28):

Prepared according to general procedure (SI, Section 5). 1-(*tert*-butyl) 2-ethyl 5-chloro-1*H*-indole-1,2-dicarboxylate (58 mg, 0.20 mmol), 4CzIPN (1.58 mg, 1 mol%), sodium formate (41 mg, 3 equiv.), cyclohexanethiol (20 mol%, 0.040 mmol, 4.9 µL), DMSO (2mL, 0.1 M). The crude reaction was purified by silica chromatography (0—100% EtOAc/Hexanes gradient) to give 57 mg (83.8% yield).

^{1}H NMR (400 MHz, DMSO) δ 7.71 (d, *J* = 8.5 Hz, 1H), 7.27 – 7.12 (m, 2H), 4.89 (dd, *J* = 11.6, 4.2 Hz, 1H), 4.16 (q, *J* = 7.1 Hz, 2H), 3.54 (dd, *J* = 17.2, 11.6 Hz, 1H), 3.05 (dd, *J* = 17.1, 4.5 Hz, 1H), 1.42 (s, 9H), 1.21 (t, *J* = 7.1 Hz, 3H).
^{13}C NMR (101 MHz, DMSO) δ 171.3, 150.9, 141.3, 131.1, 127.2, 126.1, 124.8, 114.6, 80.9, 61.0, 60.0, 31.6, 27.6, 14.0.
HRMS (APCI) *m/z:* [M+Na]+ calculated for $C_{16}H_{20}O_4N^{35}Cl^{23}Na$, 348.0973, found 348.0972

8) Preparation of Starting Material/Reagents

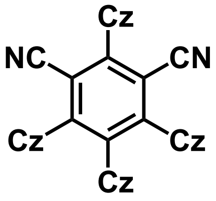

4CzIPN (29): 29 was prepared exactly according to Smith *et al.*[17]

^{1}H NMR (400 MHz, CDCl$_3$) δ 8.22 (d, *J* = 7.7 Hz, 2H), 7.76 – 7.65 (m, 8H), 7.49 (t, *J* = 7.1 Hz, 2H), 7.33 (d, *J* = 7.7 Hz, 2H), 7.27 – 7.19 (m, 4H), 7.14 – 7.03 (m, 8H), 6.82 (t, *J* = 8.2 Hz, 4H), 6.63 (t, *J* = 7.7 Hz, 2H).
^{13}C NMR (101 MHz, CDCl$_3$) δ 145.2, 144.6, 139.9, 138.1, 136.9, 134.7, 126.9, 125.8, 124.9, 124.7, 124.5, 123.8, 122.4, 121.9, 121.4, 121.0, 120.4, 119.6, 116.3, 111.6, 109.9, 109.5, 109.4.

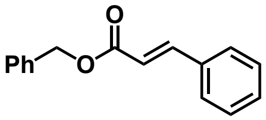

Benzyl cinnamate (30): To a flame dried round bottom flask, 250 mg (1.69 mmol) of cinnamic acid was added followed by 10 mL of DCM (0.17M). Then, 20.6 mg of DMAP (0.17 mmol) was added directly followed by 348 mg (1.69 mmol) of DCC. The reaction stirred for 15 minutes before the addition of benzyl alcohol 0.19 mL, 1.86 mmol). The reaction was monitored by TLC. Once complete, the reaction was quenched with NH_4Cl and extracted with DCM. The organic layer was washed with brine, dried with $MgSO_4$, and concentrated under reduced pressure. The crude material was purified by silica chromatography (0--50% EtOAc/Hexanes gradient) to give 350 mg (87%) of compound 28. The physical and spectral data are consistent with reported values. [18]
^{1}H NMR (400 MHz, CDCl$_3$) δ 7.74 (d, *J* = 16.0 Hz, 1H), 7.57 – 7.48 (m, 2H), 7.46 – 7.30 (m, 8H), 6.50 (d, *J* = 16.0 Hz, 1H), 5.26 (s, 2H).

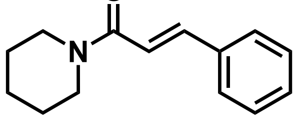

(*E*)-3-phenyl-1-(piperidin-1-yl)prop-2-en-1-one (31):

To a flame dreid round bottom flask charged with a stir bar and under argon, 100 mg of cinnamic acid (0.68 mmol) was added along with 8 mL of anhydrous DCM (0.08 M). 155 mg (0.81 mmol) of 1-(3-

Dimethylaminopropyl)-3-ethylcarbodiimideHydrochloride (EDCI) was added followed by 82.5 mg of DMAP (0.68 mmol). The reaction was allowed to stir for 20 minutes before the addition of piperidine (0.742 mmol, 0.73 μL). The reaction ran overnight before being quenched with saturated NH_4Cl and extracted with DCM. The organic layer was then washed with brine, dried with $MgSO_4$, filtered, and concentrated under reduced pressure. The crude material was purified by silica chromatography (0--100% EtOAc/Hexanes gradient) to give 137 mg (94%) of compound 31. The physical and spectral data are consistent with reported values.[19]
^{1}H NMR (400 MHz, $CDCl_3$) δ 7.64 (d, J = 15.5 Hz, 1H), 7.55 – 7.49 (m, 2H), 7.41 – 7.29 (m, 3H), 6.91 (d, J = 15.4 Hz, 1H), 3.63 (d, J = 31.5 Hz, 4H), 1.71 – 1.64 (m, 2H), 1.64 – 1.59 (m, 4H).

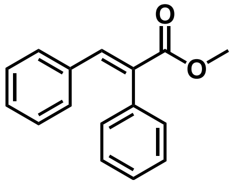

methyl (*E*)-2,3-diphenylacrylate (32): To an oven dried vial, 500 mg (2.23 mmol) of (*E*)-2,3-diphenylacrylic acid was added along with 3mL (0.6 M) of methanol. Then, 0.47 mL of sulfuric acid (8.92 mmol) slowly added. The reaction was refluxed overnight. The reaction was then quenched with sodium bicarbonate, extracted with DCM, dried with $MgSO_4$, filtered, and concentrated under reduced pressure. The crude material was purified by silica chromatography (0--30% EtOAc/Hexanes gradient) to give 520 mg (98%) of compound 32. The physical and spectral data are consistent with reported values.[20]
^{1}H NMR (400 MHz, $CDCl_3$) δ 7.85 (s, 1H), 7.42 – 7.31 (m, 3H), 7.24 – 7.12 (m, 4H), 7.08 – 6.99 (m, 2H), 3.80 (s, 3H).

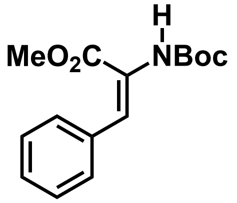

***tert*-butyl-(3-methoxy-3-oxo-1-phenylprop-1-en-2-yl)-λ^2-azanecarboxylate (33):** To a flame dried round bottom flask charged with a stir bar and under argon, 770 mg (2.59 mmol) of methyl 2-((tert-butoxycarbonyl)amino)-2-(dimethoxyphosphoryl)acetatewas added along with 3 ml of DCM (0.8M). DBU was then added to the reaction (2.59 mmol, 0.391 mL). The reaction was allowed to stir for ~15 minutes. Benzaldehyde (250 mg, 0.24 mL, 2.36 mmol) was then added dropwise. The reaction was allowed to run overnight. It was quenched with saturated ammonium chloride, extracted with DCM(x3), dried with $MgSO_4$, filtered, and concentrated under reduced pressure. The crude material was purified by silica chromatography (0--100% EtOAc/Hexanes gradient) to give 381 mg (58%) of compound 33. The physical and spectral data are consistent with reported values.[21]
^{1}H NMR (400 MHz, $CDCl_3$) δ 7.55 – 7.52 (m, 2H), 7.42 – 7.28 (m, 3H), 7.25 (s, 1H), 6.16 (s, 1H), 3.86 (s, 3H), 1.40 (s, 10H).

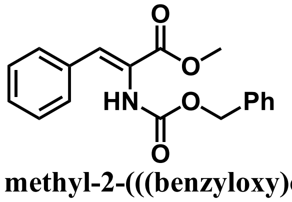

methyl-2-(((benzyloxy)carbonyl)amino)-3-phenylacrylate (34): To a flame dried round bottom flask charged with a stir bar and under argon, 858 mg (2.59 mmol) of methyl 2-(((benzyloxy)carbonyl)amino)-

2-(dimethoxyphosphoryl)acetate was added along with 3 ml of DCM (0.8M). DBU was then added to the reaction (2.59 mmol, 0.391 mL). The reaction was allowed to stir for ~15 minutes. Benzaldehyde (250 mg, 0.24 mL, 2.36 mmol) was then added dropwise. The reaction was allowed to run overnight. It was quenched with saturated ammonium chloride, extracted with DCM (X3), dried with $MgSO_4$, filtered, and concentrated under reduced pressure. The crude material was purified by silica chromatography (0--100% EtOAc/Hexanes gradient) to give 609 mg (83%) of compound 34. The physical and spectral data are consistent with reported values.[21]
^{1}H NMR (400 MHz, $CDCl_3$) δ 7.51 (dd, *J* = 6.8, 2.9 Hz, 2H), 7.42 – 7.27 (m, 9H), 5.12 (s, 2H), 3.82 (s, 3H).

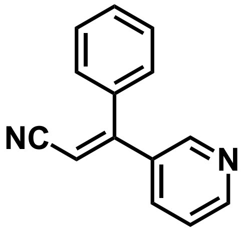

3-phenyl-3-(pyridin-3-yl)acrylonitrile (35):
An oven dried round bottom flask was charged with 531.4 mg (3.00 mmol, 0.486 ml) diethylcyanomethylphosphonate and put under an argon atmosphere. THF (10 ml,0.2 M) was then added followed by potassium tert-butoxide (448 mg, 4.0 mmol). The reaction was stirred for 30 minutes, then phenyl(pyridin-3-yl) methanone(366 mg, 2.0 mmol) was added. The reaction stirred overnight and was quenched with NH_4Cl, extracted with ether, dried with $MgSO_4$, filtered, and concentrated under reduced pressure. The crude material was purified by silica chromatography (0--100% EtOAc/Hexanes gradient) to give 358 mg (86.8%) of compound 35. The physical and spectral data are consistent with reported values. [22]
^{1}H NMR (400 MHz, $CDCl_3$) δ 8.70 (ddd, *J* = 18.7, 4.8, 1.6 Hz, 1H), 8.61 (ddd, *J* = 12.4, 2.4, 0.9 Hz, 1H), 7.71 (dddd, *J* = 99.8, 8.0, 2.4, 1.7 Hz, 1H), 7.50 – 7.27 (m, 6H), 5.82 (d, *J* = 30.8 Hz, 1H).

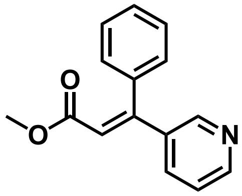

methyl-3-phenyl-3-(pyridin-3-yl)acrylate (36): To an oven-dried flask charged with a stir bar and under argon, 1.37 g (67.5 mmol, 1.21 mL) methyl 2-(dimethoxyphosphoryl)acetate was added along with 16 mL of THF (0.3M). The reaction was cooled to 0 °C and then potassium tert-butoxide 898 mg (8.0 mmol) was carefully added. After 15 minutes, 916 mg (5.00 mmol) of phenyl(pyridin-3-yl)methanone was added. The reaction was allowed to warm to room temp and stirred overnight. The reaction was quenched with saturated ammonium chloride, extracted with EtOAc (X3), dried with $MgSO_4$, filtered, and concentrated under reduced pressure. The crude material was purified by silica chromatography (0--40% EtOAc/Hexanes gradient) to give 1.250g (91.2%) of compound 36. The physical and spectral data are consistent with reported values.[23]
^{1}H NMR (400 MHz, MeOD) δ 8.53 (ddd, *J* = 8.1, 4.9, 1.6 Hz, 1H), 8.43 (dd, *J* = 2.4, 0.9 Hz, 1H), 7.78 – 7.58 (m, 1H), 7.50 – 7.25 (m, 5H), 7.22 – 7.13 (m, 1H), 6.50 (d, *J* = 21.0 Hz, 1H), 3.59 (d, *J* = 7.5 Hz, 3H).

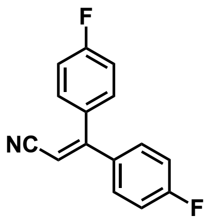

3,3-bis(4-fluorophenyl)acrylonitrile (37): An oven dried round bottom flask was charged with 531.4 mg (3.00 mmol, 0.486 ml) diethylcyanomethylphosphonate and put under an argon atmosphere. THF (10 ml,0.2 M) was then added followed by potassium tert-butoxide (448 mg, 4.0 mmol). The reaction was stirred for 30 minutes, then bis(4-fluorophenyl)methanone (436 mg, 2.0 mmol) was added. The reaction stirred overnight and was quenched with NH_4Cl, extracted with ether, dried with $MgSO_4$, filtered, and concentrated under reduced pressure. The crude material was purified by silica chromatography (0--100% EtOAc/Hexanes gradient) to give 421 mg (87.3%) of compound **37**. The physical and spectral data are consistent with reported values.[24]
^{1}H NMR (400 MHz, $CDCl_3$) δ 7.47 – 7.38 (m, 2H), 7.34 – 7.24 (m, 2H), 7.19 – 7.12 (m, 2H), 7.12 – 7.05 (m, 2H), 5.68 (s, 1H).

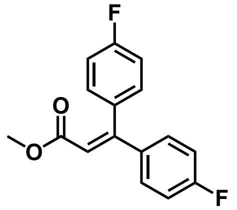

methyl 3,3-bis(4-fluorophenyl)acrylate (38): To an oven-dried flask charged with a stir bar and under argon, 1.37 g (67.5 mmol, 1.21 mL) methyl 2-(dimethoxyphosphoryl)acetate was added along with 16 mL of THF (0.3M). The reaction was cooled to 0 °C and then potassium tert-butoxide 898 mg (8.0 mmol) was carefully added. After 15 minutes, 1.09g (5.00 mmol) of bis(4-fluorophenyl)methanone was added. The reaction was allowed to warm to room temp and stirred overnight. The reaction was quenched with saturated ammonium chloride, extracted with EtOAc (X3), dried with $MgSO_4$, filtered, and concentrated under reduced pressure. The crude material was purified by silica chromatography (0--40% EtOAc/Hexanes gradient) to give 1.250g (91.2%) of compound 38. The physical and spectral data are consistent with reported values. [25]
^{1}H NMR (400 MHz, $CDCl_3$) δ 7.29 – 7.00 (m, 9H), 6.31 (s, 1H), 3.63 (s, 3H).

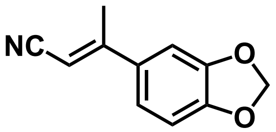

3-(benzo[*d*][1,3]dioxol-5-yl)but-2-enenitrile (39):
To an oven-dried flask charged with a stir bar and under argon, 1.06g (6.00 mmol, 1.19 mL) diethyl (cyanomethyl)phosphonate was added along with 16 mL of THF (0.3M). The reaction was cooled to 0 °C and then sodium hydride 260 mg (6.5 mmol, 60% by mass) was carefully added. After 30 minutes, 820 mg (5.00 mmol) of 1-(benzo[d][1,3]dioxol-5-yl)ethan-1-one was added. The reaction was allowed to warm to room temp and stirred overnight. The reaction was quenched with saturated ammonium chloride, extracted with DCM (X3), dried with $MgSO_4$, filtered, and concentrated under reduced pressure.

The crude material was purified by silica chromatography (0--100% EtOAc/Hexanes gradient) to give 802 mg (85.7%) of compound 39. The physical and spectral data are consistent with reported values. [26]
^{1}H NMR (400 MHz, $CDCl_3$) δ 7.14 – 6.97 (m, 1H), 6.94 (d, *J* = 1.9 Hz, 1H), 6.88 – 6.78 (m, 1H), 6.02 (d, *J* = 1.6 Hz, 2H), 2.42 (d, *J* = 1.0 Hz, 3H).

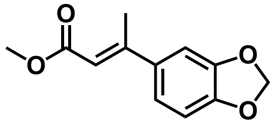

methyl-3-(benzo[*d*][1,3]dioxol-5-yl)but-2-enoate (40): To an oven-dried flask charged with a stir bar and under argon, 1.35g (6.00 mmol, 1.19 mL) of ethyl 2-(diethoxyphosphoryl)acetate was added along with 16 mL of THF (0.3M). The reaction was cooled to 0 °C and then sodium hydride 260 mg (6.5 mmol, 60% by mass) was carefully added. After 30 minutes, 820 mg (5.00 mmol) of 1-(benzo[d][1,3]dioxol-5-yl)ethan-1-one was added. The reaction was allowed to warm to room temp and stirred overnight. The reaction was quenched with saturated ammonium chloride, extracted with DCM (X3), dried with $MgSO_4$, filtered, and concentrated under reduced pressure. The crude material was purified by silica chromatography (0--100% EtOAc/Hexanes gradient) to give 852 mg (77.4%) of compound **40**.
^{1}H NMR (400 MHz, $CDCl_3$) δ 7.05 – 6.94 (m, 2H), 6.80 (dd, *J* = 8.1, 4.1 Hz, 1H), 5.99 (s, 2H), 3.74 (s, 3H), 2.54 (d, *J* = 1.2 Hz, 3H).
^{13}C NMR (101 MHz, $CDCl_3$) δ 167.5, 155.4, 148.5, 148.0, 136.3, 120.6, 115.5, 108.3, 106.8, 101.5, 51.2, 18.1.
HRMS (APCI) *m/z:* [M+H]+ calculated for $C_{12}H_{13}O_4$, 221.0808, found 221.0803

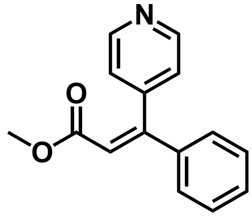

methyl (*Z*)-3-phenyl-3-(pyridin-4-yl)acrylate(41): An oven-dried 20-ml vial was charged with methyl 2-dimethoxyphosphorylacetate (546.3 mg, 463 µL, 3.000 mmol) and dissolved in THF (10.00 mL, 0.19M). KOtBu (448.8 mg, 4.000 mmol) was added at r.t. and the reaction was stirred for 15 min. Phenyl(pyridin-4-yl)methanone 366 mg (2 mmol) was added, and the reaction was heated to 50 °C and stirred overnight (~9 hours). The reaction was quenched with sat. NH_4Cl and extracted with EtOAc. The organic layer was separated, dried on sodium sulfates, and concentrated in vacuo. The crude material was purified by silica chromatography (0--50% EtOAc/Hexanes gradient) to give 244 mg (61%) of compound **41** as a mixture of diastereomers.
^{1}H NMR (400 MHz, $CDCl_3$) δ 8.63 (ddd, *J* = 29.2, 4.5, 1.7 Hz, 2H), 7.45 – 7.31 (m, 3H), 7.27 – 7.23 (m, 2H), 7.21 – 7.12 (m, 2H), 6.46 (d, *J* = 4.1 Hz, 1H), 3.63 (d, *J* = 4.6 Hz, 3H).
^{13}C NMR (101 MHz, $CDCl_3$) δ 165.8, 154.0, 153.9, 150.2, 149.6, 148.0, 147.2, 138.9, 137.2, 130.0, 129.0, 128.7, 128.2, 128.0, 123.7, 122.3, 119.7, 117.9, 51.5.
HRMS (APCI) *m/z:* [M+H]+ calculated for $C_{15}H_{14}O_2N$ 240.1019, found 240.1017

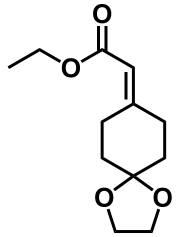

ethyl 2-(1,4-dioxaspiro[4.5]decan-8-ylidene)acetate (42): A flame dried round bottom flask was charged with a stir bar and 15 ml of anhydrous THF. 300 mg of ethyl 2-(diethoxyphosphoryl)acetate (1.34 mmol) was added. The reaction was cooled to 0 °C before the addition of sodium hydride (NaH, 67 mg, 1.67 mmol). Gas evolution was observed. After 15 minutes, 1,4-dioxaspiro[4.5]decan-8-one (261 mg, 1.67 mg) was added. The reaction was allowed to run overnight. The reaction was quenched with aqueous NH_4Cl and extracted with ether. The organic layer was washed with brine, dried with $MgSO_4$, filtered, and concentrated under reduced pressure. The crude material was purified by silica chromatography (0--30% EtOAc/Hexanes gradient) to give 273 mg (73%) of compound **42**. The physical and spectral data are consistent with reported values.[27]
^{1}H NMR (400 MHz, $CDCl_3$) δ 5.66 (q, *J* = 1.2 Hz, 1H), 4.15 (q, *J* = 7.1 Hz, 2H), 4.04 – 3.92 (m, 4H), 3.00 (ddd, *J* = 7.9, 5.0, 1.2 Hz, 2H), 2.38 (ddd, *J* = 8.0, 5.3, 1.2 Hz, 2H), 1.82 – 1.71 (m, 4H), 1.27 (t, *J* = 7.1 Hz, 3H).

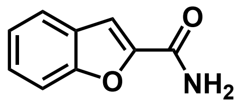

benzofuran-2-carboxamide (43):

An oven dried 20ml scintillation vial was charged with ethyl benzofuran-2-carboxylate (250.0 mg, 1.314 mmol) and calcium chloride (437.6 mg, 3.943 mmol), ammonia (447.7 mg, 3.755 mL, 7 molar, 26.29 mmol) in methanol was added. The vial was capped, heated to 50 °C and stirred for 2 hours and monitored by TLC. The reaction was quenched with H2O and extracted with EtOAc. The organic layer was separated, dried on sodium sulfates, filtered, and concentrated in vacuo. Purified on s column, 0--> 60% EtOAc gradient. The crude material was purified by silica chromatography (0--30% EtOAc/Hexanes gradient) to give 183 mg (86.4%) of compound **43.** The physical and spectral data are consistent with reported values.[28]
^{1}H NMR (400 MHz, $CDCl_3$) δ 7.72 – 7.65 (m, 1H), 7.55 – 7.48 (m, 2H), 7.44 (ddd, *J* = 8.5, 7.1, 1.3 Hz, 1H), 7.35 – 7.26 (m, 1H), 6.56 (s, 1H), 6.08 (s, 1H).

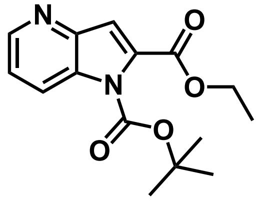

1-(*tert*-butyl) 2-ethyl 1*H*-pyrrolo[3,2-*b*]pyridine-1,2-dicarboxylate (44):
An oven-dried 20ml scintillation vial was charged with ethyl 1H-pyrrolo[3,2-b]pyridine-2-carboxylate (0.200 g, 1 Eq, 1.052 mmol) and dissolved in 5.2mL of ACN. DMAP (12.8mg, 0.1 Eq, 105.2 µmol) was added followed by Boc_2O (0.334 g, 1.5 Eq, 7.89 mmol) The reaction was monitored by TLC, and upon completion was quenched with H2O and extracted with EtOAc. The organic layer was separated, dried on sodium sulfate, and concentrated in vacuo. The crude material was purified by silica chromatography (0--50% EtOAc/Hexanes gradient) to give 257 mg of compound 44 (82.4%). The physical and spectral data are consistent with reported values. [29]
^{1}H NMR (400 MHz, $CDCl_3$) δ 8.59 (dd, *J* = 4.7, 1.4 Hz, 1H), 8.34 (ddd, *J* = 8.5, 1.5, 0.8 Hz, 1H), 7.31 (dd, *J* = 8.5, 4.7 Hz, 1H), 7.21 (d, *J* = 0.8 Hz, 1H), 4.41 (q, *J* = 7.1 Hz, 2H), 1.63 (s, 11H), 1.41 (t, *J* = 7.1 Hz, 3H).

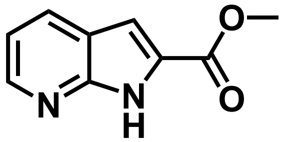

methyl 1*H*-pyrrolo[2,3-*b*]pyridine-2-carboxylate (45): To an oven dried vial, 500 mg (3.08 mmol) of 1H-pyrrolo[2,3-b]pyridine-2-carboxylic acid was added along with 3mL (0.9 M) of methanol. Then, 0.49 mL of sulfuric acid (9.25 mmol) slowly added. The reaction was refluxed overnight. The reaction was then quenched with sodium bicarbonate, extracted with DCM, dried with $MgSO_4$, filtered, and concentrated under reduced pleasure. The crude material was purified by silica chromatography (0--100% EtOAc/Hexanes gradient) to give 221 mg (40%) of compound 45. The physical and spectral data are consistent with reported values. [30]

^{1}H NMR (400 MHz, $CDCl_3$) δ 11.76 (s, 1H), 8.62 (dd, *J* = 4.7, 1.6 Hz, 1H), 7.22 – 7.14 (m, 2H), 4.01 (s, 3H).

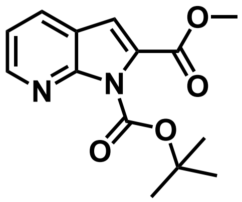

1-(*tert*-butyl) 2-methyl 1*H*-pyrrolo[2,3-*b*]pyridine-1,2-dicarboxylate (46): To an oven dried vial, 100 mg (0.57 mmol) of methyl 1*H*-pyrrolo[2,3-*b*]pyridine-2-carboxylate was added along with 4mL (0.1 M) of acetonitrile. Then, 136 mg (0.62 mmol) of di-tert-butyl decarbonate was added, along with 35 mg (0.284 mmol) of DMAP. The reaction was allowed to stir overnight. The reaction was then quenched with water, extracted with DCM, dried with $MgSO_4$, filtered, and concentrated under reduced pleasure. The crude material was purified by silica chromatography (0--100% EtOAc/Hexanes gradient) to give 77 mg (49%) of compound **46.** The physical and spectral data are consistent with reported values. [29]

^{1}H NMR (400 MHz, $CDCl_3$) δ 8.59 (dd, *J* = 4.7, 1.7 Hz, 1H), 7.96 (dd, *J* = 7.9, 1.7 Hz, 1H), 7.22 (dd, *J* = 7.9, 4.7 Hz, 1H), 7.09 (s, 1H), 3.94 (s, 3H), 1.63 (s, 9H).

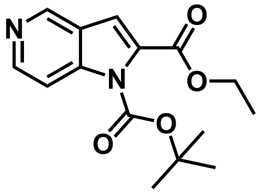

1-(*tert*-butyl) 2-ethyl 1*H*-pyrrolo[3,2-*c*]pyridine-1,2-dicarboxylate (47): An oven dried vial was charged with 1h-pyrrolo[3,2-c]pyridine-2-carboxylic acid etthyl ester (200.0 mg, 1.052 mmol) and dissolved in 1.052 mL THF (1M). DMAP (1.285 mg, 10.52 µmol) was added followed by Boc_2O (252.4 mg, 266 µL, 1.157 mmol). The vial was stirred for ~1hr and monitored by TLC. The reaction was quenched with H2O and extracted with EtOAc. The organic layer was separated, dried on sodium sulfate, filtered, and concentrated in vacuo. The crude material was purified by silica chromatography (0--60% EtOAc/Hexanes gradient) to give 185 mg (60.6%) of compound **47.** The physical and spectral data are consistent with reported values. [29]

^{1}H NMR (400 MHz, $CDCl_3$) δ 8.93 (d, *J* = 1.1 Hz, 1H), 8.54 (d, *J* = 5.9 Hz, 1H), 7.93 (dt, *J* = 5.9, 0.9 Hz, 1H), 7.16 (d, *J* = 0.8 Hz, 1H), 4.40 (q, *J* = 7.1 Hz, 2H), 1.64 (s, 11H), 1.40 (t, *J* = 7.1 Hz, 3H).

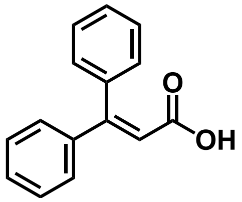

3,3-diphenylacrylic acid (48):
To a flame dried round bottom flask under argon and charged with a stir bar methyl 2-(dimethoxyphosphoryl)acetate (750 mg, 4.21 mmol) was added followed by 30 mL of THF (0.091 M). Then NaH was slowly added (165 mg, 4.12 mmol. 60% w/w) and gas evolution was observed. After 1 hour, benzophenone (500 mg, 2.74 mmol) was added and the reaction was allowed to run overnight. The reaction was then quenched with NH_4Cl and extracted with Et_2O (X3). The organic layer was washed with brine, dried with $MgSO_4$, filtered, and concentrated under reduced pressure. The crude material was then run through a quick silica plug to product an inseparable mixture of methyl 3,3-diphenylacrylate and unreactive benzophenone. This mixture was then suspended in a 2:1 mixture of THF:Water (30 mL). Then, 329 mg (13.7 mmol) of LiOH was added and the reaction was allowed to stir overnight. The reaction was then extracted with DCM. The aqueous layer then acidified with 1M HCl and extract with DCM. The acidified organic layer was then washed with brine, dried with $MgSO_4$, filtered and concentrated under reduced pressure. The resulting compound 48 was isolated in 34% yield (210 mg) and was used without further purification. The physical and spectral data are consistent with reported values. [31]

^{1}H NMR (400 MHz, $CDCl_3$) δ 7.43 – 7.17 (m, 11H), 6.33 (s, 1H).

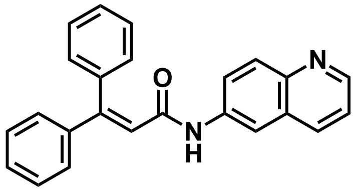

3,3-diphenyl-*N*-(quinolin-6-yl)acrylamide (49):
To a flame dried vial charged with a stir bar and under argon, 35 mg of **48** (0.156 mmol) was added followed by 2 mL of DCM (0.08 M). Then, DMAP (28.6 mg, 0.234 mmol) and EDC (44.9 mg, 0.234 mmol) was added. The reaction stirred for 30 minutes before the addition of quinolin-6-amine (27 mg, 0.187 mmol). The reaction was allowed to run overnight before being quenched with NH_4Cl. The reaction was extracted with DCM (X3), washed with brine, dried with $MgSO_4$, filtered, and concentrated under reduced pressure. The crude material was purified by silica chromatography (0--100% EtOAc/Hexanes gradient) to give 36 mg (66%) of compound **49**.
^{1}H NMR (400 MHz, $CDCl_3$) δ 8.79 (dd, J = 4.2, 1.7 Hz, 1H), 8.24 (d, J = 2.4 Hz, 1H), 8.07 (dt, J = 8.2, 1.2 Hz, 1H), 7.89 (d, J = 9.0 Hz, 1H), 7.52 (dd, J = 5.1, 1.8 Hz, 3H), 7.44 – 7.34 (m, 8H), 7.09 (s, 1H), 6.82 (dd, J = 9.0, 2.4 Hz, 1H), 6.57 (s, 1H).
^{13}C NMR (101 MHz, $CDCl_3$) δ 185.1, 164.5, 151.1, 140.2, 138.0, 135.4, 130.0, 129.5, 129.4, 129.2, 129.2, 128.6, 128.1, 122.7, 121.5, 115.6.
HRMS (APCI) *m/z:* [M+H]+ calculated for $C_{24}H_{19}ON_2$, 351.1492, found 351.14889.

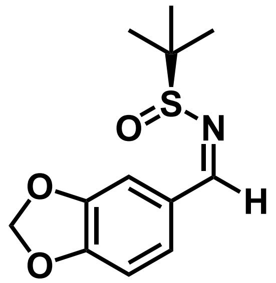

(S,Z)-N-(benzo[d][1,3]dioxol-5-ylmethylene)-2-methylpropane-2-sulfinamide (50):
A 3-neck 500ml round bottom flask was charged with a stir bar and flame dried under vaccum. The flask was then charged with Cs2CO3 (26.04 g, 1.20 Eq, 79.93 mmol), (S)-2-methylpropane-2-sulfinamide (8.477 g, 1.05 Eq, 69.94 mmol), and benzo[d][1,3]dioxole-5-carbaldehyde (10.00 g, 1 Eq, 66.61 mmol). DCM (222.0 mL) was added, and a water condenser was equipped to the flask. The flask was heated to 37 °C to maintain a gentle reflux. The reaction was stirred at this temp for at least 12 hours and monitored by 1H-NMR. The reaction was deemed complete when <5% remaining aldehyde was observed. Upon completion, the reaction was cooled to room temp and quenched with a saturated solution of NH_4Cl and extracted with DCM. The organic layer was separated, washed with saturated brine, dried on sodium sulfate, and concentrated in vacuo. The crude material was typically not purified and carried forward directly in the next step.
^{1}H NMR (400 MHz, $CDCl_3$) δ 8.44 (d, J = 1.0 Hz, 1H), 7.40 (d, J = 1.6 Hz, 1H), 7.32 – 7.24 (m, 1H), 6.88 (dd, J = 8.0, 1.1 Hz, 1H), 6.04 (q, J = 1.3 Hz, 2H), 1.24 (d, J = 1.1 Hz, 9H).
^{13}C NMR (101 MHz, $CDCl_3$) δ 161.7, 151.5, 148.6, 129.2, 127.0, 108.5, 107.3, 101.9, 57.7, 22.6.
HRMS (APCI) *m/z:* : [M+H]+ calculated for $C_{12}H_{16}O_3N^{32}S$, 254.0845, found 254.0833

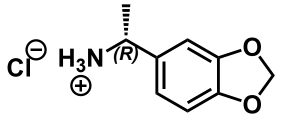

(R)-1-(benzo[d][1,3]dioxol-5-yl)ethan-1-amiminum chloride (51):
An oven-dried two neck flask was charged w/ (S,Z)-N-(benzo[d][1,3]dioxol-5-ylmethylene)-2-methylpropane-2-sulfinamide (1.060 g, 1 Eq, 4.184 mmol) and dissolved in DCM (20.92 mL). The solution was cooled to 0 °C via ice bath, then methyl magnesium bromide (997.9 mg, 2.790 mL, 3.0 molar, 2 Eq, 8.369 mmol) was added dropwise via syringe. The reaction was stirred for 2 hours at this temp, then removed from the ice bath and slowly allowed to come to room temp and stirred o/n. The reaction was carefully quenched with NH4Cl and 0°C and diluted with EtOAc. The organic layer was separated, washed with brine, dried on sodium sulfate, and concentrated in vacuo. The resulting yellow powder was dissolved in a methanolic solution of HCl (1.526 g, 13.95 mL, 3 molar, 10 Eq, 41.84 mmol) and stirred for 1hour. The methanol was removed *in vacuo*, and the resulting crude HCl salt was dissolved in diethyl ether. The white precipitate was filtered off and washed with additional cold Et_2O to yield the pure HCl salt.
^{1}H NMR (400 MHz, DMSO) δ 8.55 (s, 3H), 7.18 (d, J = 1.8 Hz, 1H), 6.98 (dt, J = 8.2, 2.0 Hz, 1H), 6.93 (d, J = 8.0 Hz, 1H), 6.02 (p, J = 1.2 Hz, 2H), 4.29 (q, J = 6.7 Hz, 1H), 1.47 (dd, J = 6.8, 2.3 Hz, 3H).
^{13}C NMR (151 MHz, DMSO) δ 147.42, 147.13, 133.23, 120.62, 108.24, 107.36, 101.20, 49.85, 20.82.
HRMS (ESI Positive) *m/z:* [M-NH_3]+ calculated for $C_9H_9O_2$, 149.0597, found 149.0597.

NOTE: Despite testing a clarity of HRMS and LC-MS conditions, we exclusory observed the [M-NH_3]$^+$ ion. We note that the same racemic hydrochloride acid has been characterized previously and our ^{1}H and ^{13}C are consistent with this report. [32]

To characterize enantiopurity, a 50 mg sample of the corresponding tert-butyl carbamate was prepared. A ~1mg / ml sample was prepared in 10% isopropyl alcohol in hexanes and analyzed via chiral HPLC.

```
=====================================================================
Acq. Operator   : SYSTEM                          Seq. Line :   2
Sample Operator : SYSTEM
Acq. Instrument : Normal Phase                     Location :   P2-E-01
Injection Date  : 10/10/2023 5:40:17 PM                 Inj :   1
                                                Inj Volume : 5.000 µl
Method          : C:\Users\Public\Documents\ChemStation\1\Data\CHIRAL_SCREENS\2023-10-10 17-28-
                  51 10%IPA_SCREEN_30min\10_IPA_IA_FIXED_1.0mL-min_30min.M (Sequence Method)
Last changed    : 10/1/2022 2:18:02 PM by SYSTEM
Method Info     : Date Created: 09/27/2022
                  Created By: Patrick Gross
                  %IPA: 10
                  Column: IA
                  Flowrate: 1.0 mL/min
                  Runtime: 30.0 min
                  Wavelengths: 210nm, 230nm, 254nm, 280nm

Additional Info : Peak(s) manually integrated
```

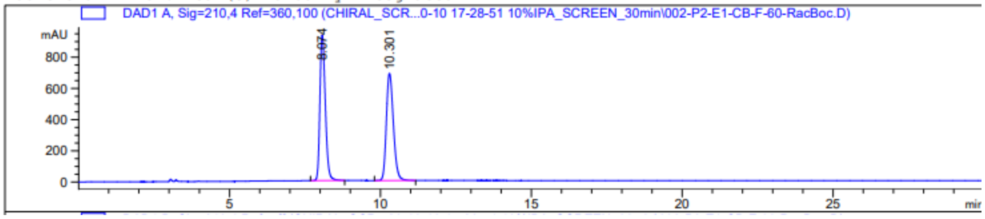


```
=====================================================================
                         Area Percent Report
=====================================================================

Sorted By             :      Signal
Multiplier            :      1.0000
Dilution              :      1.0000
Do not use Multiplier & Dilution Factor with ISTDs


Signal 1: DAD1 A, Sig=210,4 Ref=360,100

Peak RetTime Type  Width     Area      Height     Area
  #   [min]        [min]   [mAU*s]     [mAU]        %
----|-------|----|-------|----------|----------|--------|
   1   8.074 VB R  0.1901 1.15978e4  932.82776  51.1097
   2  10.301 VB R  0.2461 1.10942e4  686.21411  48.8903

Normal Phase 10/11/2023 3:34:54 PM SYSTEM                          Page   2 of 4
```

```
==================================================================
Acq. Operator   : SYSTEM                          Seq. Line :   2
Sample Operator : SYSTEM
Acq. Instrument : Normal Phase                     Location :   P2-E-02
Injection Date  : 10/11/2023 1:36:46 PM                 Inj :   1
                                                Inj Volume : 5.000 µl
Method          : C:\Users\Public\Documents\ChemStation\1\Data\IA_NP_Data\2023-10-11 13-25-20 10
                  %IPA_IA_30min\10_IPA_IA_FIXED_1.0mL-min_30min.M (Sequence Method)
Last changed    : 10/1/2022 2:18:02 PM by SYSTEM
Method Info     : Date Created: 09/27/2022
                  Created By: Patrick Gross
                  %IPA: 10
                  Column: IA
                  Flowrate: 1.0 mL/min
                  Runtime: 30.0 min
                  Wavelengths: 210nm, 230nm, 254nm, 280nm

Additional Info : Peak(s) manually integrated
```

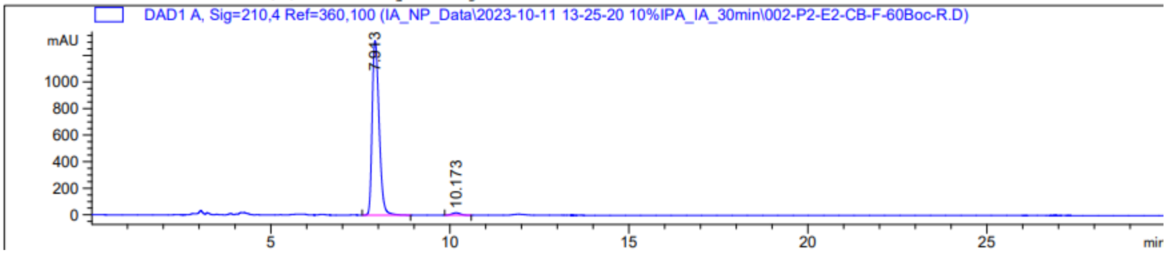


```
==================================================================
                         Area Percent Report
==================================================================

Sorted By             :      Signal
Multiplier            :      1.0000
Dilution              :      1.0000
Do not use Multiplier & Dilution Factor with ISTDs


Signal 1: DAD1 A, Sig=210,4 Ref=360,100

Peak RetTime Type  Width     Area      Height     Area
  #   [min]        [min]   [mAU*s]     [mAU]       %
----|-------|----|-------|----------|----------|--------|
   1   7.913 BV R  0.1832 1.75951e4  1313.99463 98.4497
   2  10.173 BV R  0.1910  277.07864   17.38286  1.5503
```


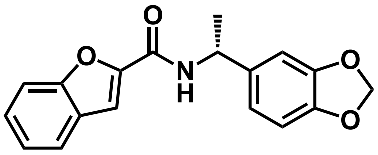

(*R*)-*N*-(1-(benzo[*d*][1,3]dioxol-5-yl)ethyl)benzofuran-2-carboxamide (52): An oven-dried 20-ml vial was charged with a stir bar, benzofuran-2-carboxylic acid (100 mg, 1 Eq, 617 µmol), (R)-(1-(benzo[d][1,3]dioxol-5-yl)ethyl)chloro-l5-azane (124 mg, 1 Eq, 617 µmol), DMAP (15.1 mg, 0.2 Eq, 123 µmol), and EDC (236 mg, 2 Eq, 1.23 mmol). DCM (3.08 mL) was added followed by triethylamine (187 mg, 258 µL, 3 Eq, 1.85 mmol), and the reaction was stirred for 3 hours. The reaction was quenched by the

addition of water and the organic layer was separated, dried on sodium sulfate, and concentrated in vacuo. The crude material was purified by silica chromatography (0--20% EtOAc/Hexanes gradient) to give 125 mg (65.5%) of compound **52.**

^{1}H NMR (400 MHz, CDCl$_3$) δ 7.66 (dt, *J* = 7.8, 1.0 Hz, 1H), 7.52 – 7.45 (m, 2H), 7.41 (ddd, *J* = 8.4, 7.2, 1.3 Hz, 1H), 7.33 – 7.27 (m, 1H), 6.94 – 6.86 (m, 2H), 6.79 (dd, *J* = 7.7, 3.7 Hz, 2H), 5.95 (s, 2H), 5.26 (dt, *J* = 14.3, 7.5 Hz, 1H), 1.61 (d, *J* = 6.9 Hz, 3H).

^{13}C NMR (101 MHz, CDCl$_3$) δ 158.0, 154.8, 148.7, 148.0, 147.0, 136.8, 127.7, 127.0, 123.8, 122.8, 119.7, 111.8, 110.7, 108.5, 107.0, 101.2, 48.7, 22.0.

HRMS (APCI) *m/z:* [M+H]+ calculated for $C_{18}H_{18}O_4N$, 310.1074, found 310.1070.

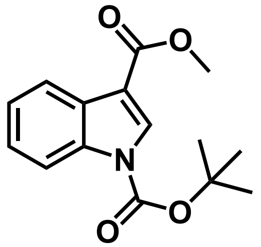

1-(*tert*-butyl) 3-methyl 1*H*-indole-1,3-dicarboxylate (53):

An oven-dried 20ml scintillation vial was charged with methyl 1H-indole-3-carboxylate (184.2 mg, 1 Eq, 1.052 mmol) and dissolved in ACN (5.258 mL). DMAP (12.85 mg, 0.1 Eq, 105.2 μmol) was added at room temp followed by Boc$_2$o (344.2 mg, 362 μL, 1.5 Eq, 1.577 mmol). The reaction was stirred and monitored by TLC (~1 hour). Upon complete consumption of SM, the reaction was diluted with EtOAc and quenched with H_2O. The organic layer was separated, dried on sodium sulfates, and concentrated in vacuo. The crude material was purified by silica chromatography (0--10% EtOAc/Hexanes gradient) to give 257 mg (84.2%) of compound **53.**The physical and spectral data are consistent with reported values. [33]

^{1}H NMR (400 MHz, CDCl$_3$) δ 8.27 (s, 1H), 8.22 – 8.11 (m, 2H), 7.42 – 7.30 (m, 2H), 3.94 (s, 3H), 1.69 (s, 9H).

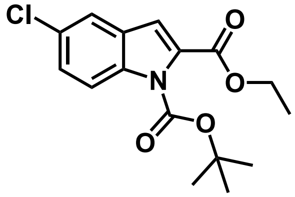

1-(*tert*-butyl) 2-ethyl 5-chloro-1*H*-indole-1,2-dicarboxylate (54): An oven-dried round bottom flask was charged with ethyl 5-chloro-1H-indole-2-carboxylate (1.00 g, 4.47 mmol) and boc2o (1.46 g, 1.54 mL, 6.71 mmol) then dissolved in 22.4 mL CAN (0.2M). DMAP (109 mg, 0.2 Eq, 894 μmol) was added and the reaction was left to stir for ~1 hr. TLC (%10 E/Hex) confirmed complete conversion of SM. The reaction was diluted with EtOAc and quenched with water. The organic layer was separated, dried on sodium sulfate, and concentrated in vacuum. 912 mg (91.6%) of compound 54 was isolated without the need for purification. The physical and spectral data are consistent with reported values. [34]

^{1}H NMR (400 MHz, CDCl$_3$) δ 8.01 (dt, *J* = 8.9, 0.7 Hz, 1H), 7.57 (d, *J* = 2.1 Hz, 1H), 7.36 (dd, *J* = 8.9, 2.1 Hz, 1H), 7.01 (d, *J* = 0.7 Hz, 1H), 4.38 (q, *J* = 7.1 Hz, 2H), 1.62 (s, 9H), 1.40 (t, *J* = 7.1 Hz, 3H).

9) Spectra

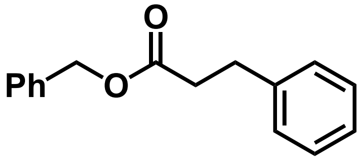

^{1}H NMR (400 MHz, $CDCl_3$), 2

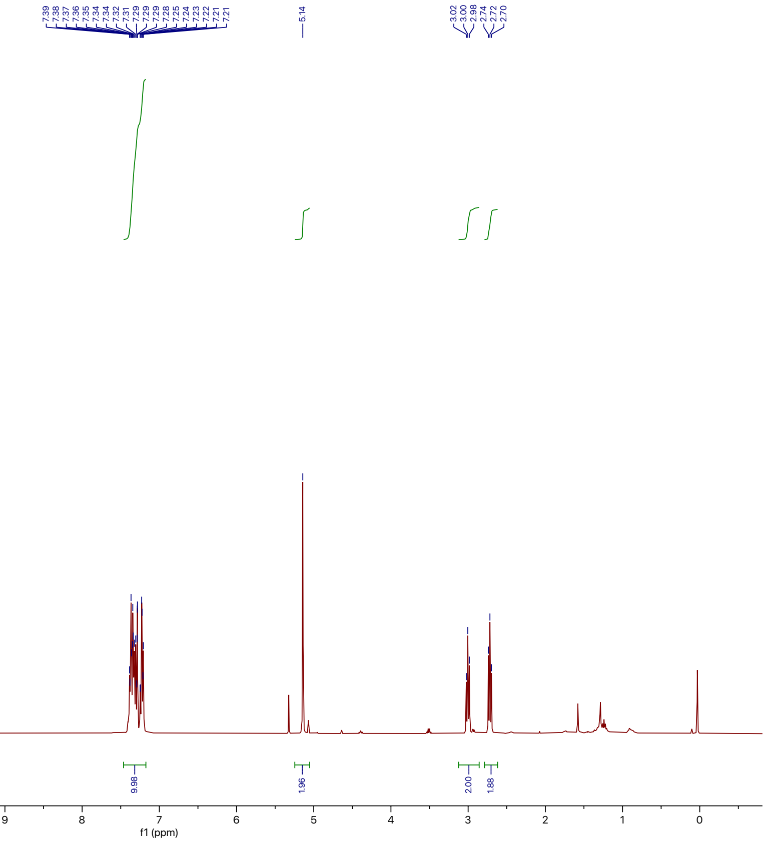

^{13}C NMR (101 MHz, $CDCl_3$), 2

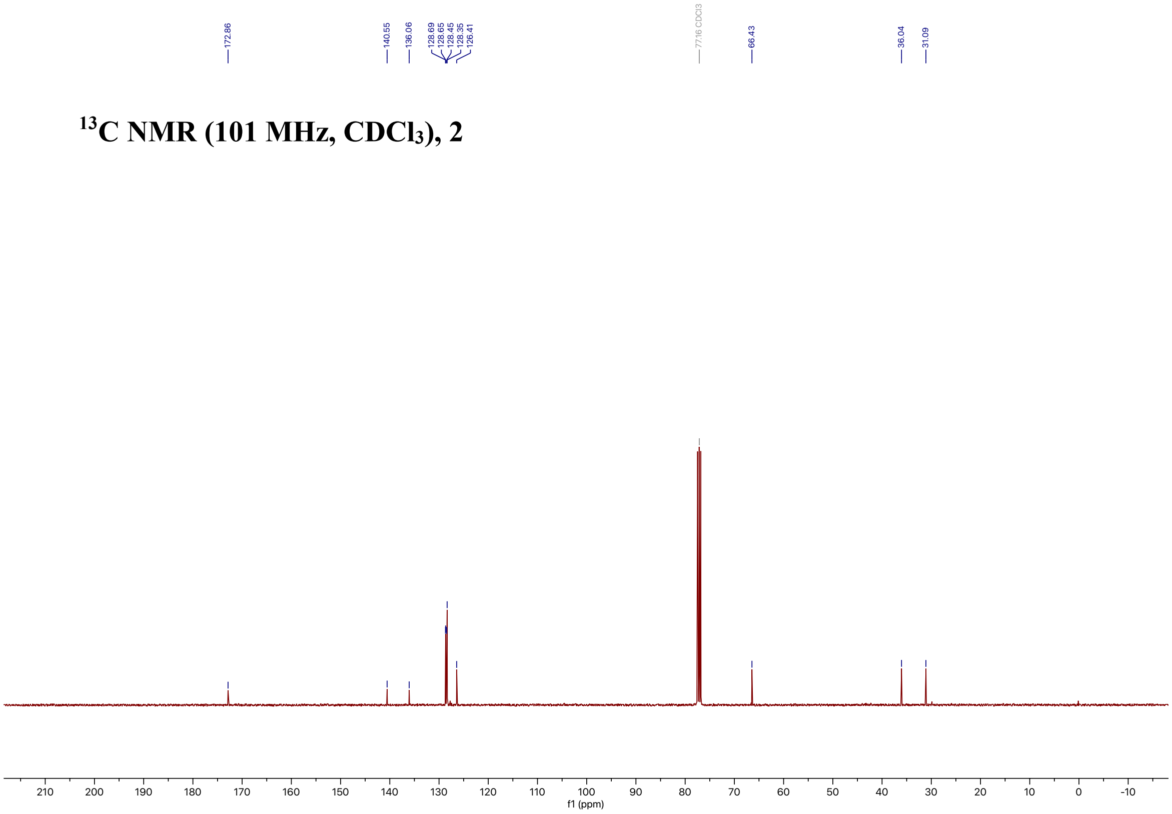

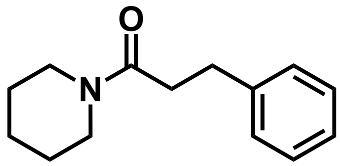

^{1}H NMR (400 MHz, $CDCl_3$), 3

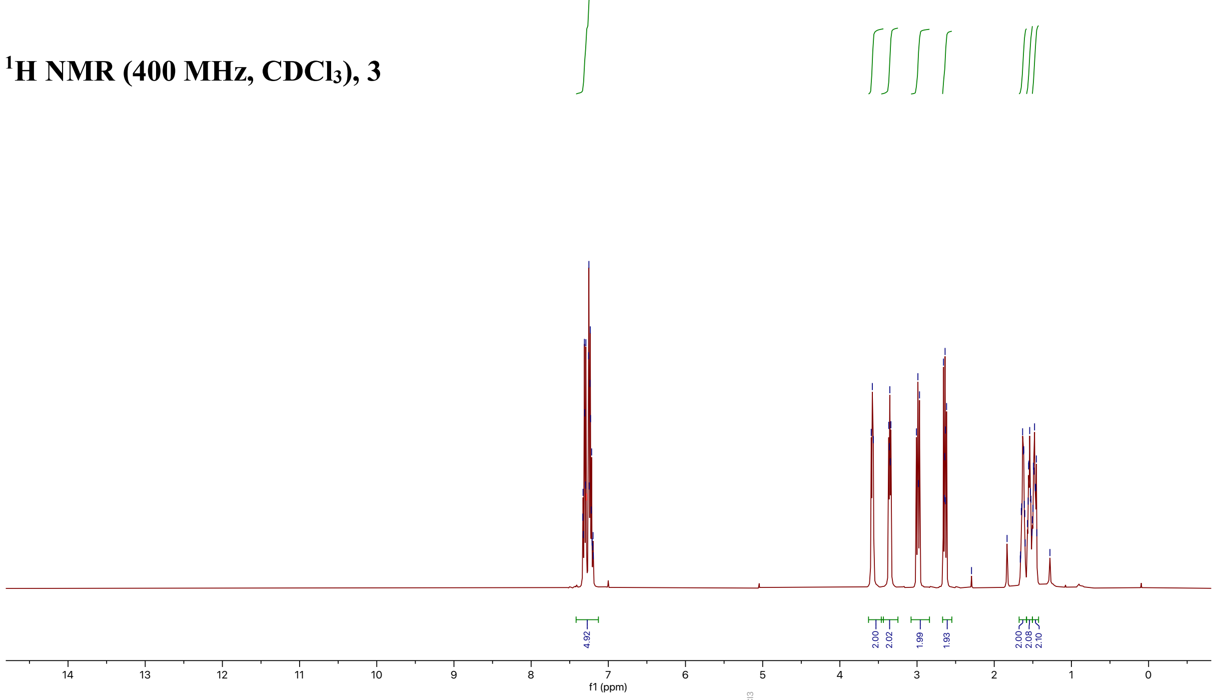

^{13}C NMR (101 MHz, $CDCl_3$), 3

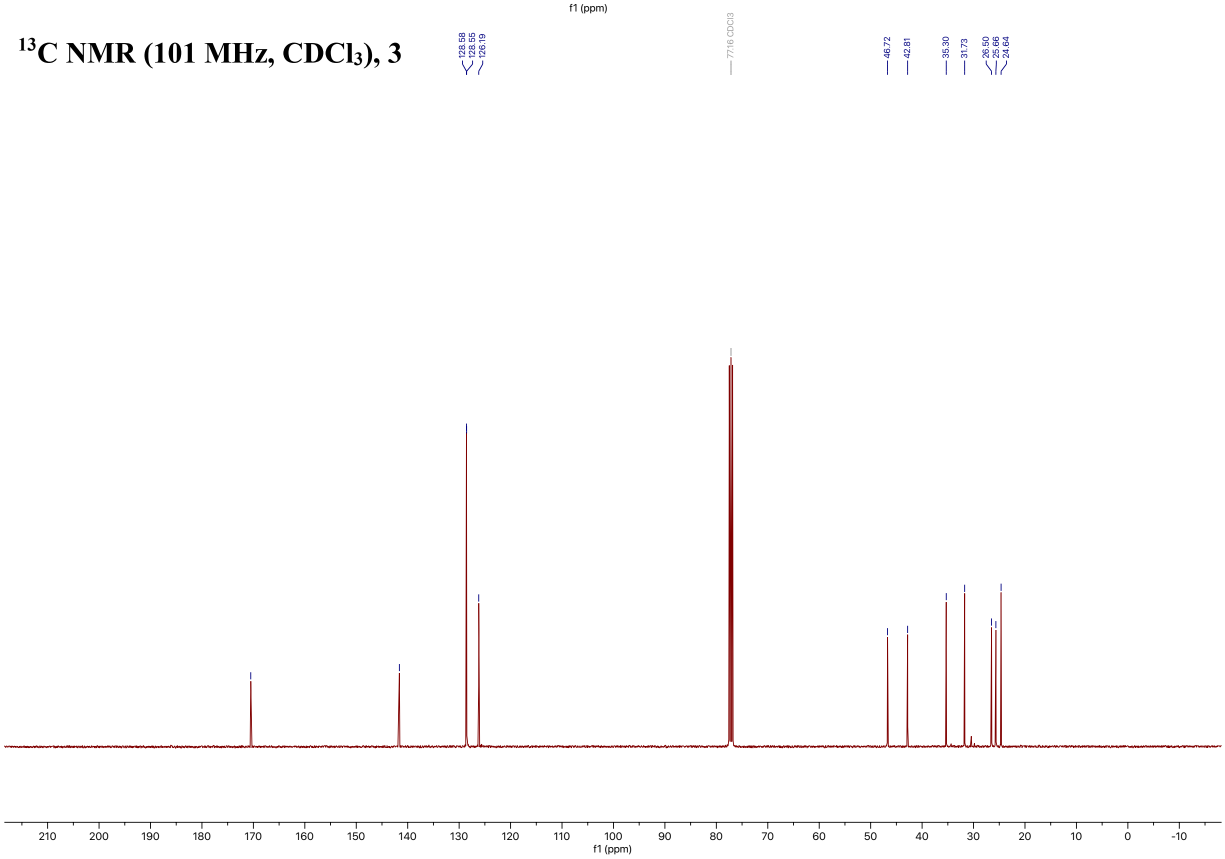

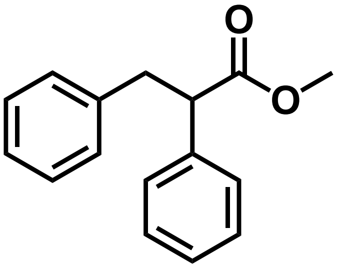

^{1}H NMR (400 MHz, $CDCl_3$), 4

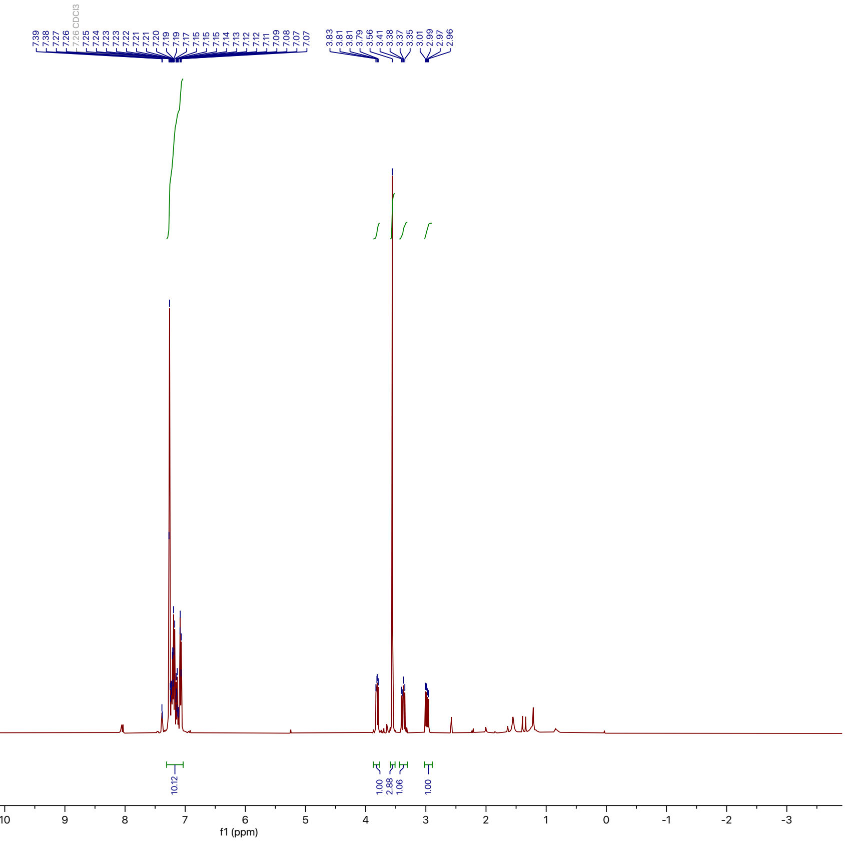

^{13}C NMR (101 MHz, $CDCl_3$), 4

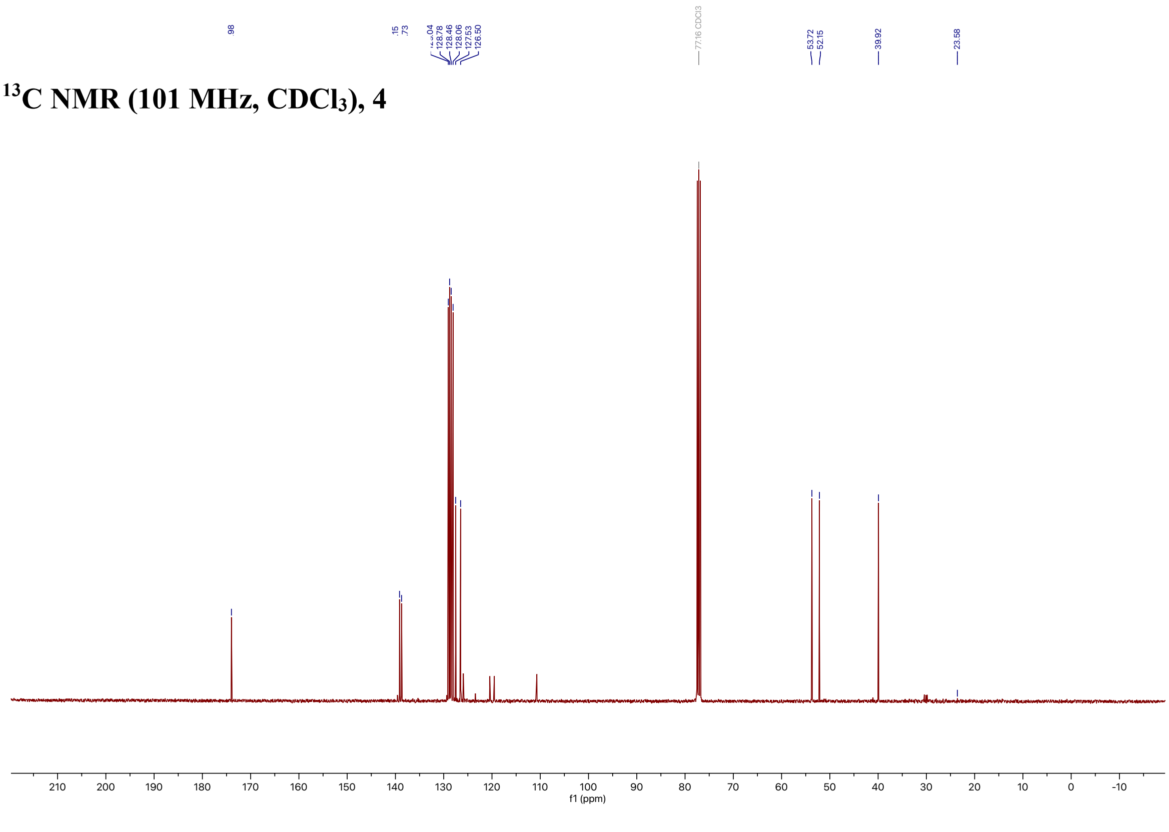

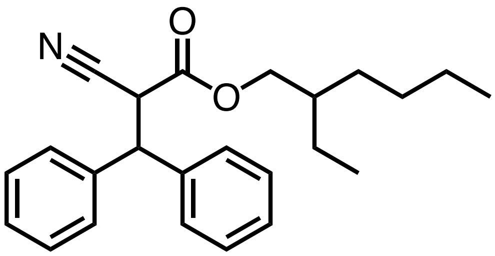

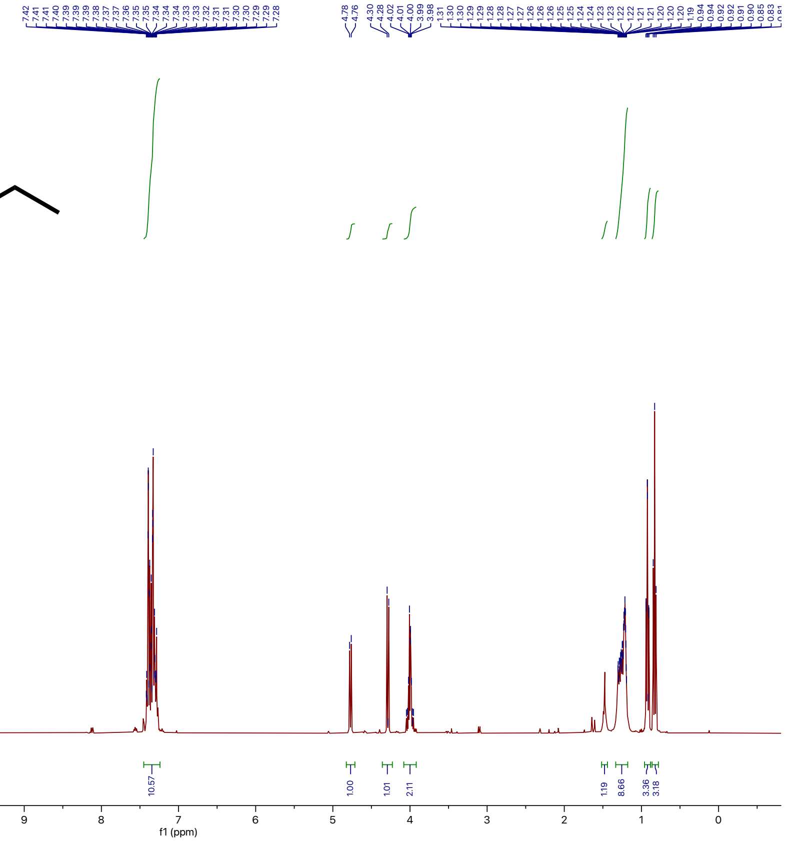

^{1}H NMR (400 MHz, $CDCl_3$), 5

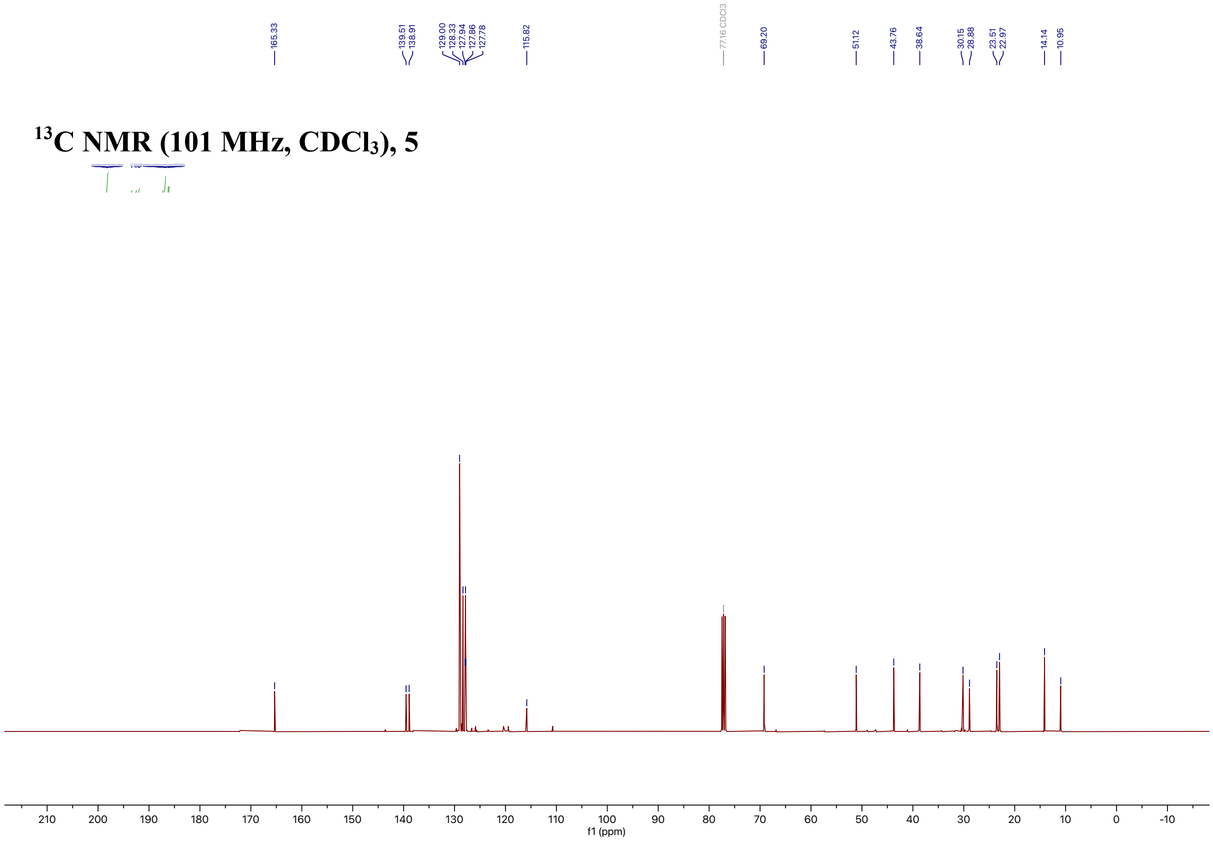

^{13}C NMR (101 MHz, $CDCl_3$), 5

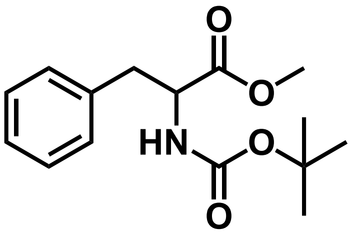

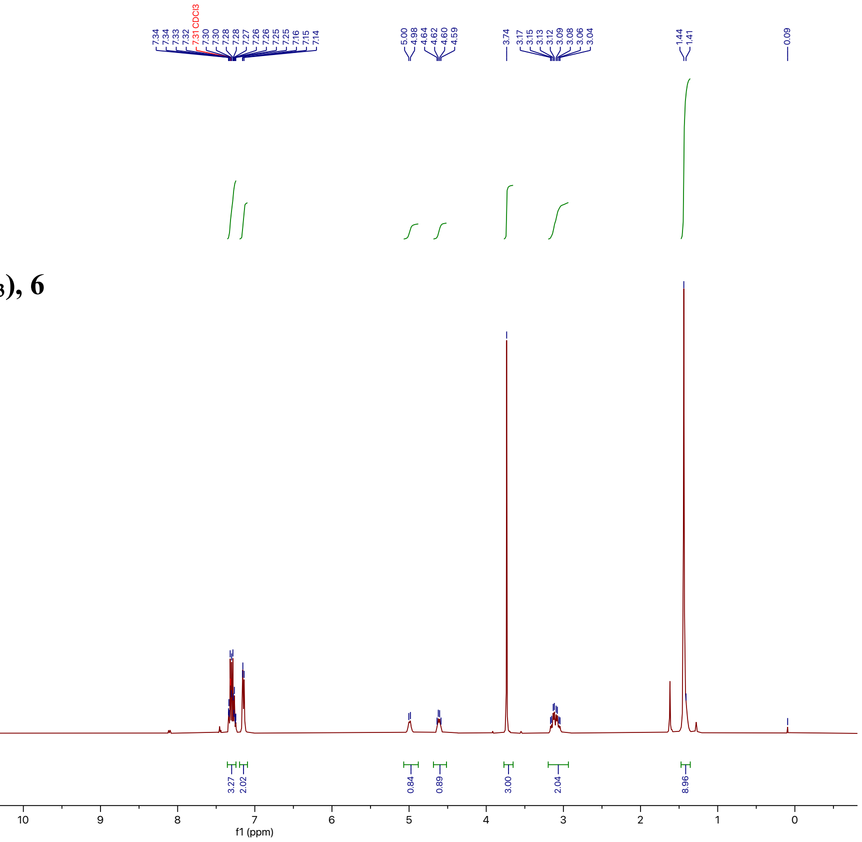

^{1}H NMR (400 MHz, $CDCl_3$), 6

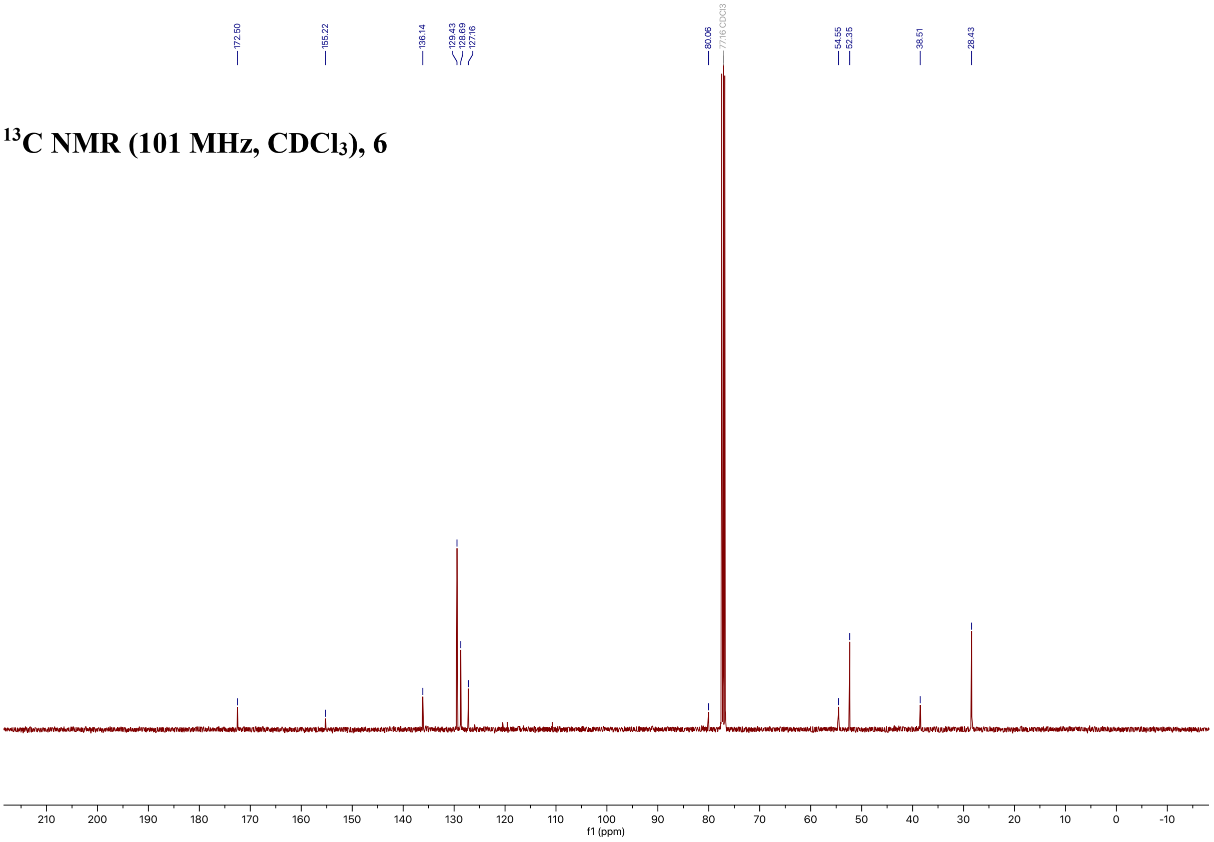

^{13}C NMR (101 MHz, $CDCl_3$), 6

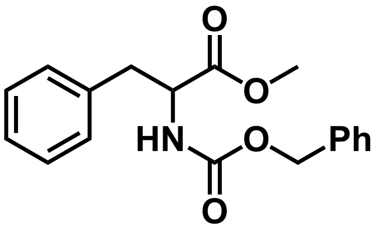

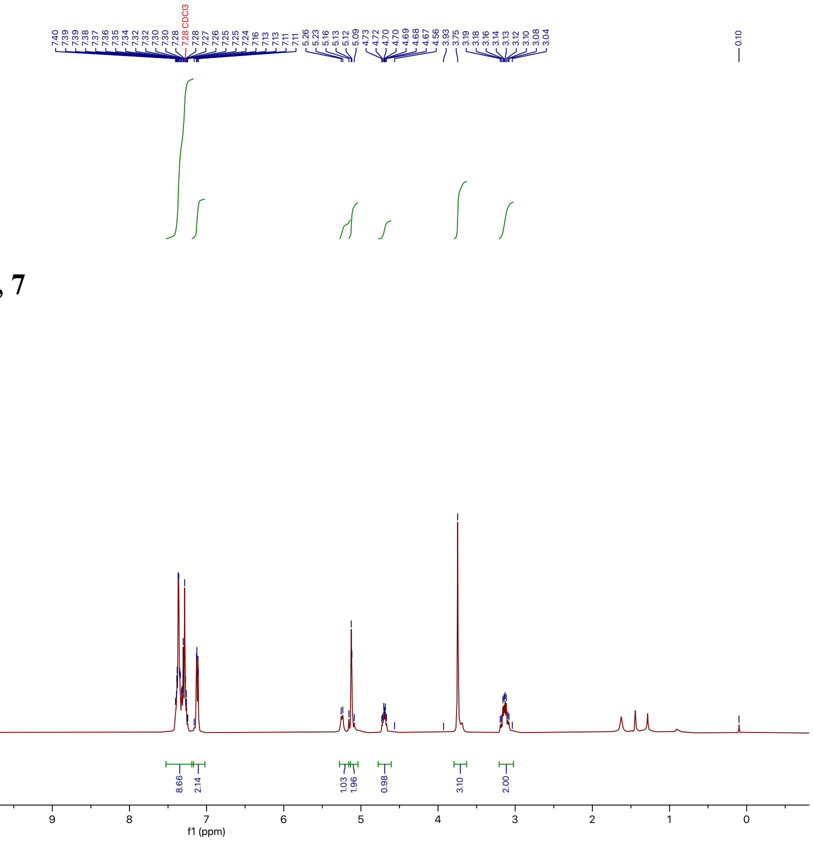

^{1}H NMR (400 MHz, CDCl$_3$), 7

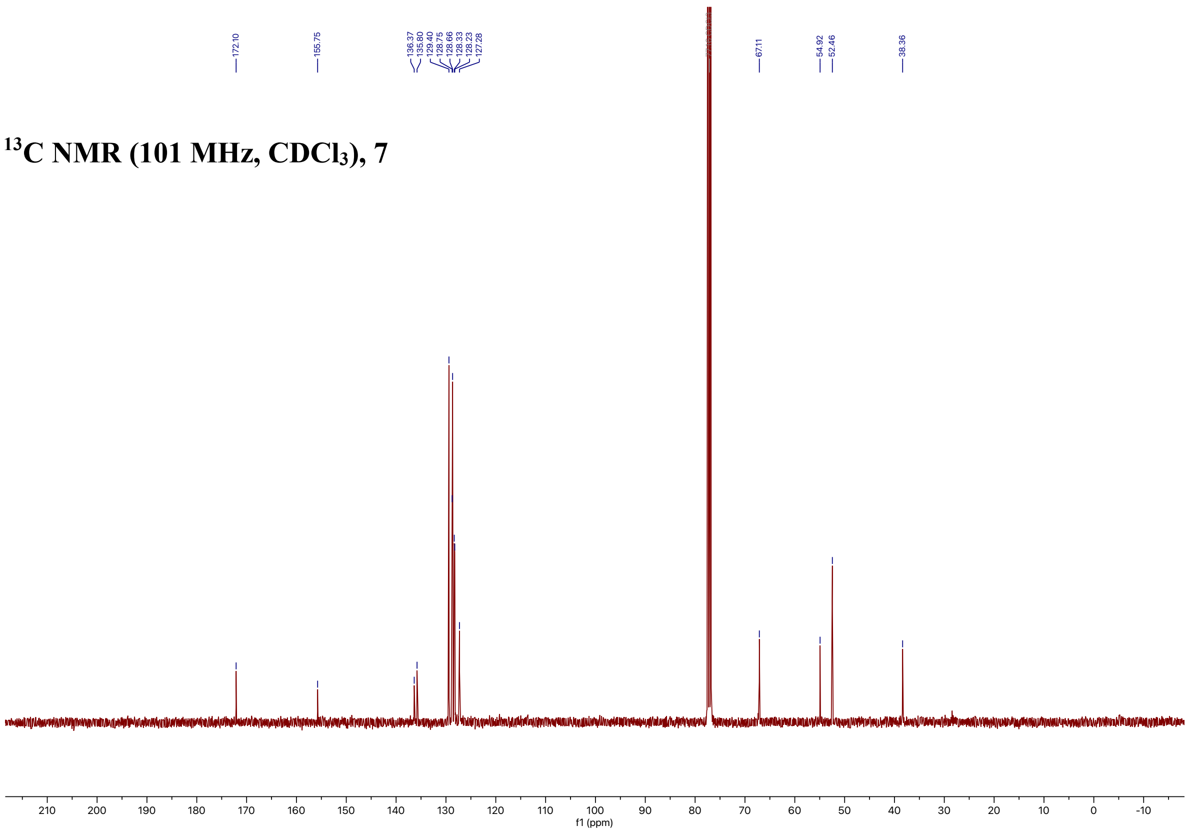

^{13}C NMR (101 MHz, CDCl$_3$), 7

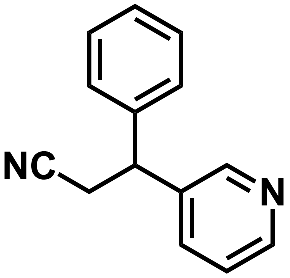

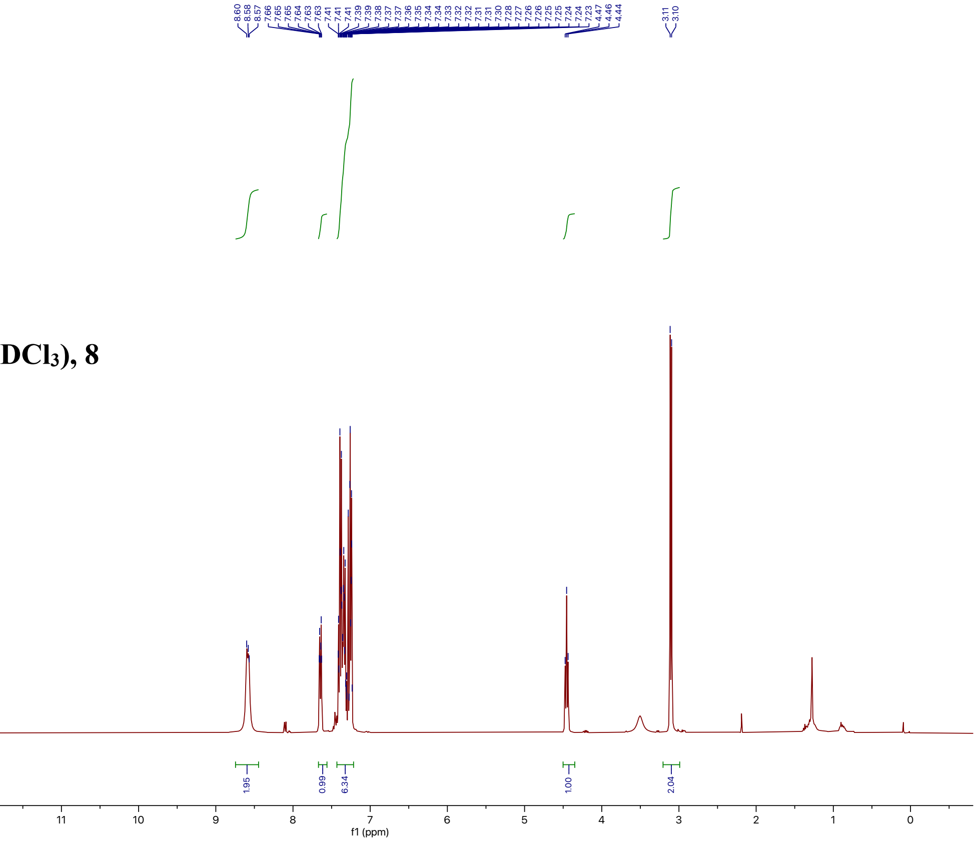

^{1}H NMR (400 MHz, CDCl$_3$), 8

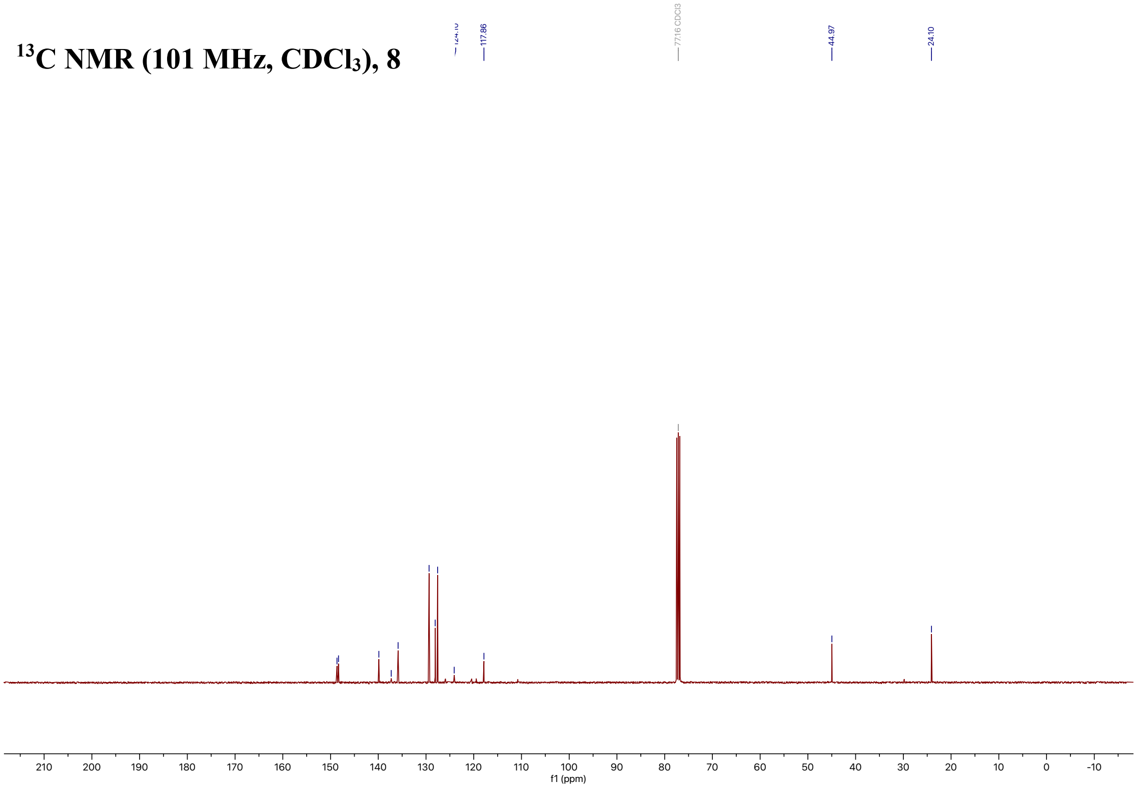

^{13}C NMR (101 MHz, CDCl$_3$), 8

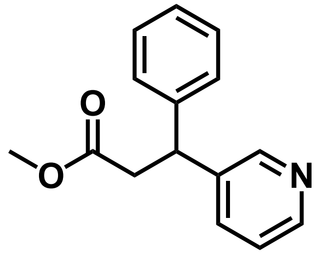

^{1}H NMR (400 MHz, CDCl$_3$), 9

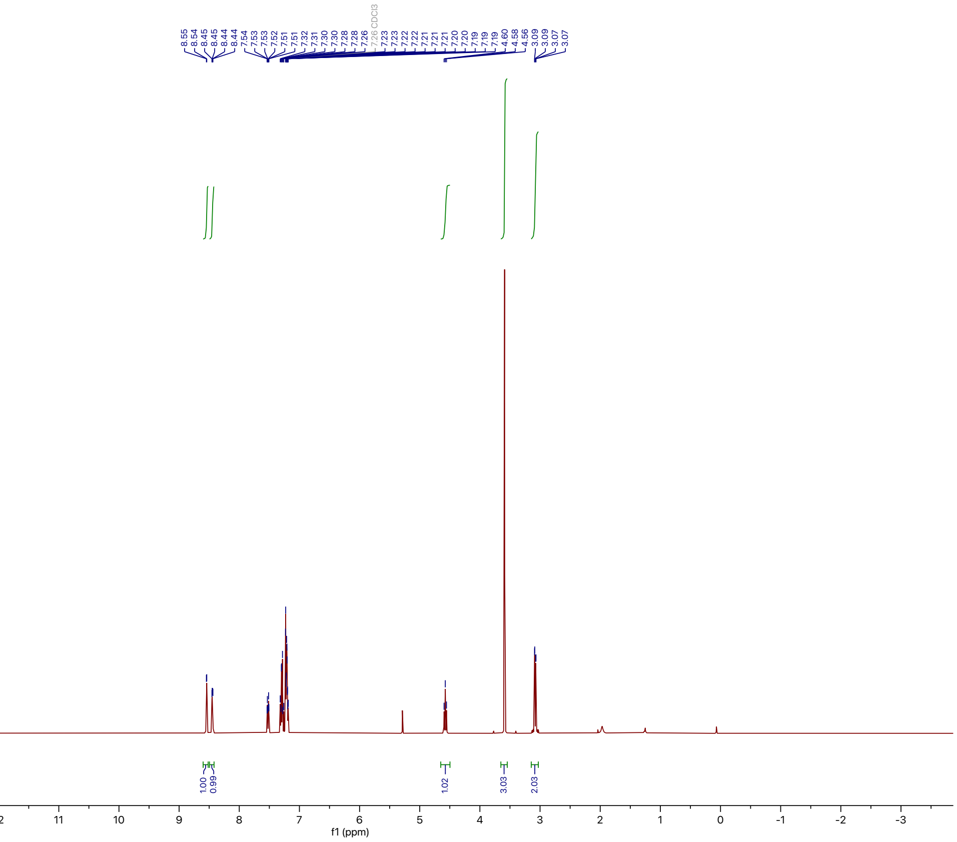

^{13}C NMR (101 MHz, CDCl$_3$), 9

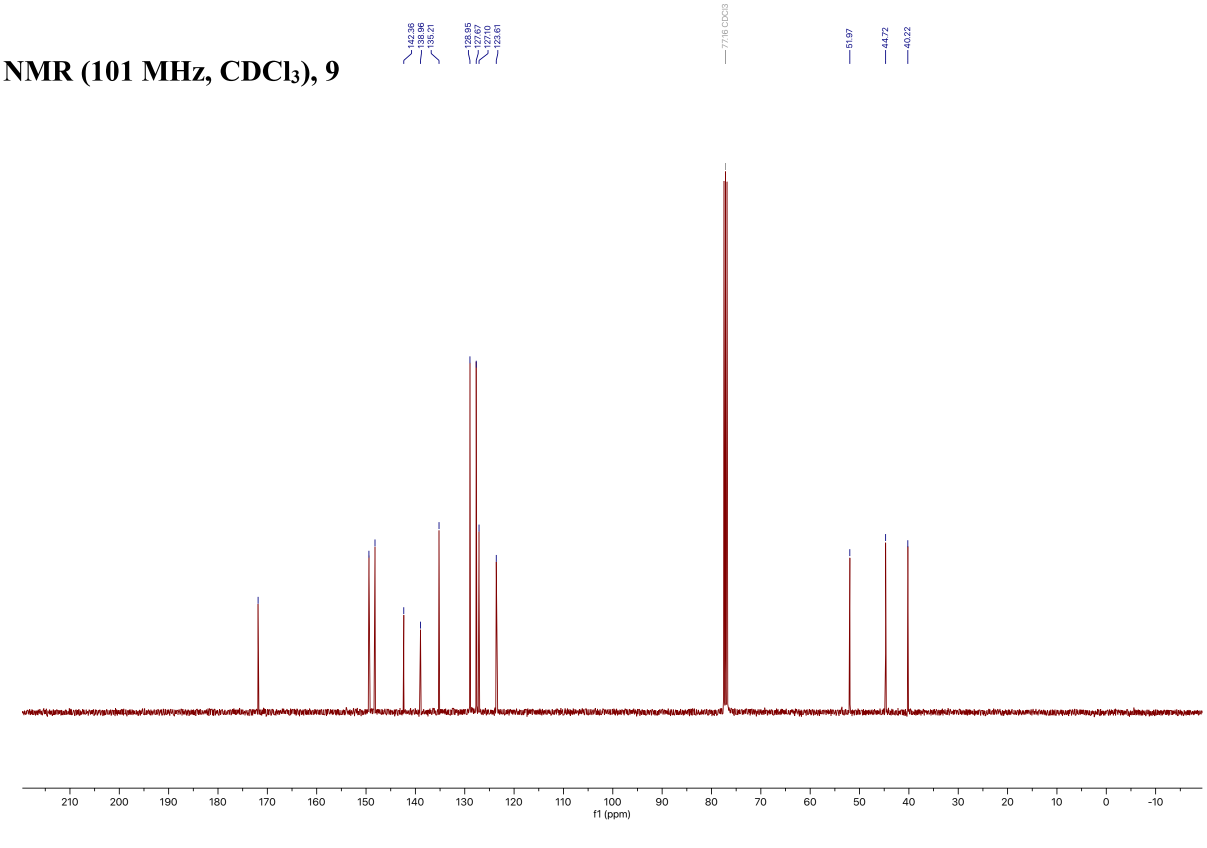

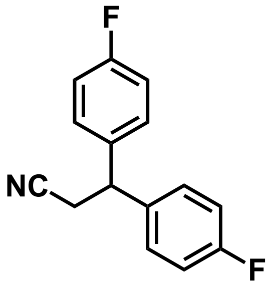

^{1}H NMR (400 MHz, $CDCl_3$), 10

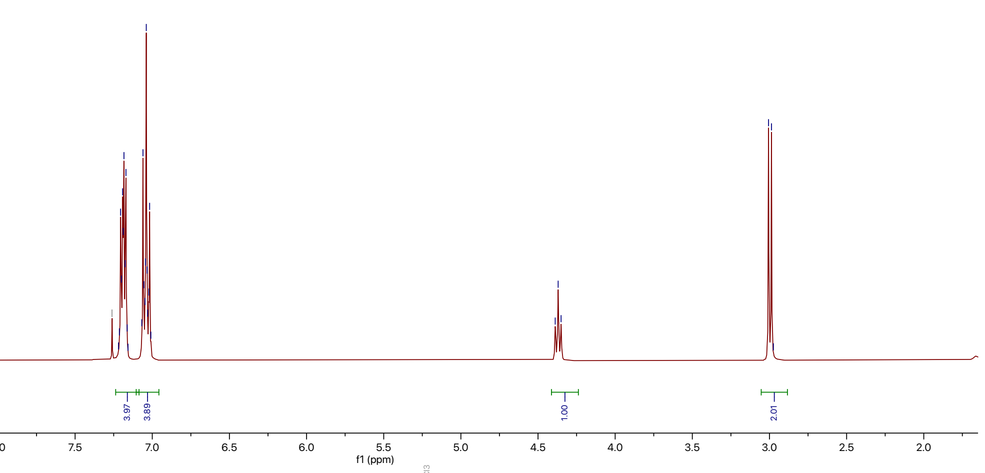

^{13}C NMR (101 MHz, $CDCl_3$), 10

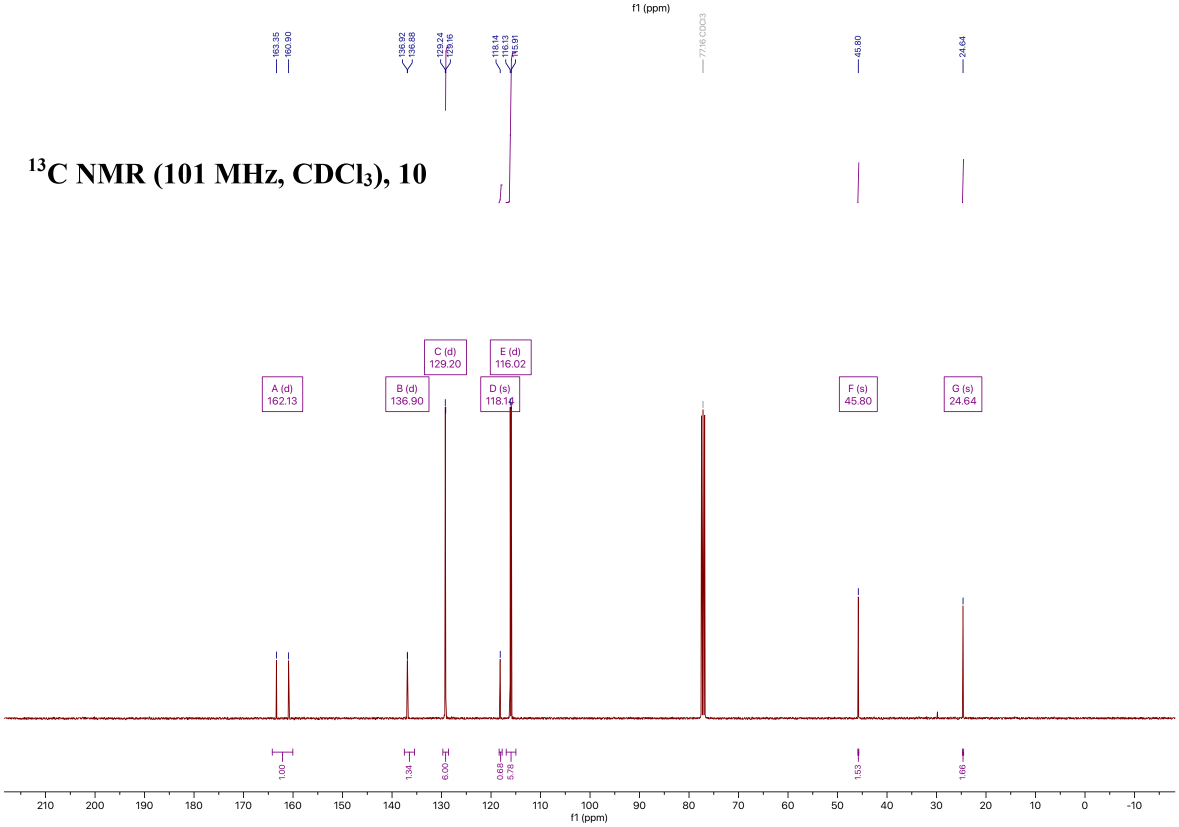

^{19}F NMR (376 MHz, $CDCl_3$), 10

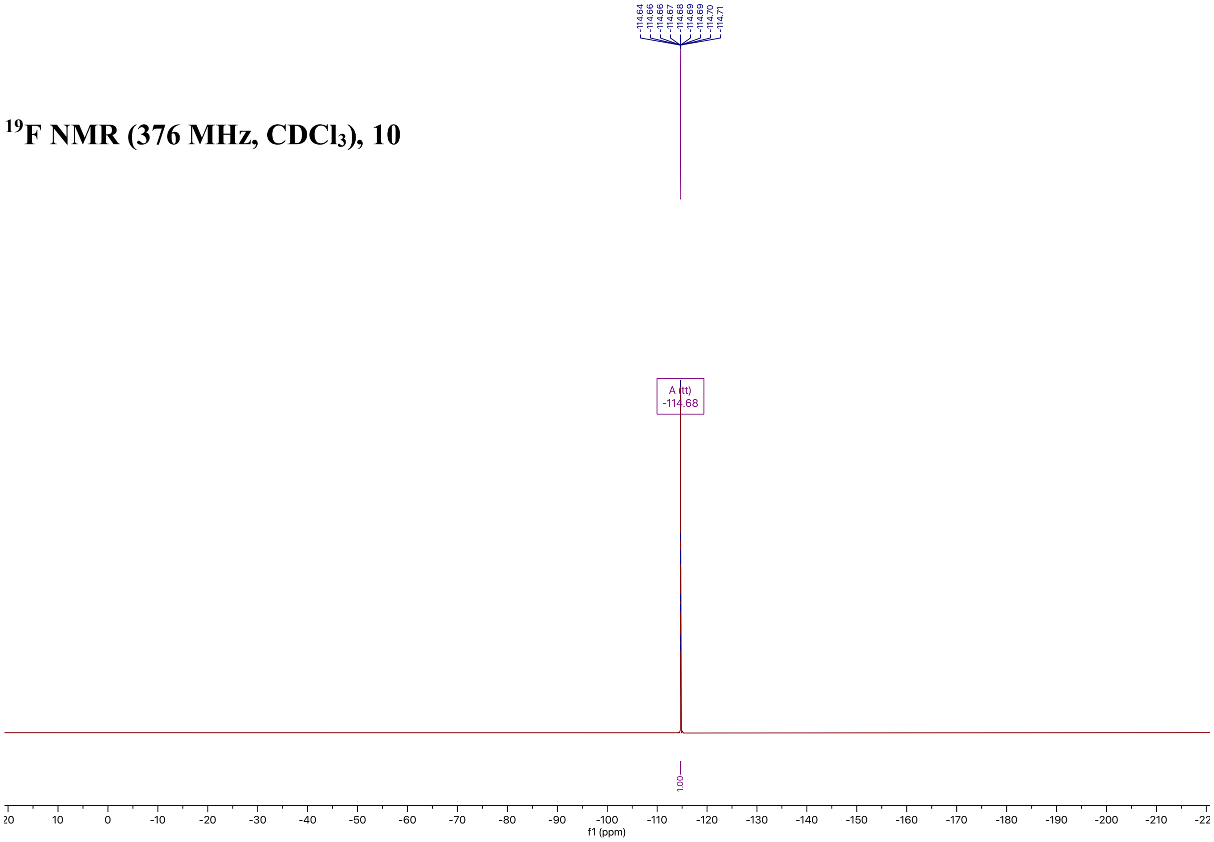

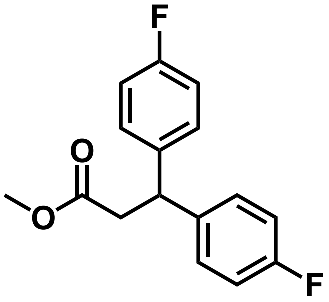

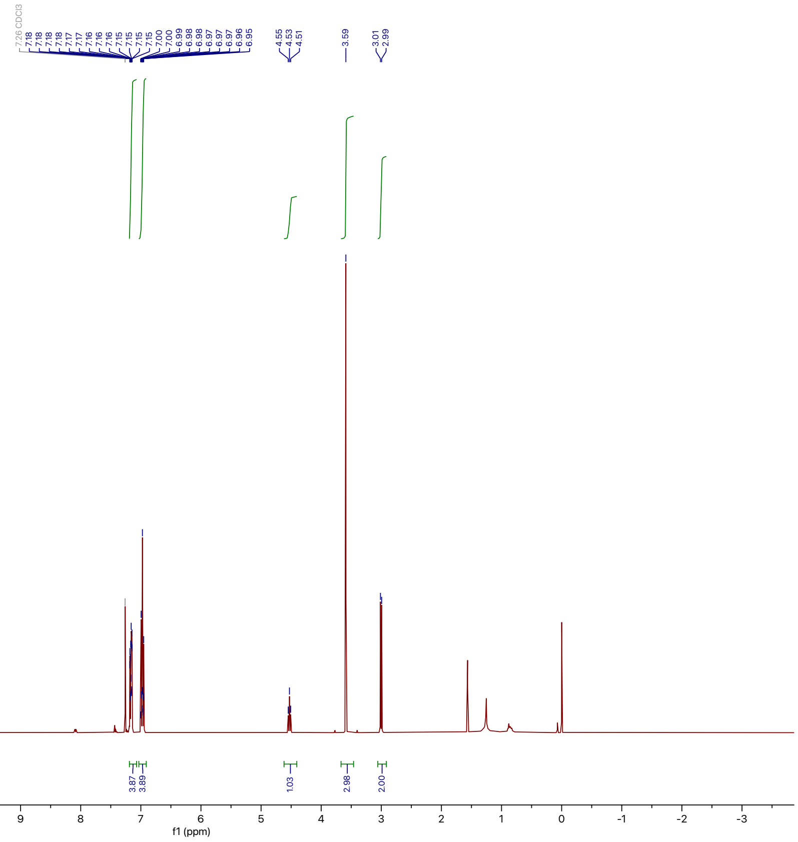

^{1}H NMR (400 MHz, $CDCl_3$), 11

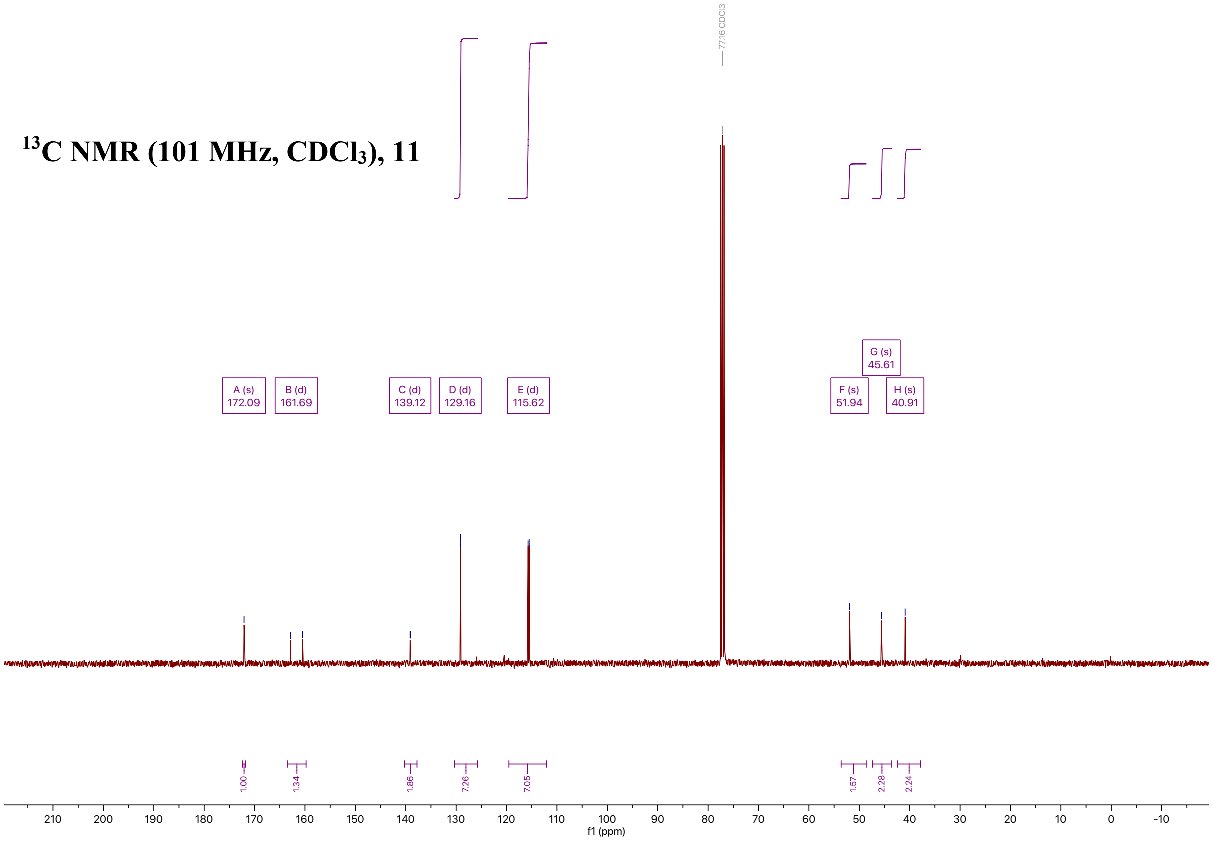

^{13}C NMR (101 MHz, $CDCl_3$), 11

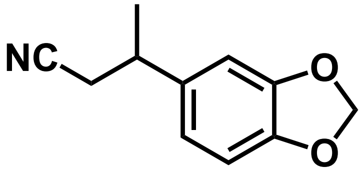

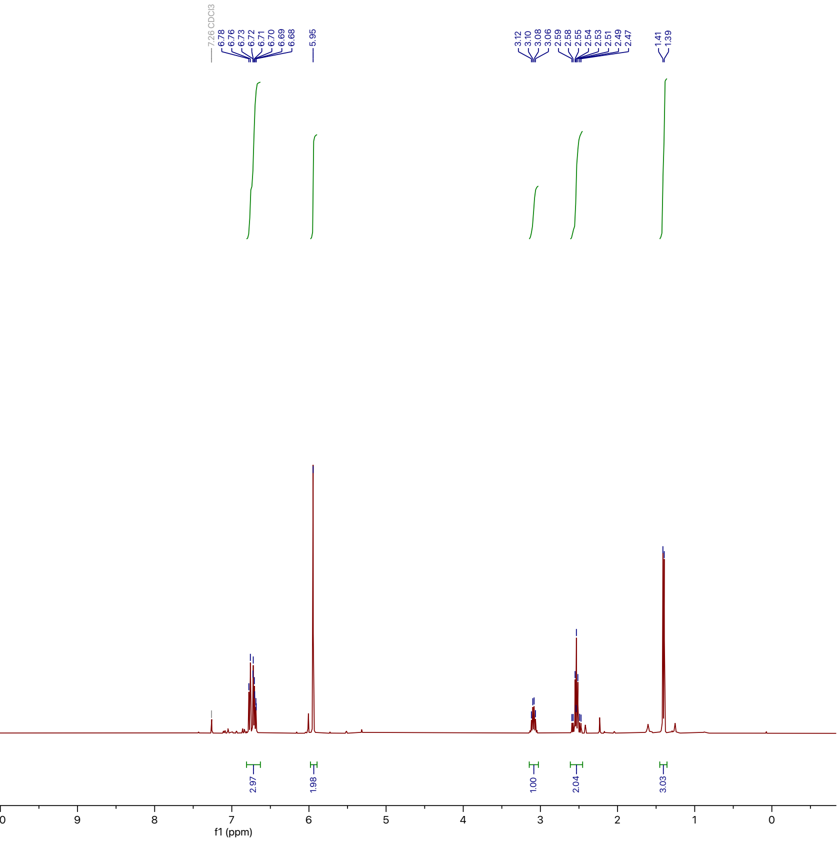

^{1}H NMR (400 MHz, CDCl$_3$),12

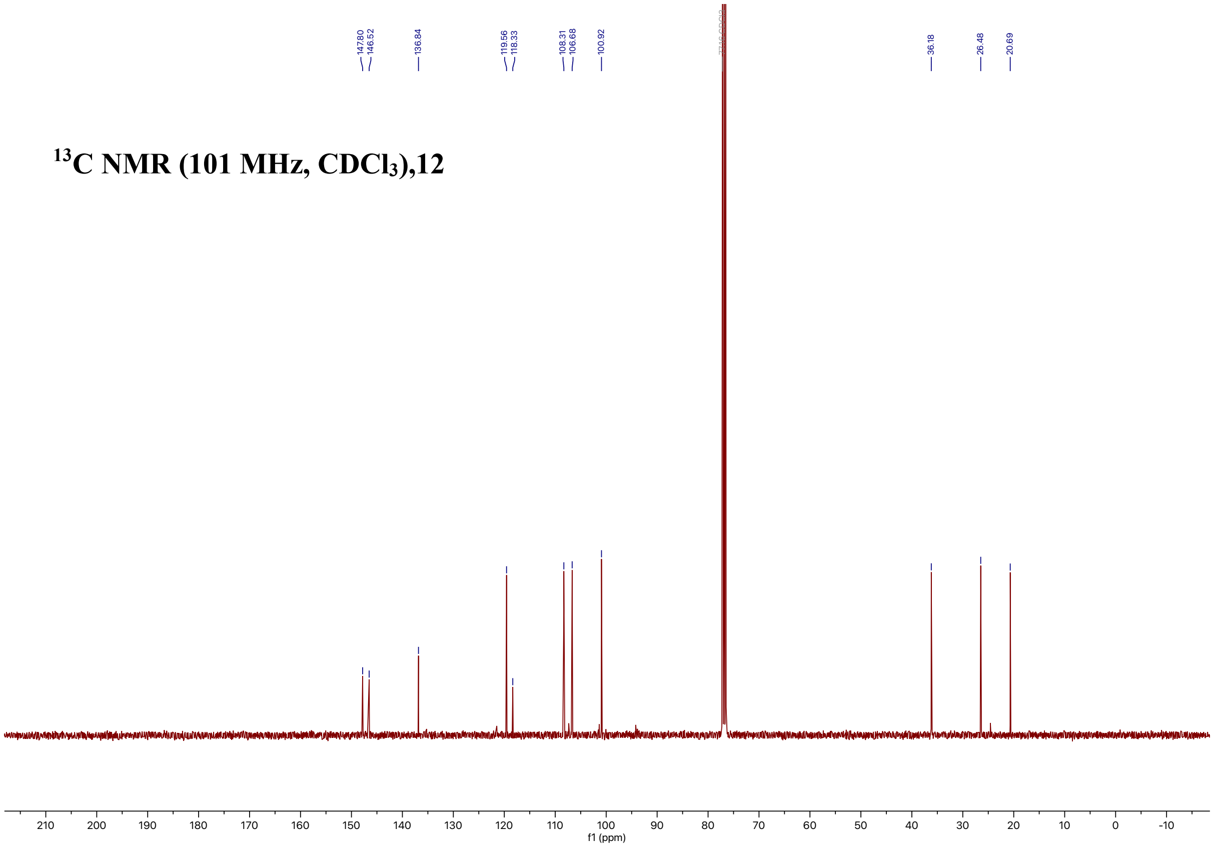

^{13}C NMR (101 MHz, CDCl$_3$),12

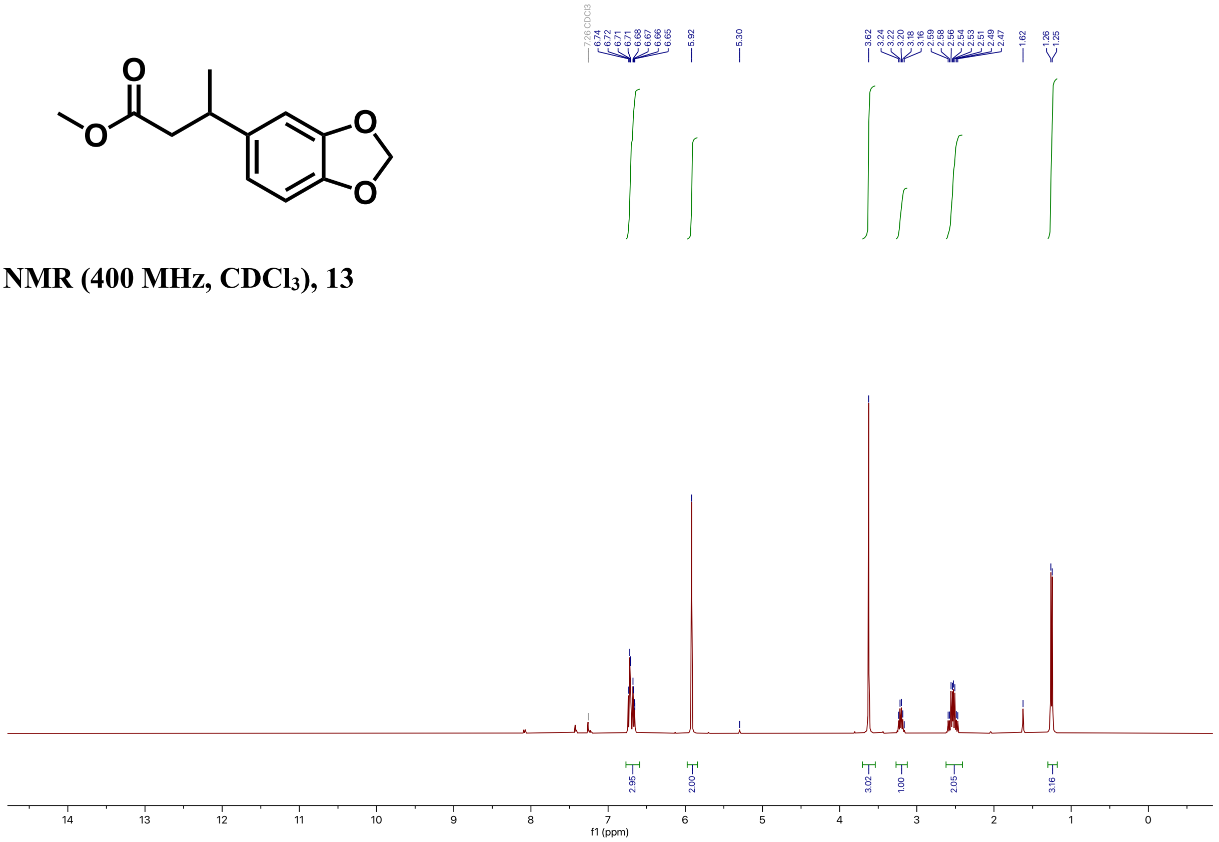

^{1}H NMR (400 MHz, CDCl$_3$), 13

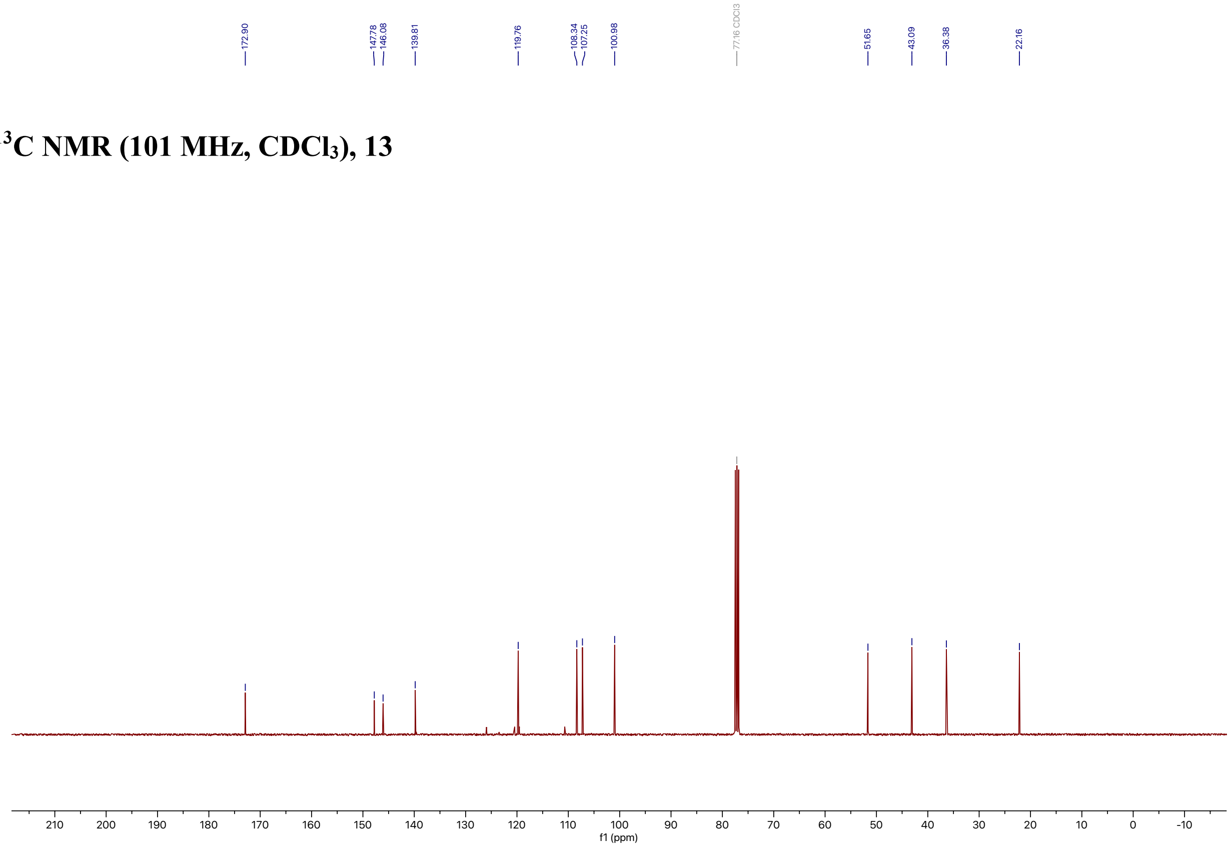

^{13}C NMR (101 MHz, CDCl$_3$), 13

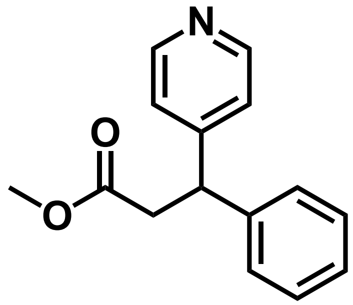

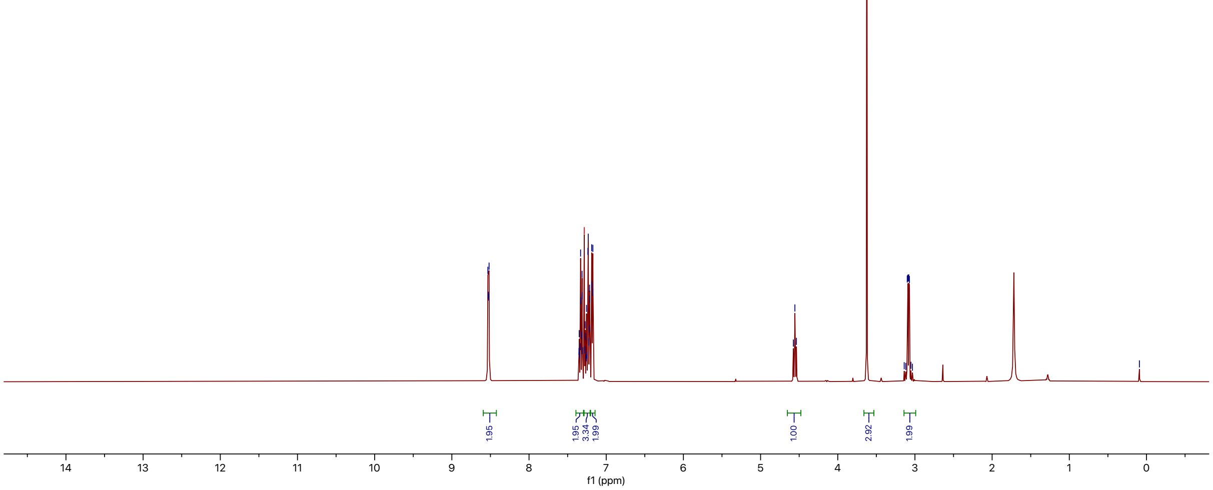

^{1}H NMR (400 MHz, $CDCl_3$),14

^{13}C NMR (101 MHz, $CDCl_3$),14

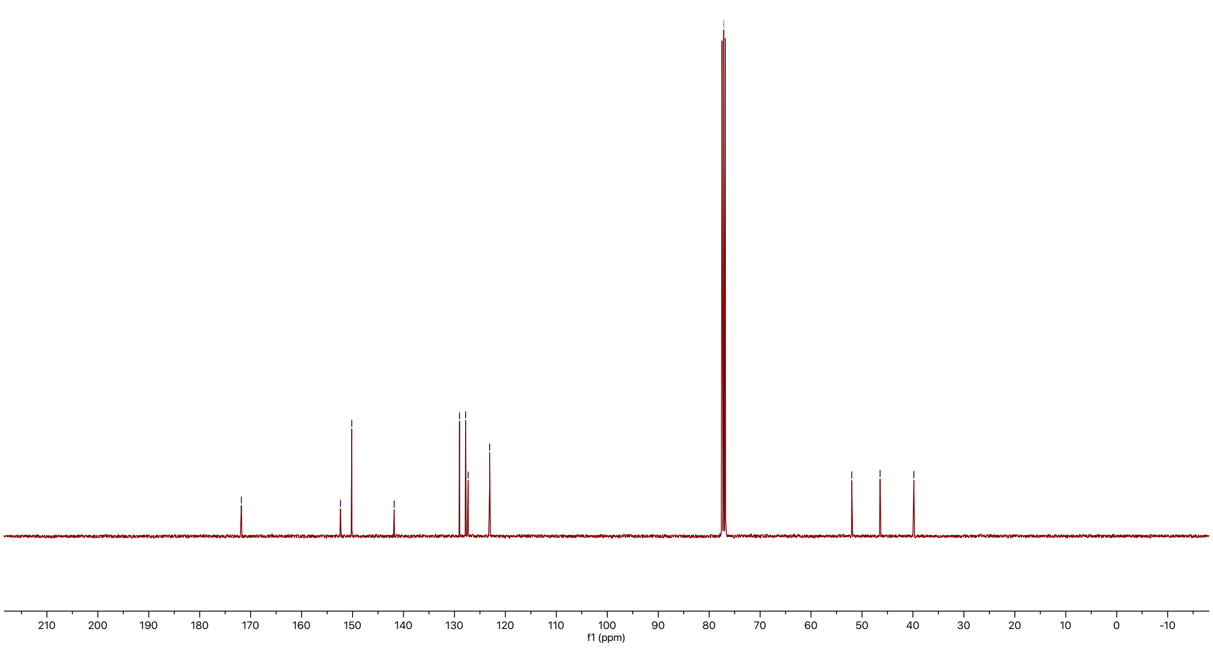

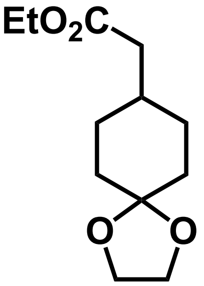

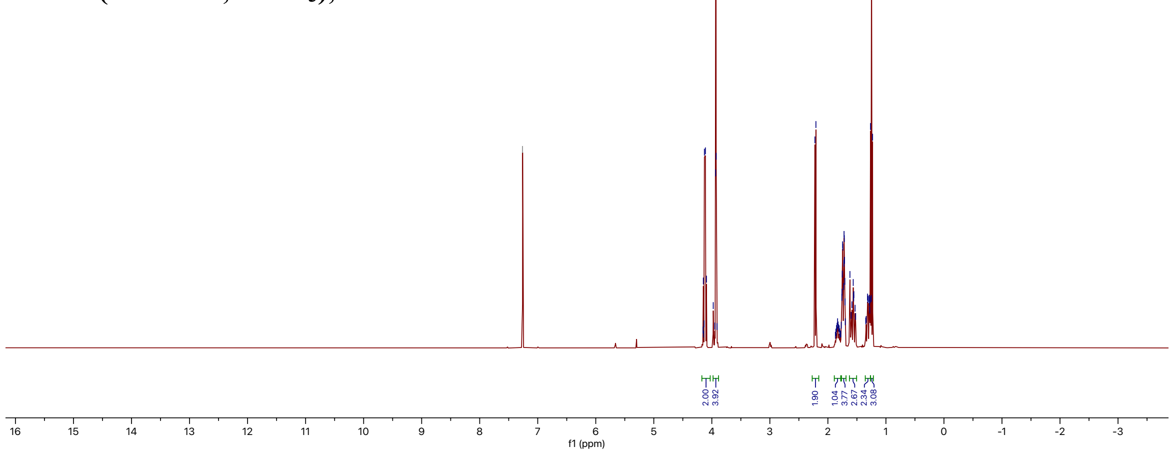

^{1}H NMR (400 MHz, $CDCl_3$), 15

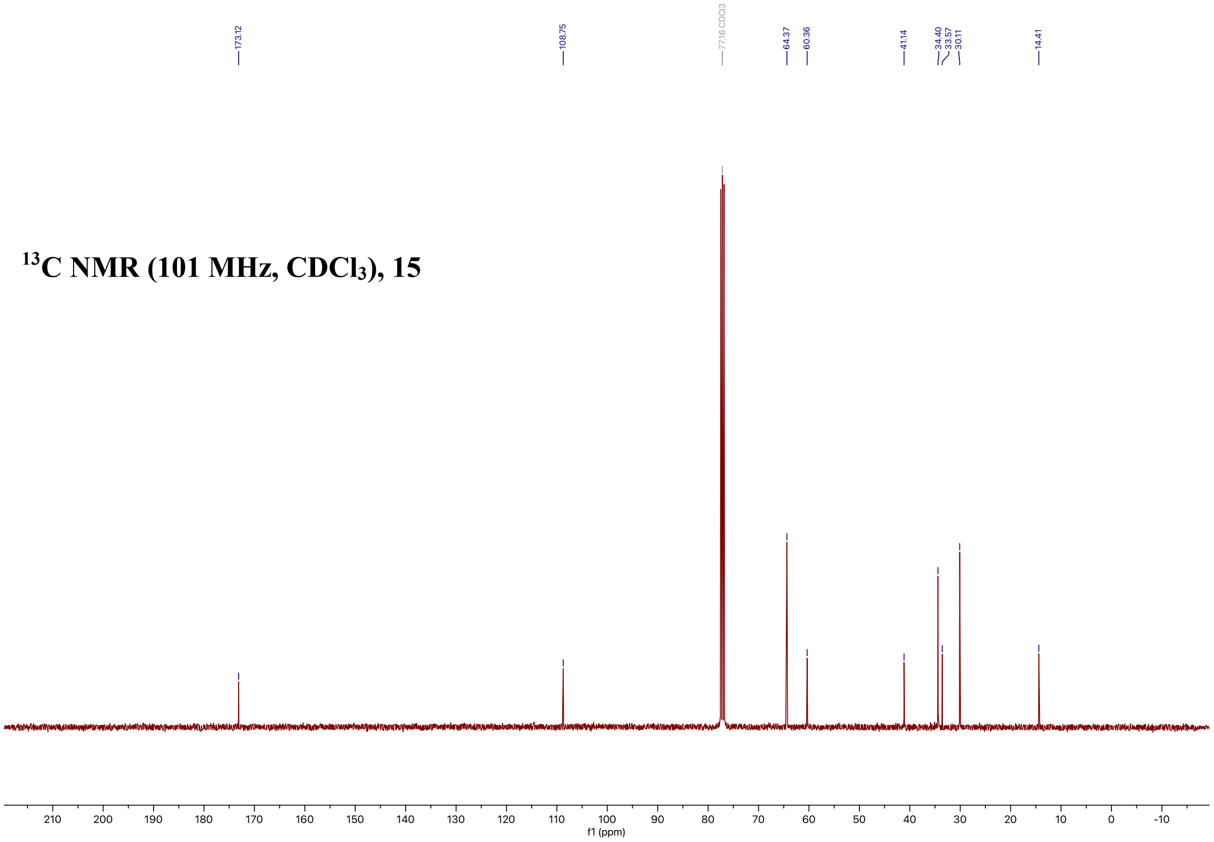

^{13}C NMR (101 MHz, $CDCl_3$), 15

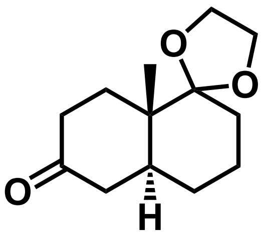

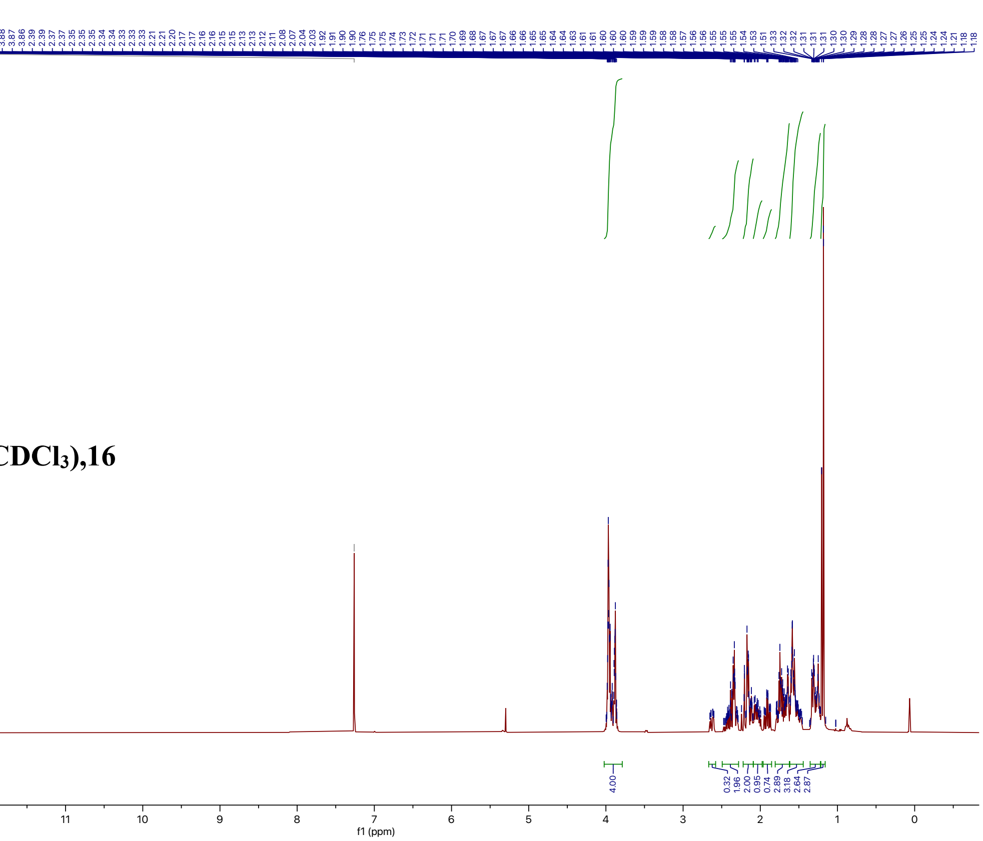

^{1}H NMR (400 MHz, CDCl$_3$),16

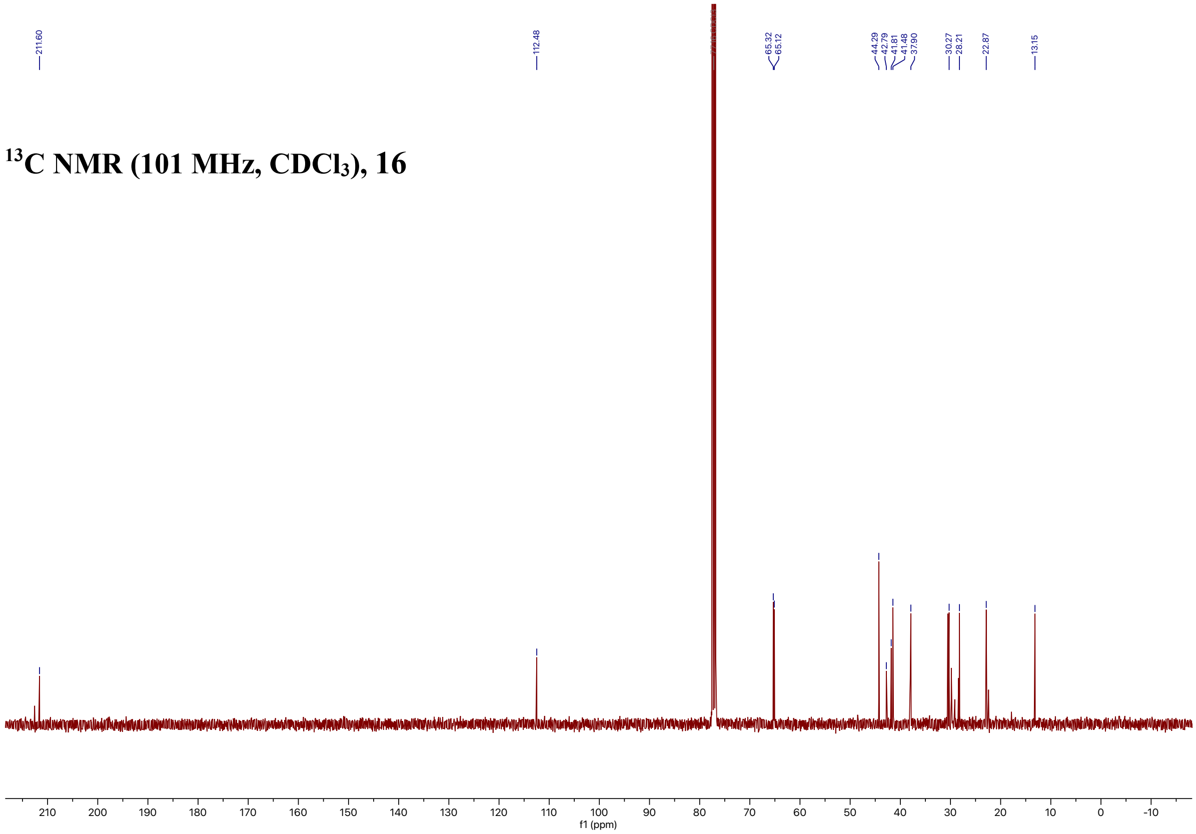

^{13}C NMR (101 MHz, CDCl$_3$), 16

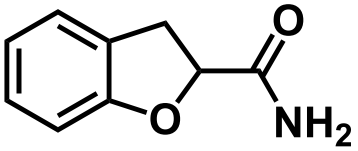

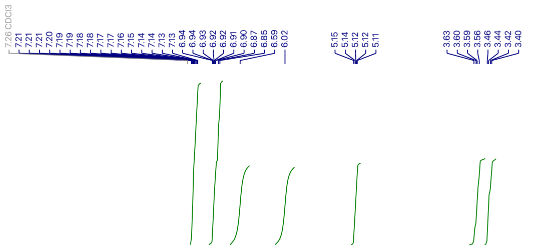

^{1}H NMR (400 MHz, CDCl$_3$), 17

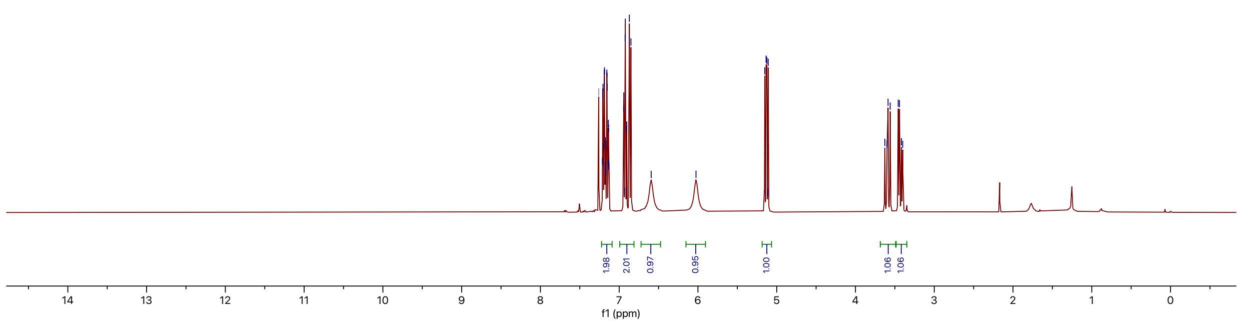

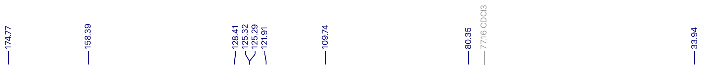

^{13}C NMR (151 MHz, CDCl$_3$), 17

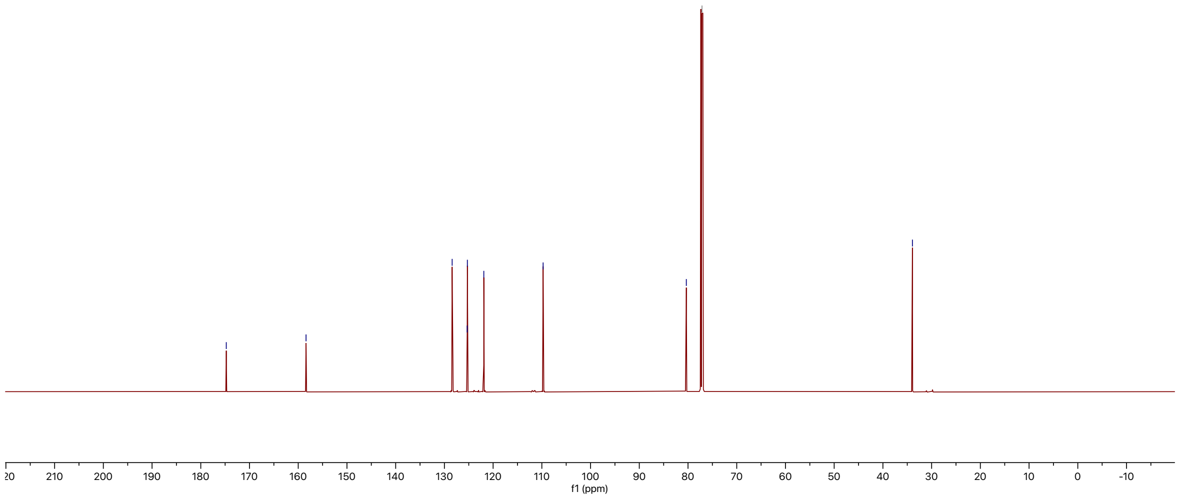

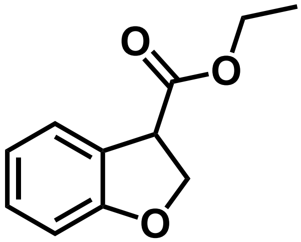

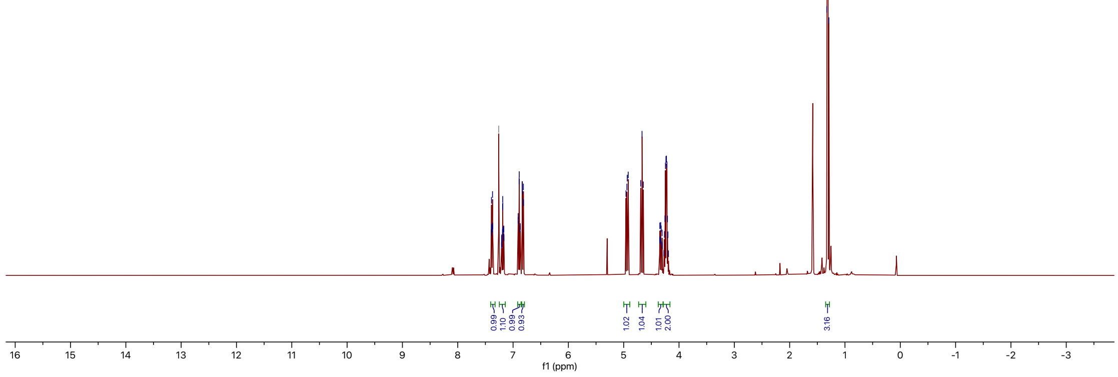

^{1}H NMR (400 MHz, $CDCl_3$), 18

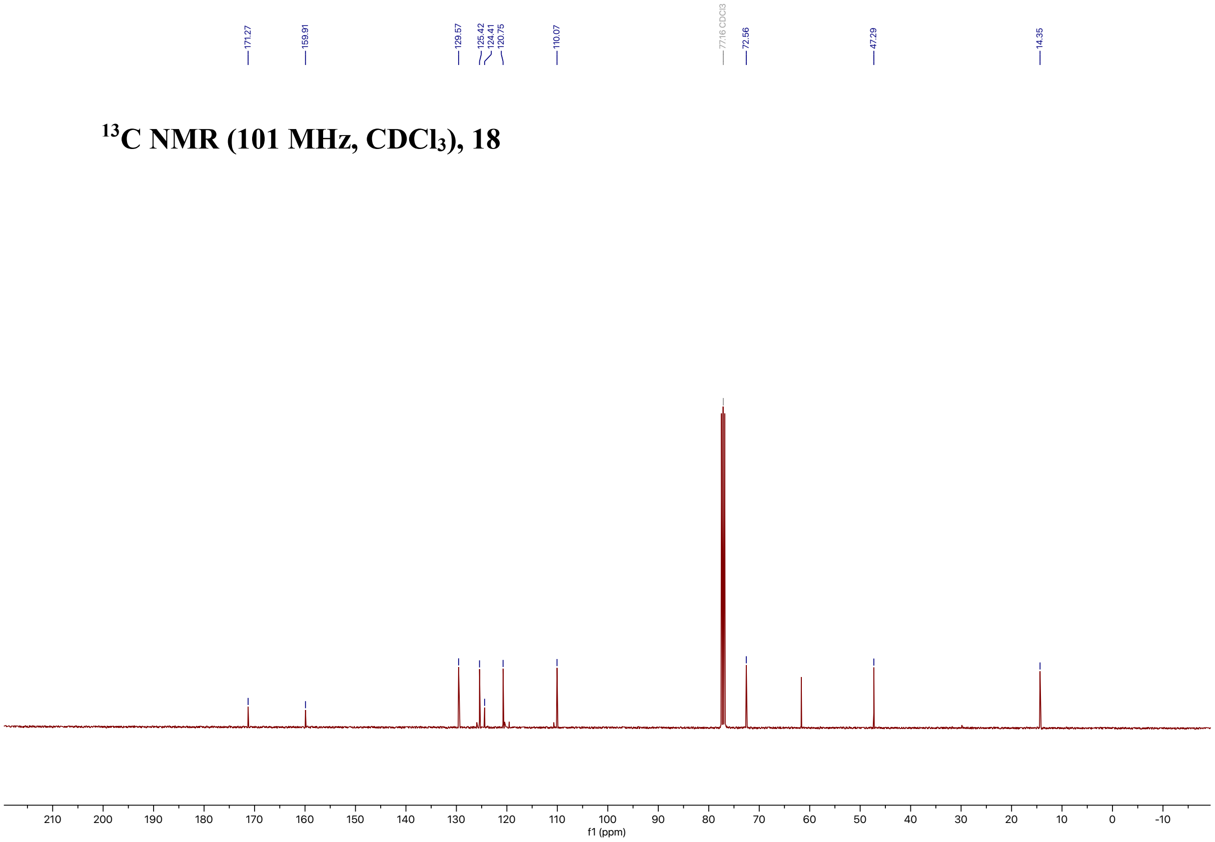

^{13}C NMR (101 MHz, $CDCl_3$), 18

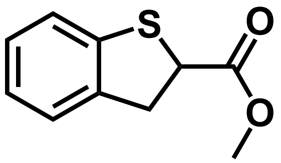

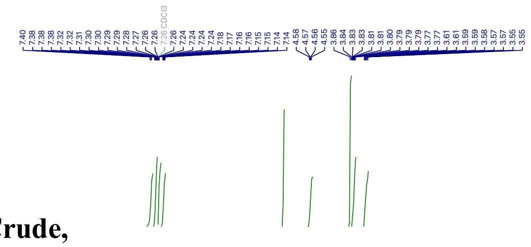

^{1}H NMR (400 MHz, $CDCl_3$), 19, Crude, NMR Yield

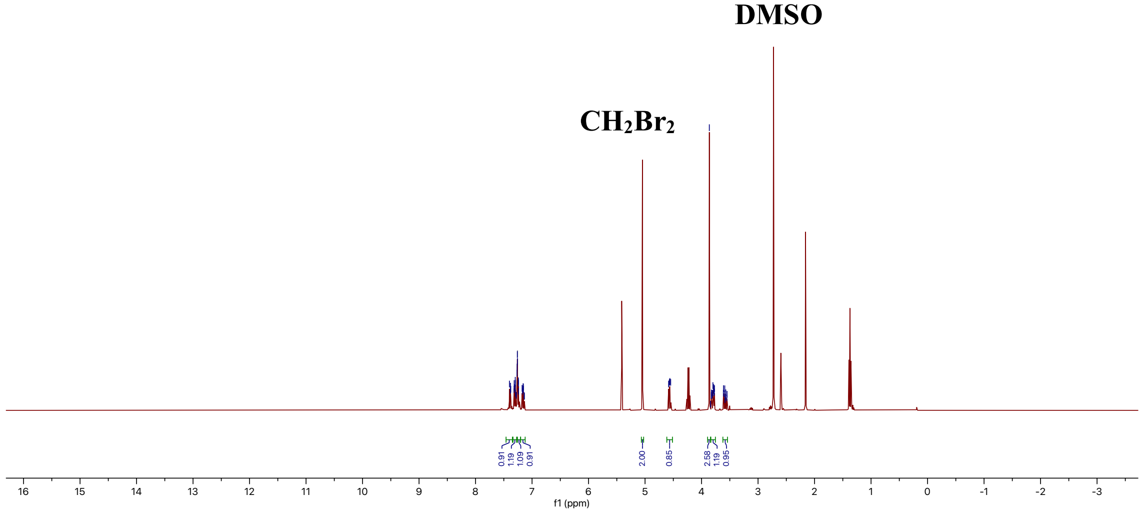

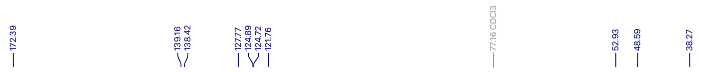

^{13}C NMR (101 MHz, $CDCl_3$), 19

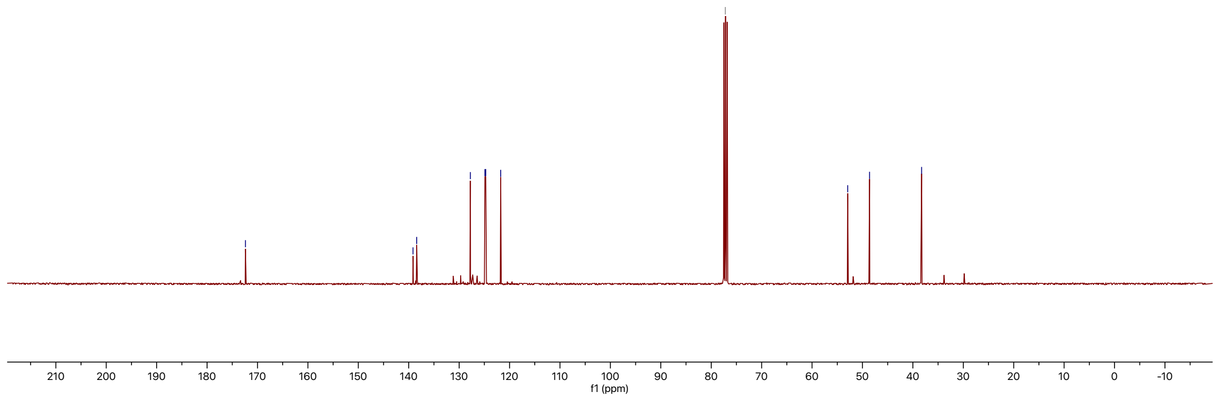

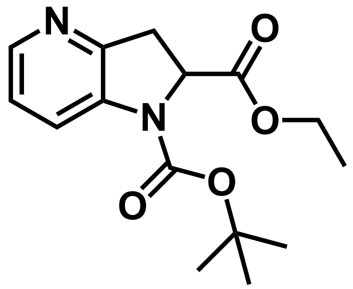

^{1}H NMR (400 MHz, DMSO), 20

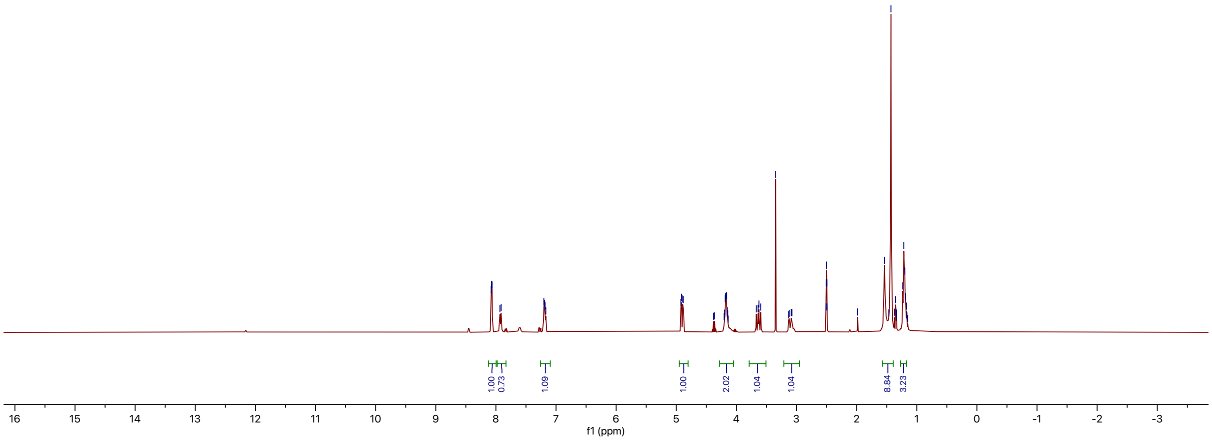

^{13}C NMR (101 MHz, DMSO), 20

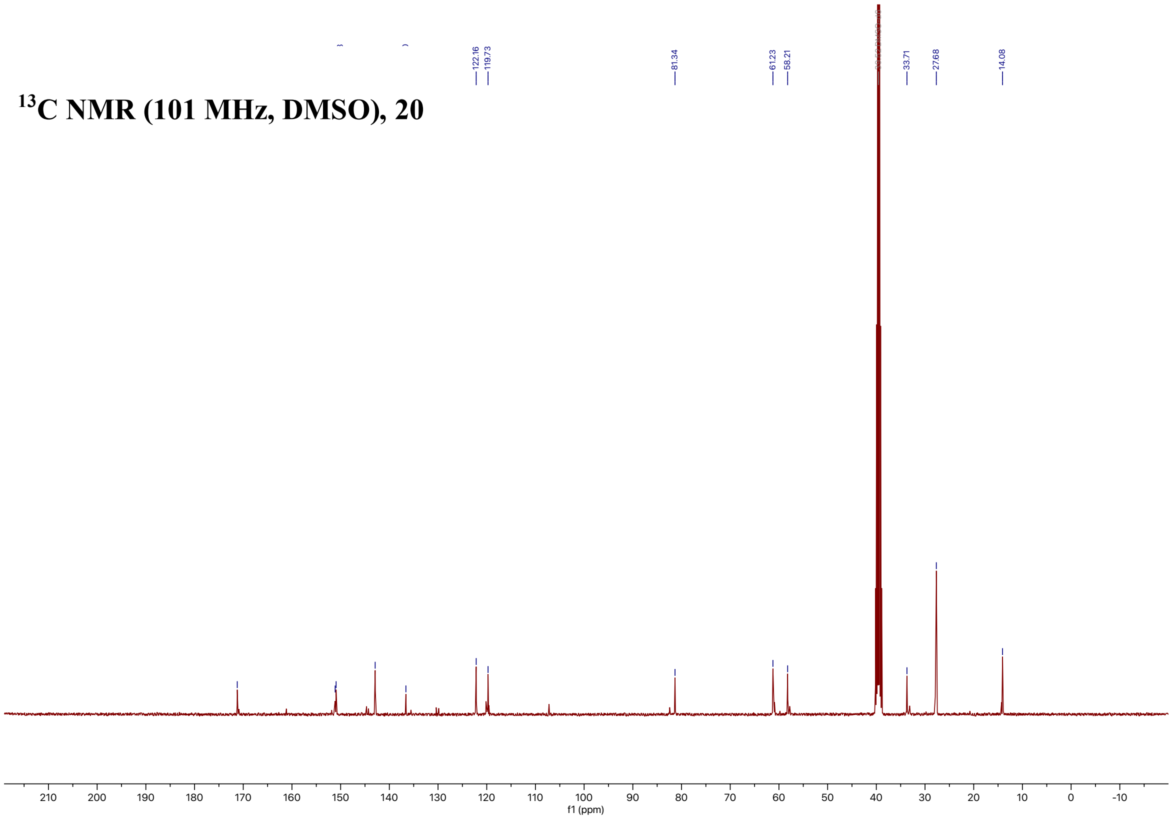

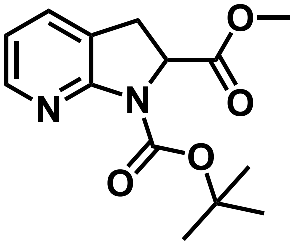

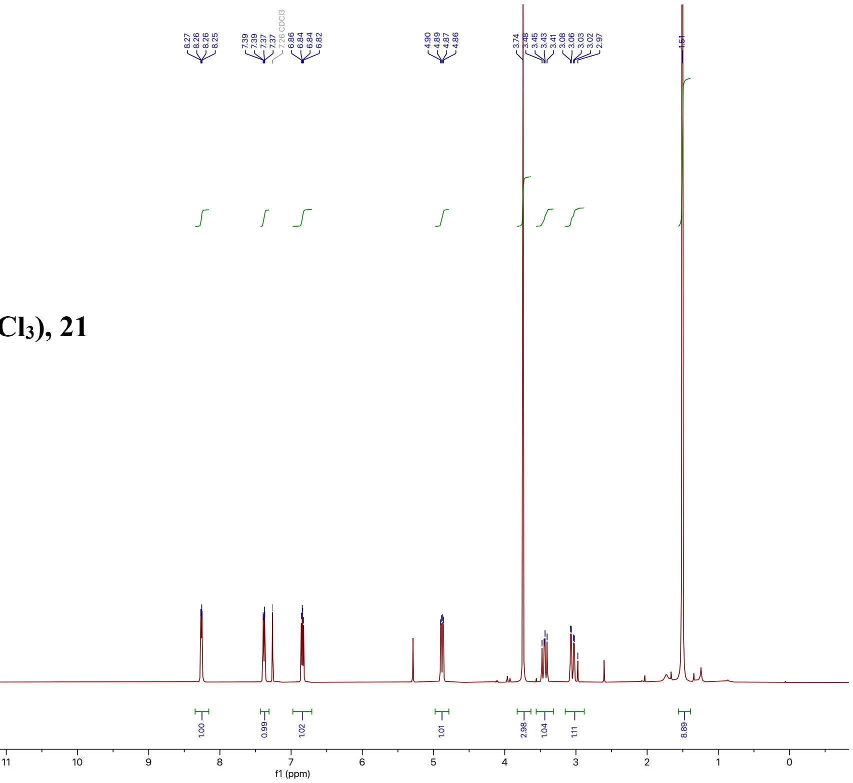

^{1}H NMR (400 MHz, CDCl$_3$), 21

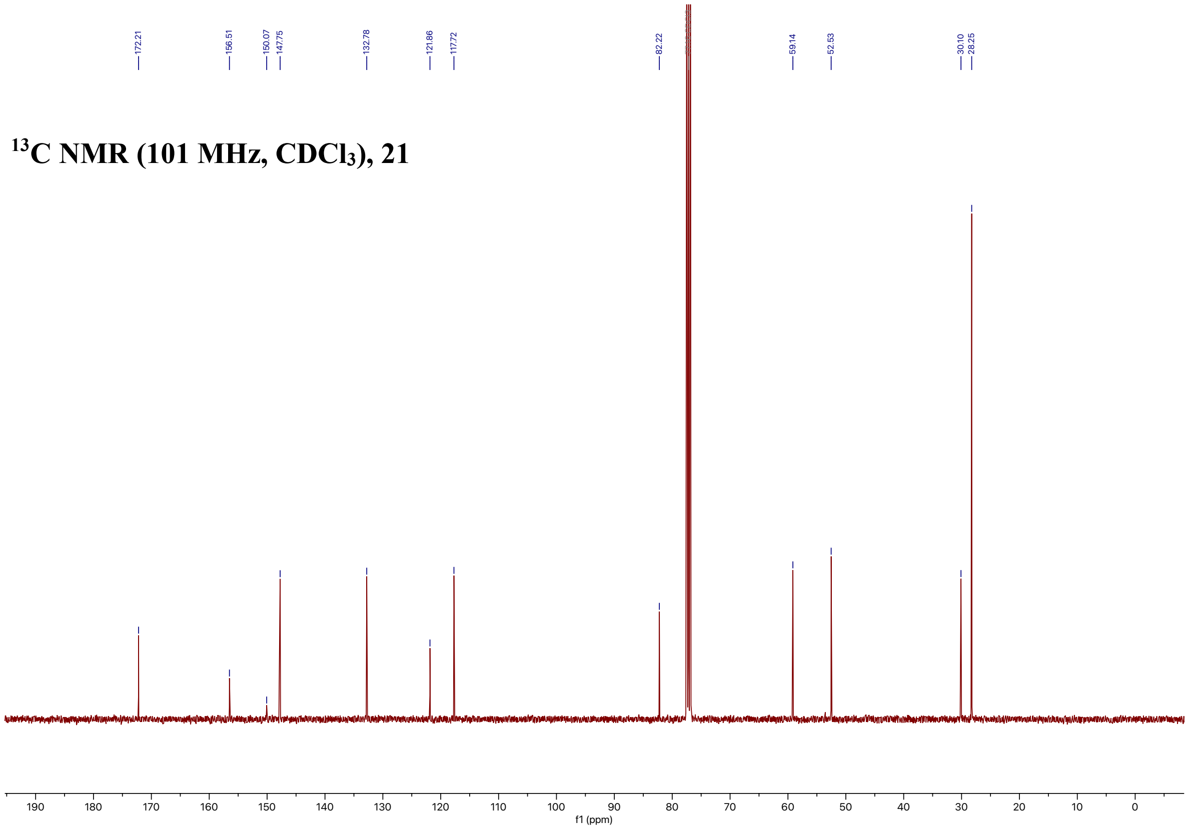

^{13}C NMR (101 MHz, CDCl$_3$), 21

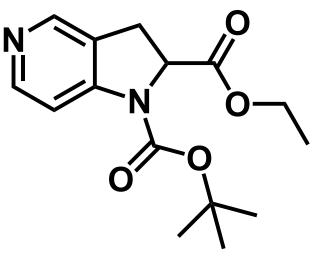

^{1}H NMR (400 MHz, DMSO), 22

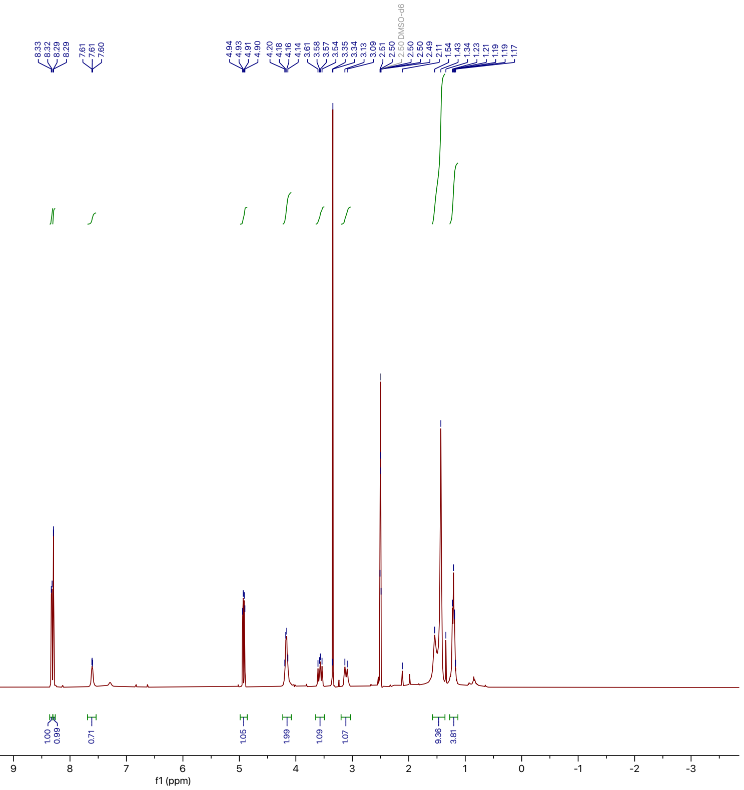

^{13}C NMR (101 MHz, DMSO), 22

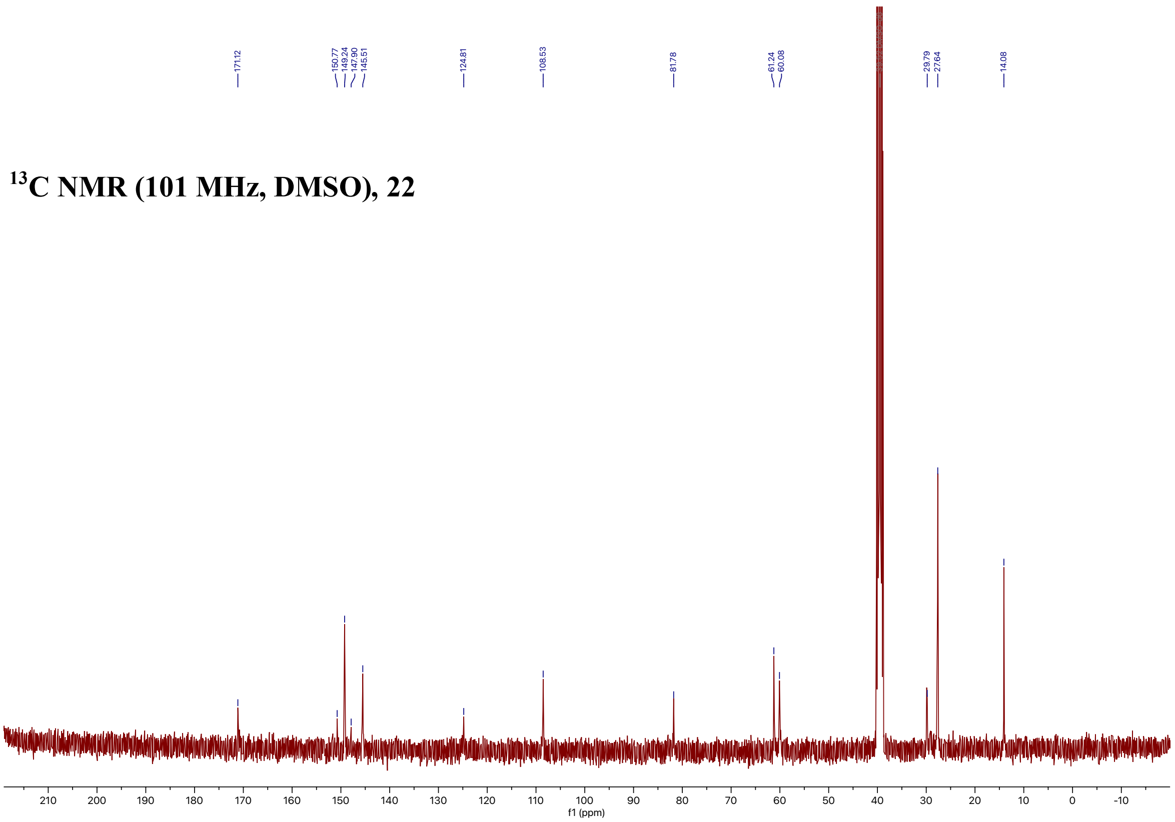

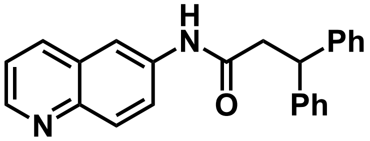

^{1}H NMR (400 MHz, $CDCl_3$), 23

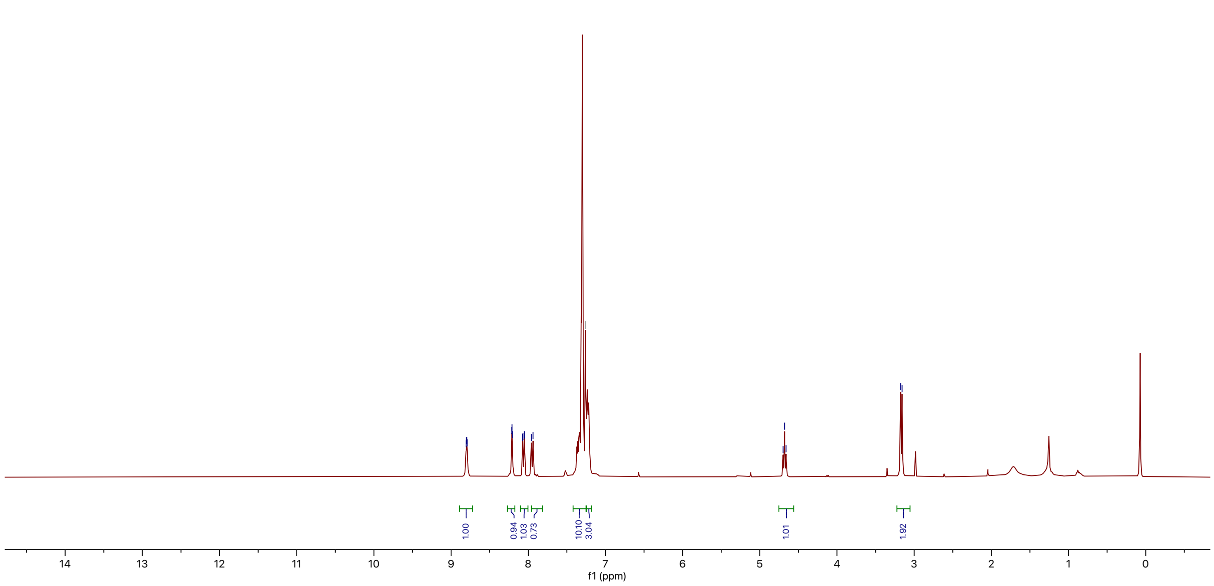

^{13}C NMR (101 MHz, $CDCl_3$), 23

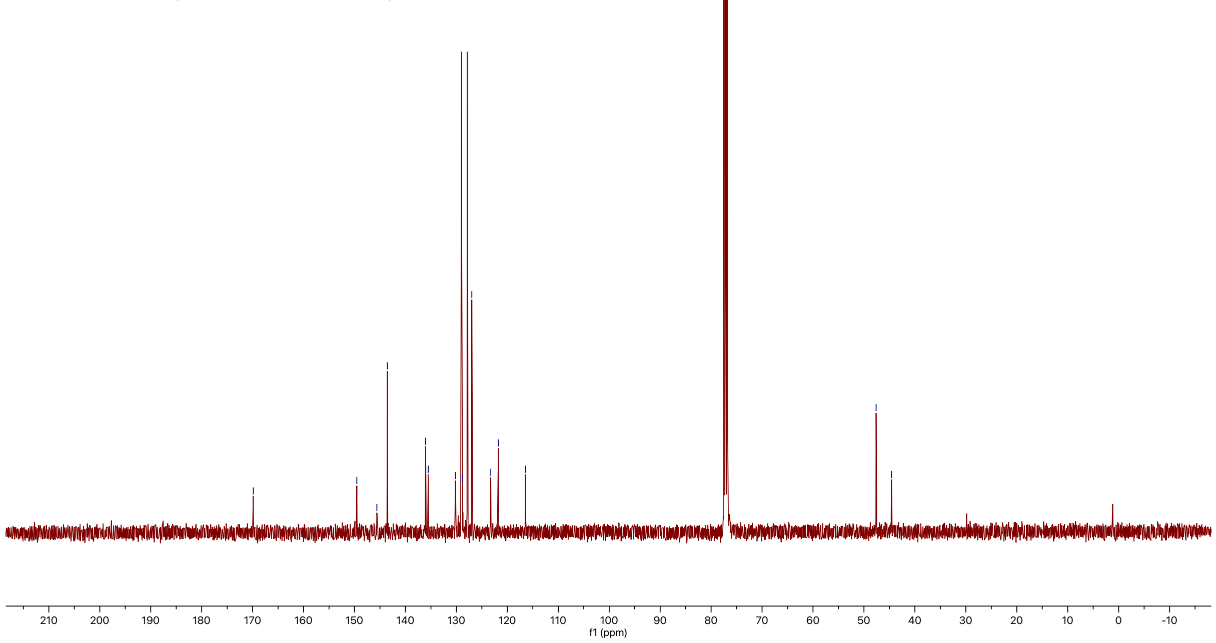

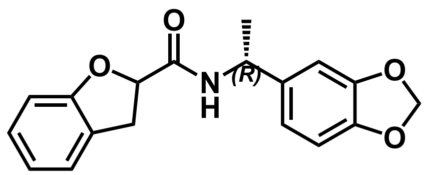

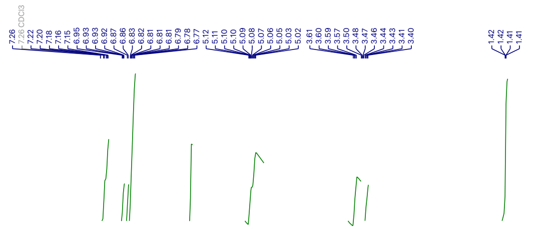

^{1}H NMR (600 MHz, CDCl$_3$), 24

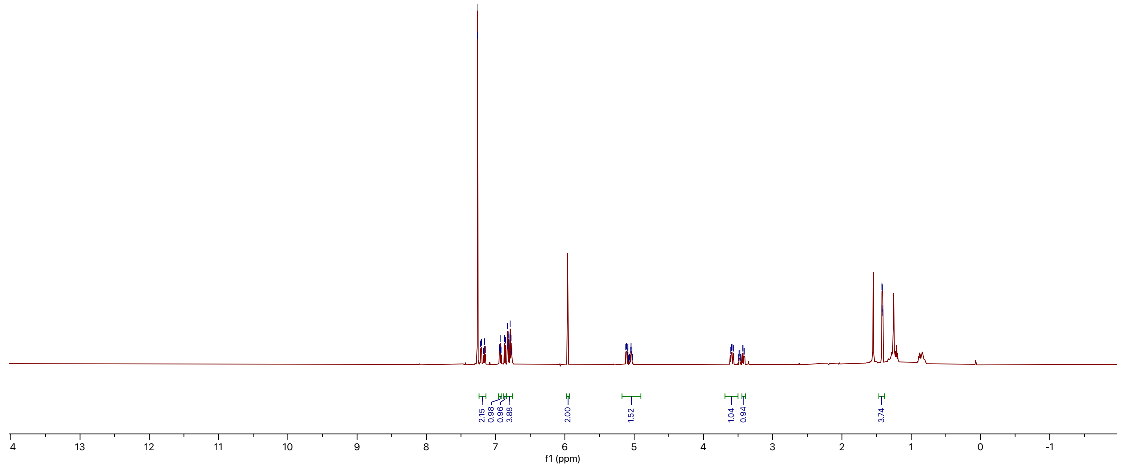

^{13}C NMR (101 MHz, CDCl$_3$), 24

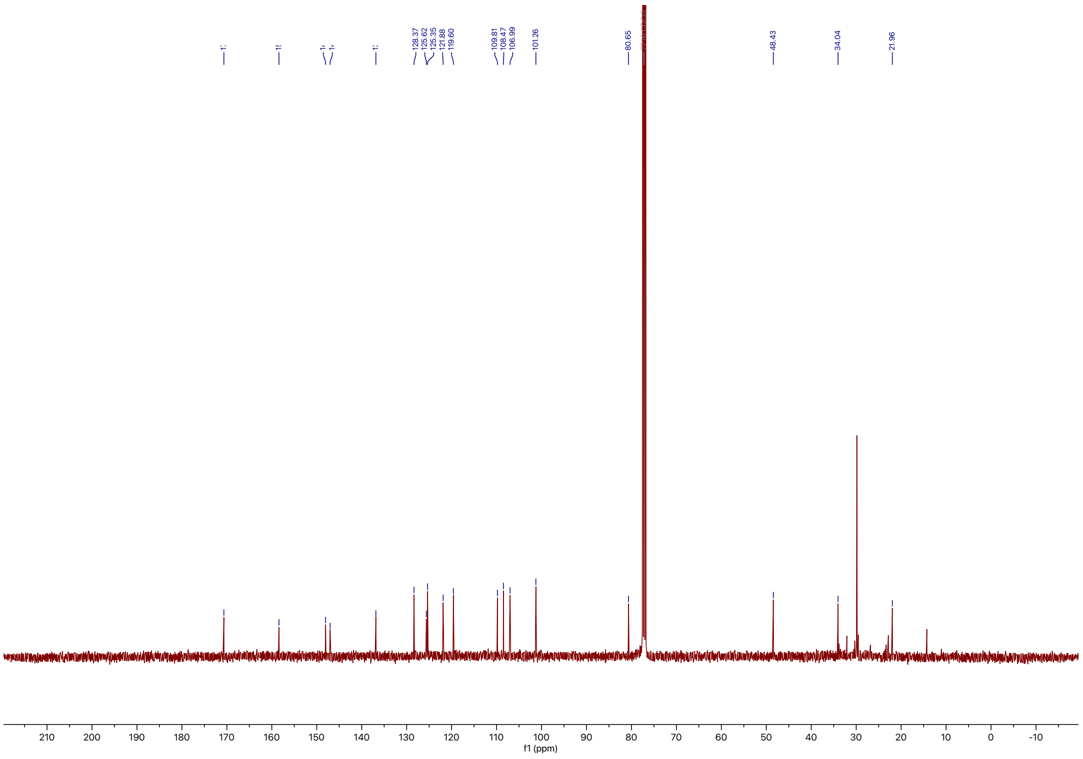

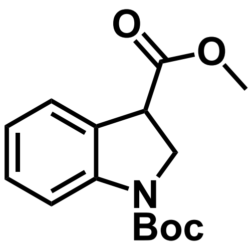

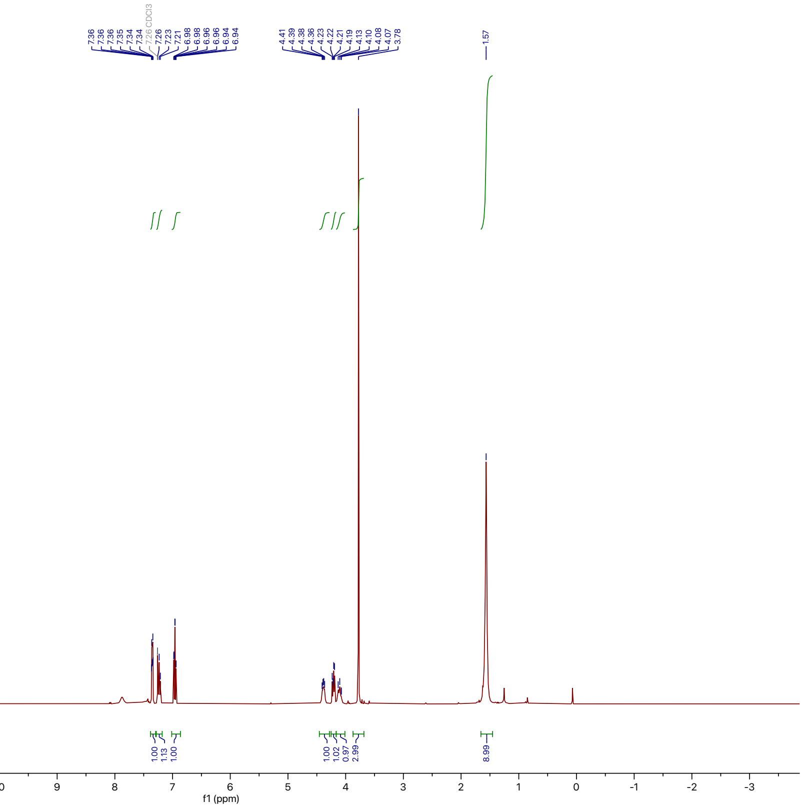

^{1}H NMR (400 MHz, $CDCl_3$), 25

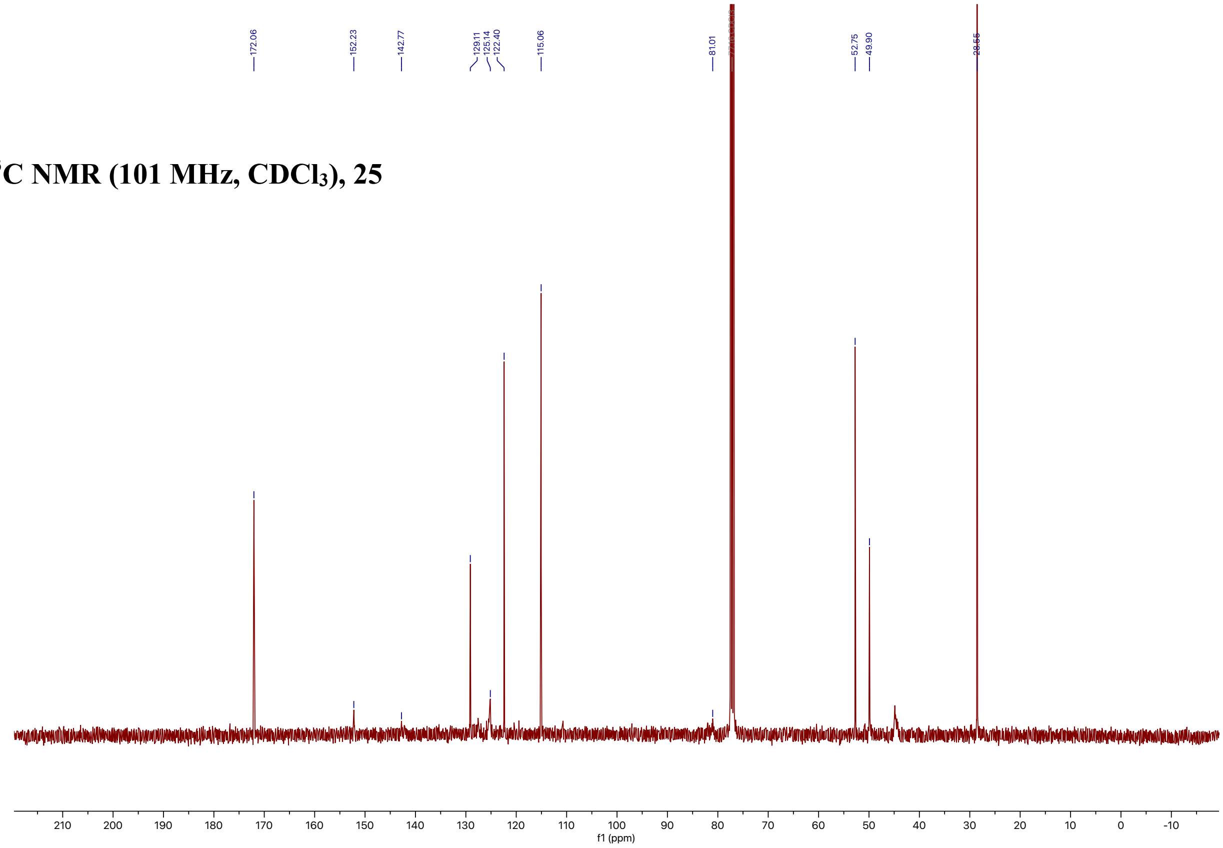

^{13}C NMR (101 MHz, $CDCl_3$), 25

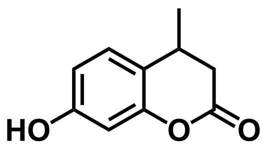

^{1}H NMR (400 MHz, $CDCl_3$), 26

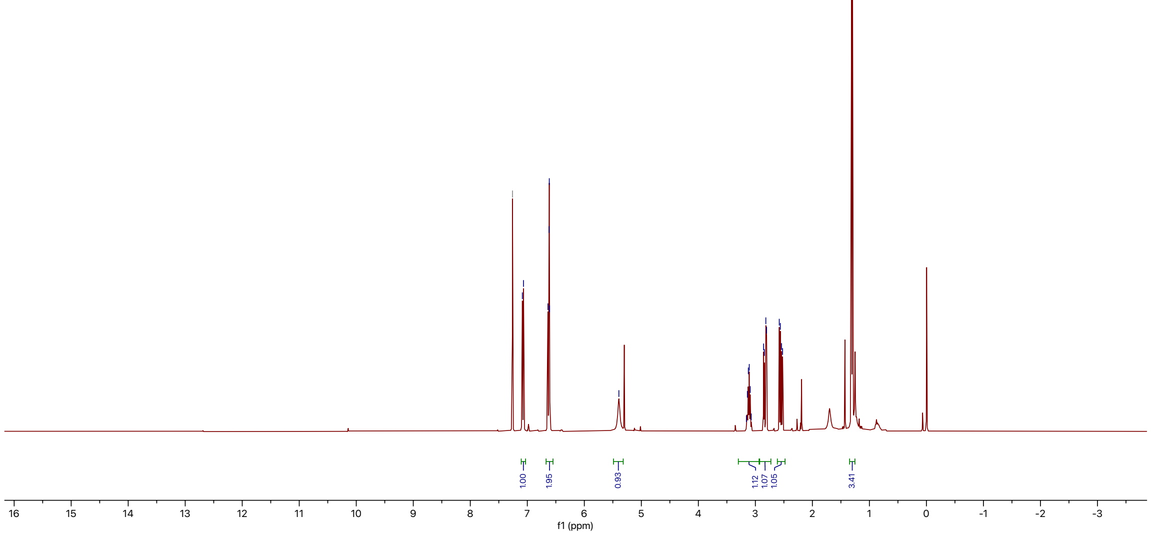

^{13}C NMR (101 MHz, $CDCl_3$), 26

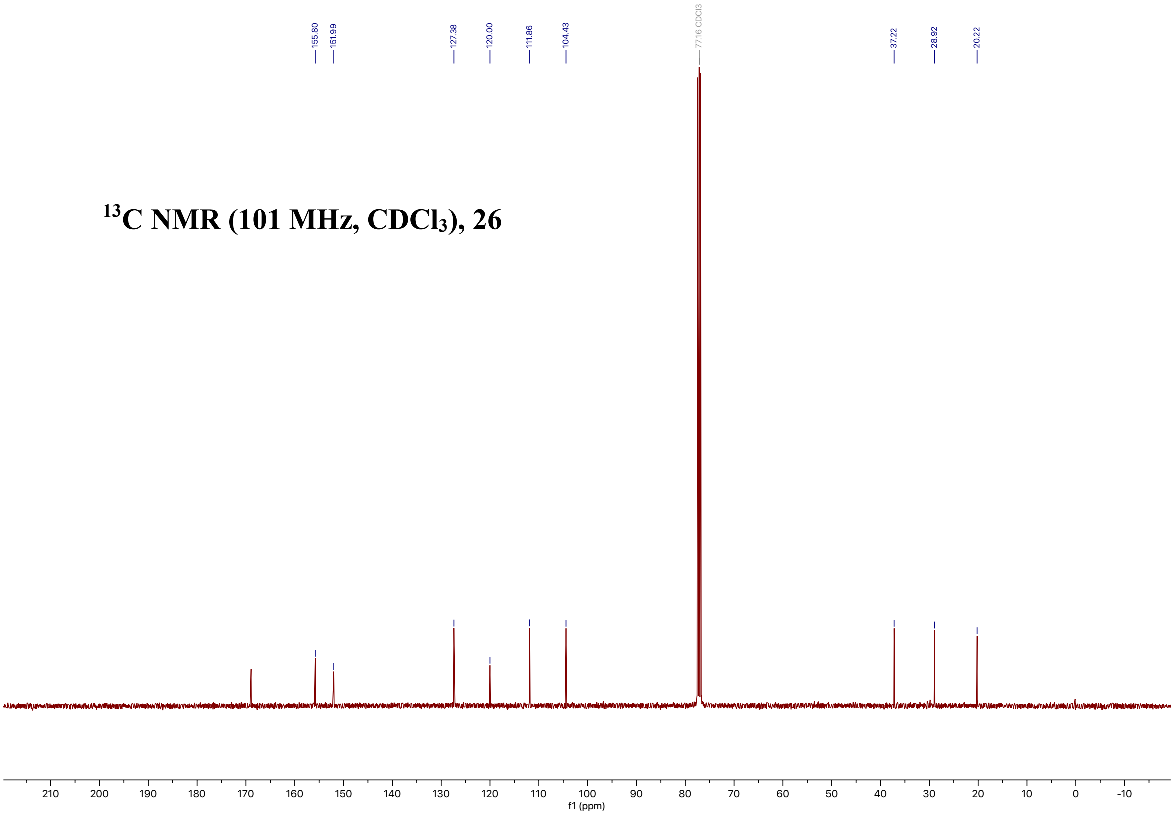

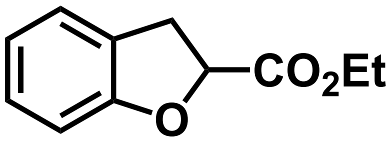

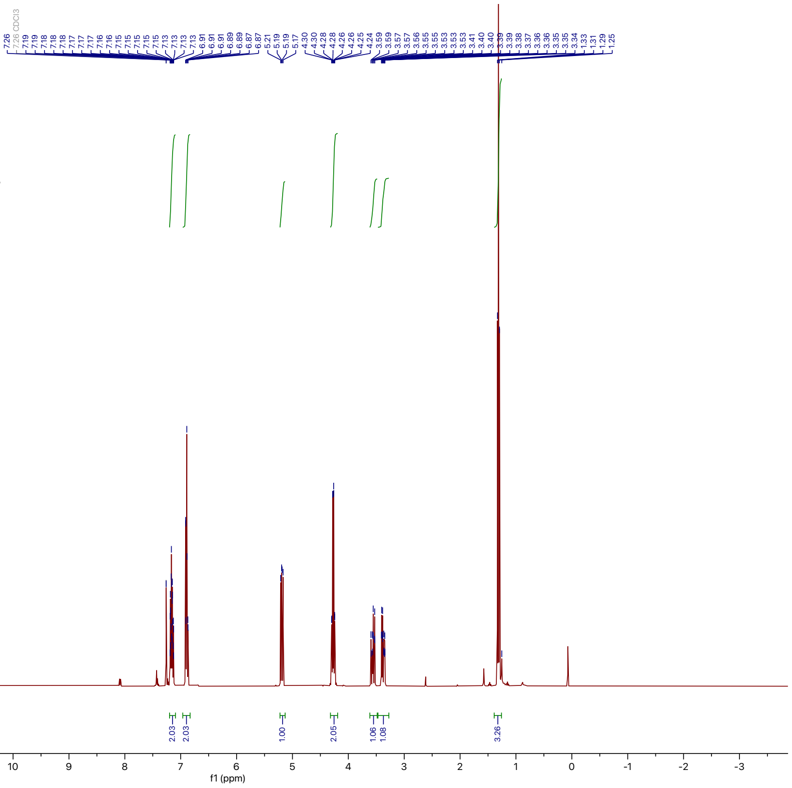

^{1}H NMR (400 MHz, $CDCl_3$), 27

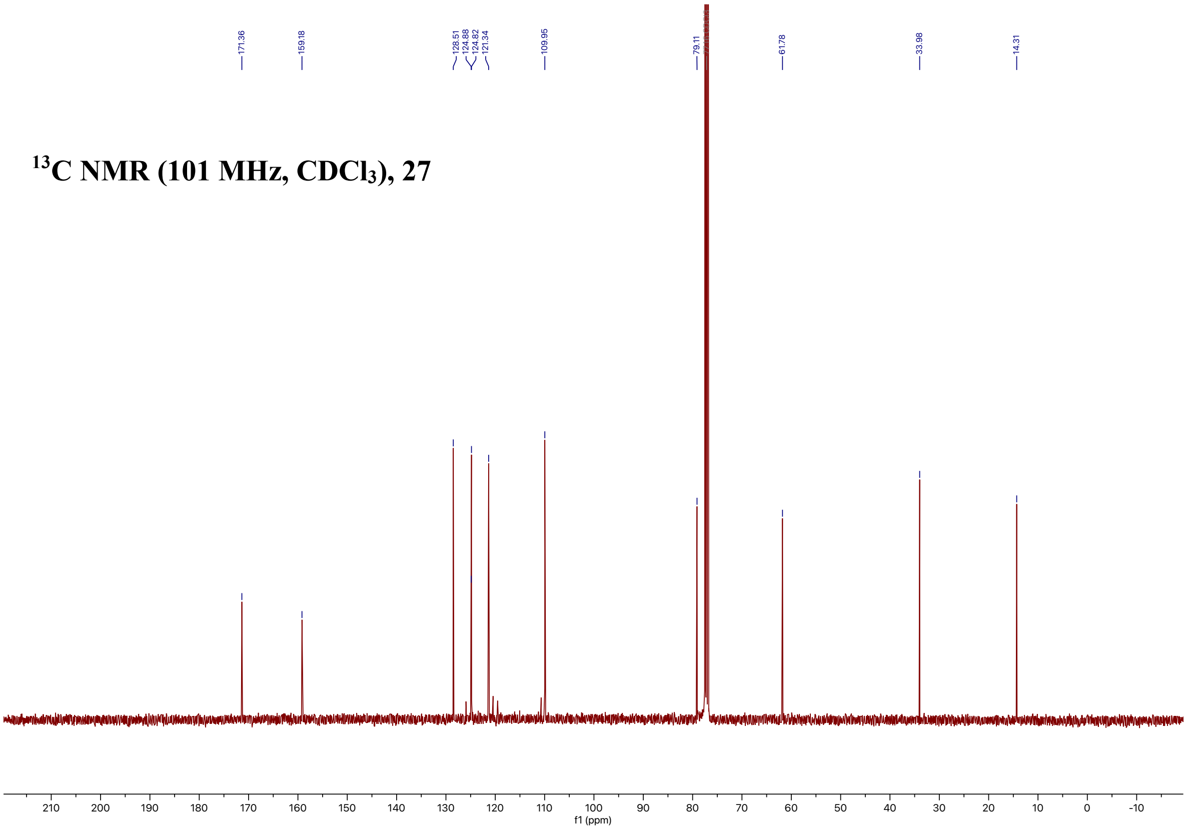

^{13}C NMR (101 MHz, $CDCl_3$), 27

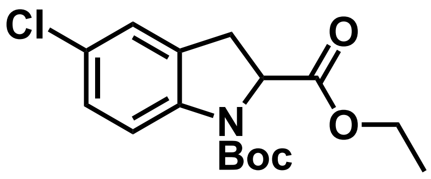

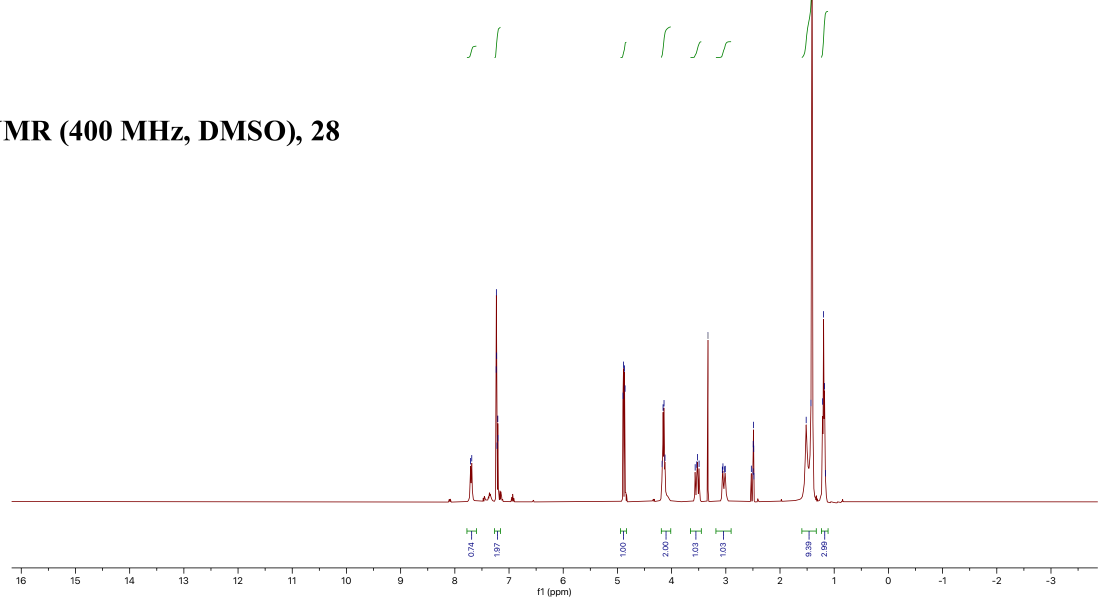

^{1}H NMR (400 MHz, DMSO), 28

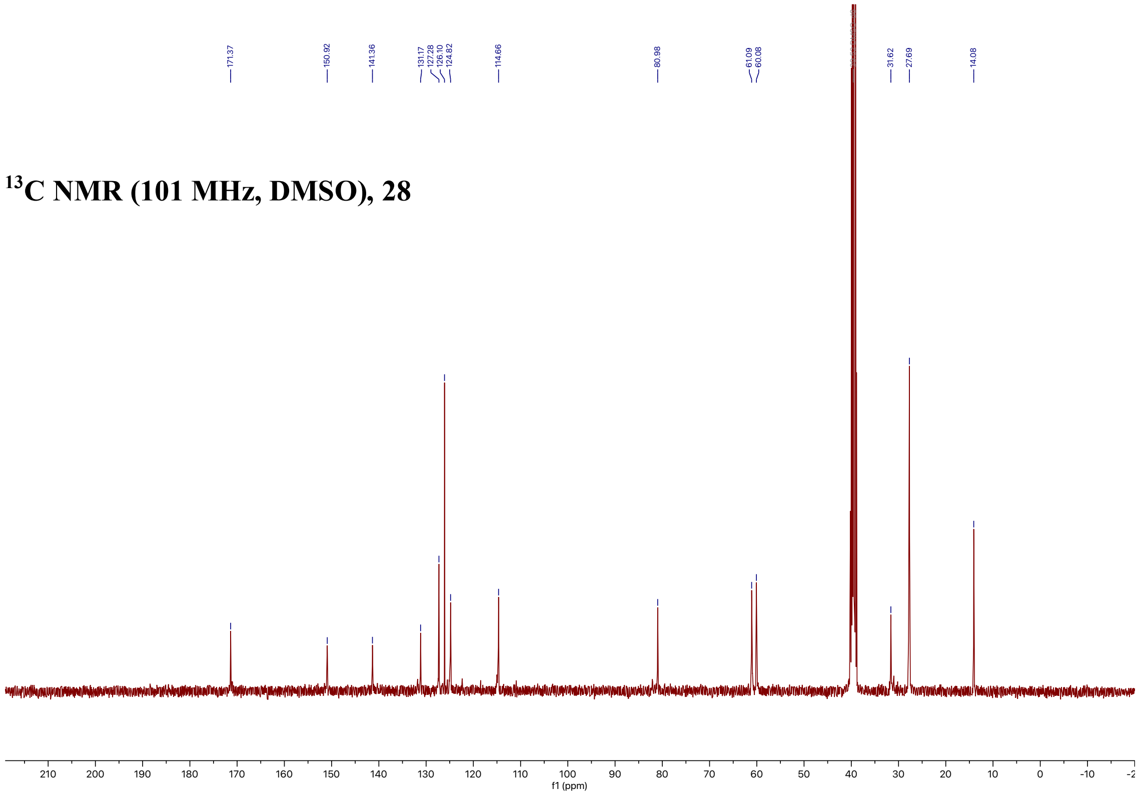

^{13}C NMR (101 MHz, DMSO), 28

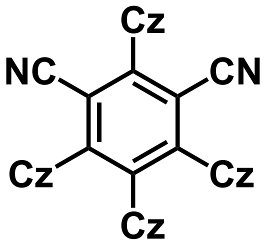

^{1}H NMR (400 MHz, $CDCl_3$), 29

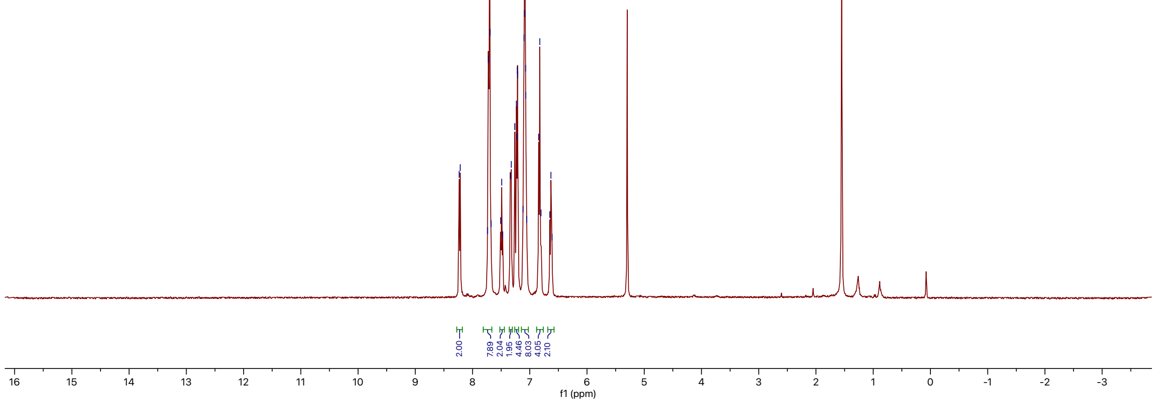

^{13}C NMR (101 MHz, $CDCl_3$)

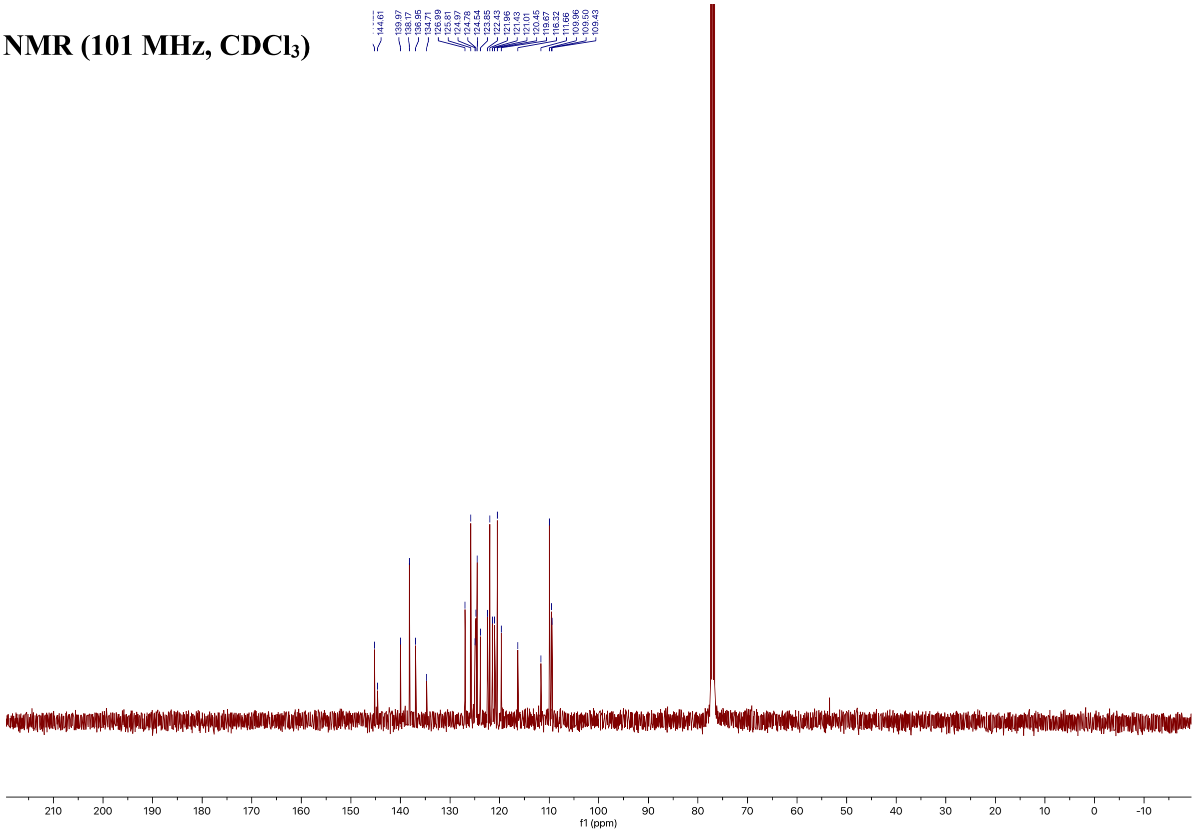

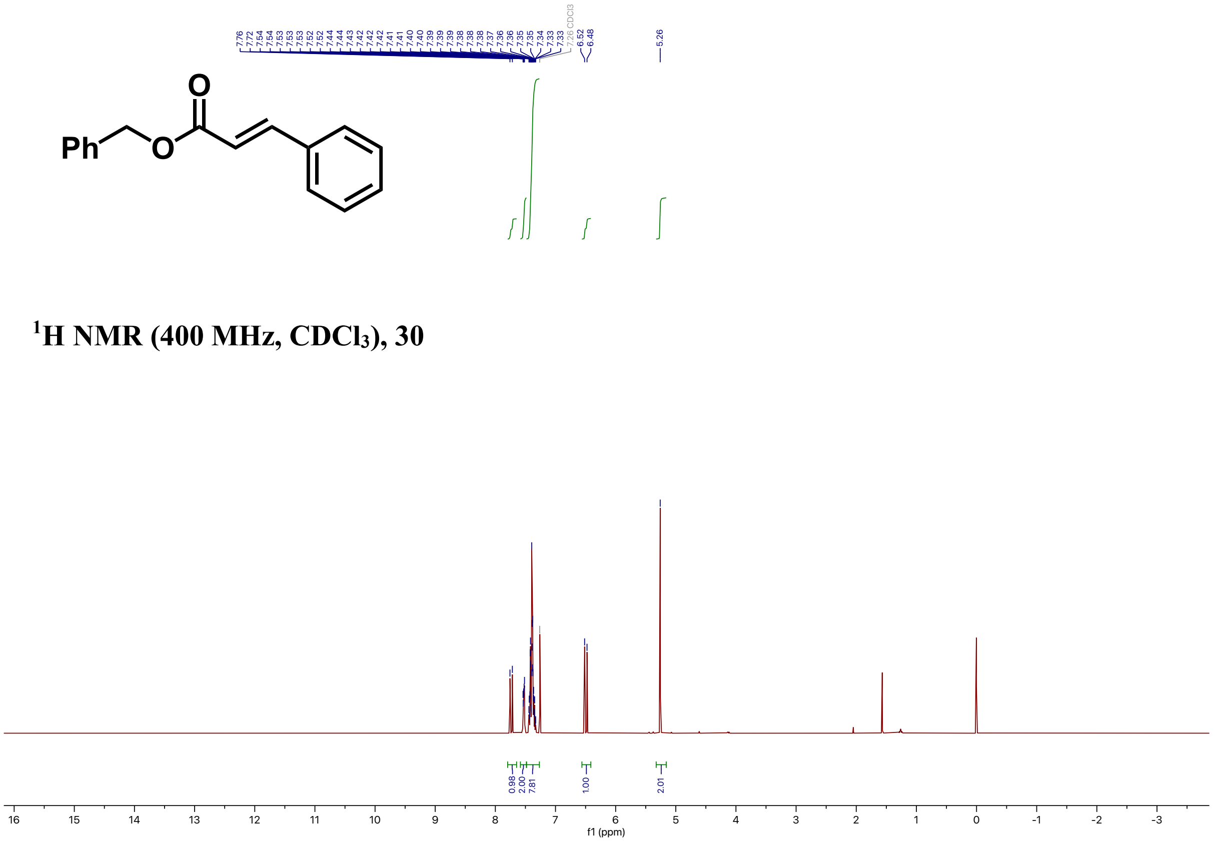

^{1}H NMR (400 MHz, CDCl$_3$), 30

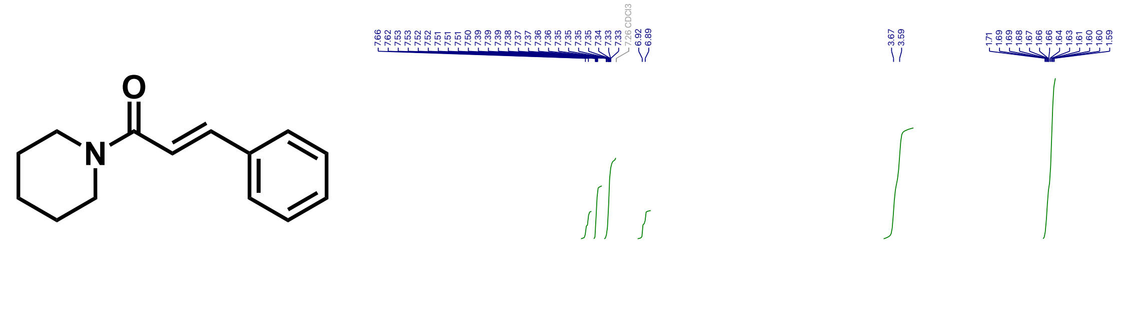

^{1}H NMR (400 MHz, CDCl$_3$), 31

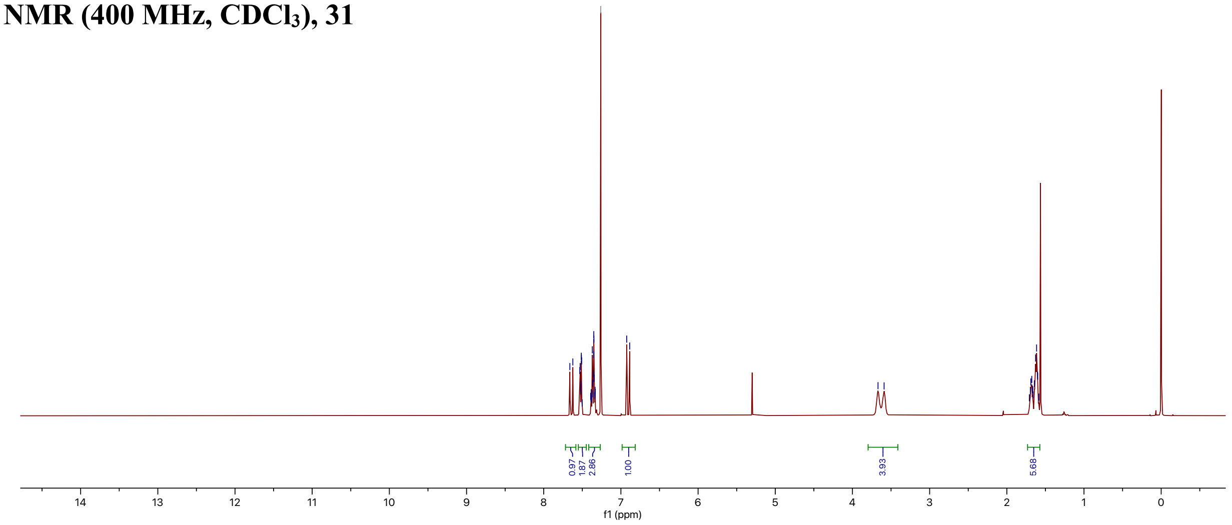

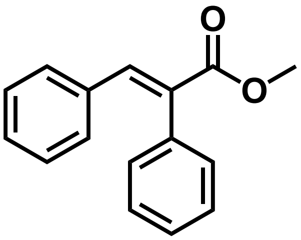

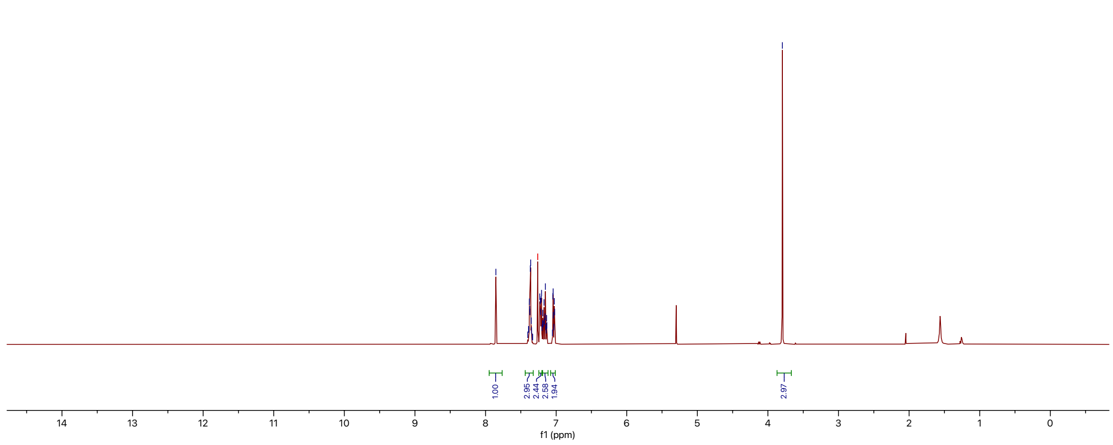

^{1}H NMR (400 MHz, $CDCl_3$), 32

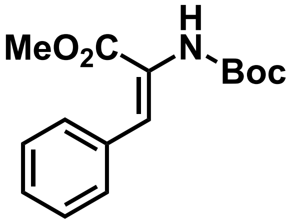

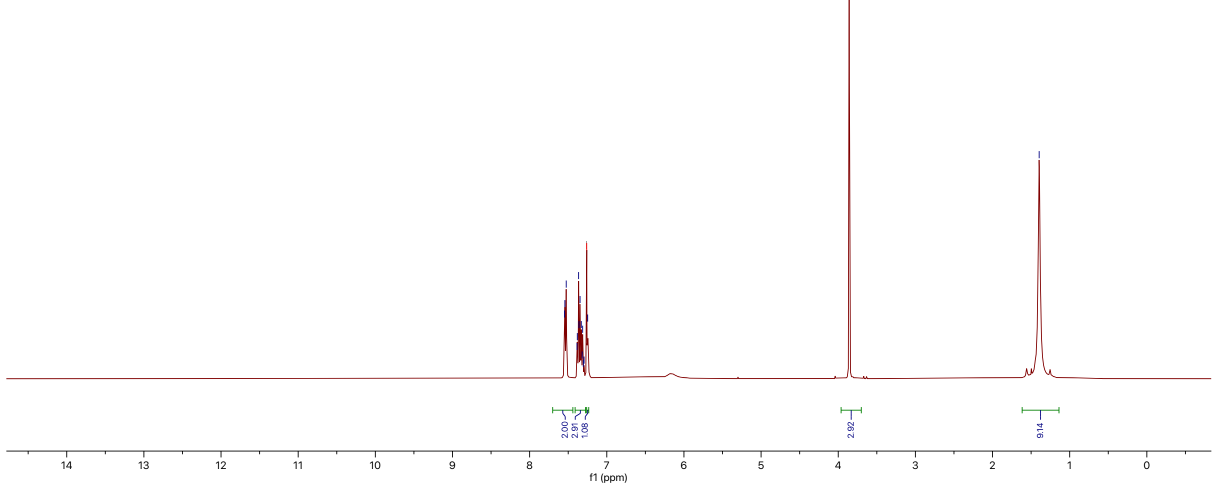

^{1}H NMR (400 MHz, $CDCl_3$), 33

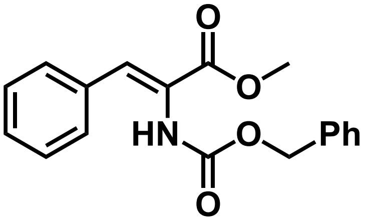

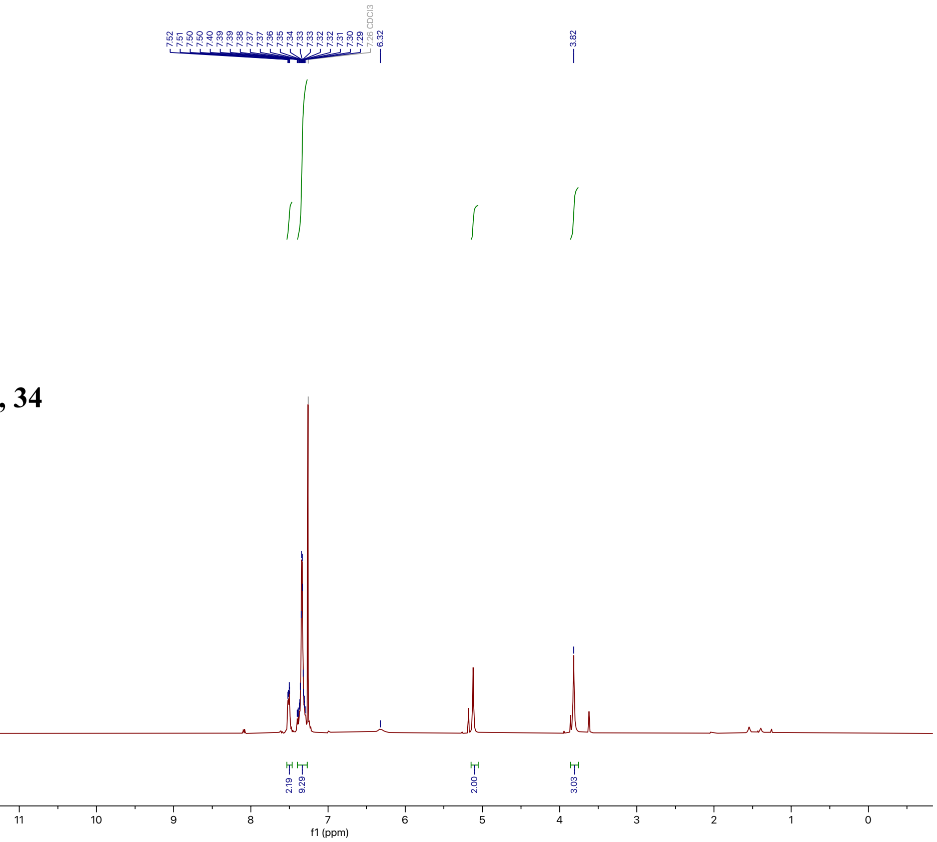

^{1}H NMR (400 MHz, CDCl$_3$), 34

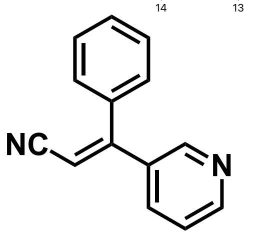

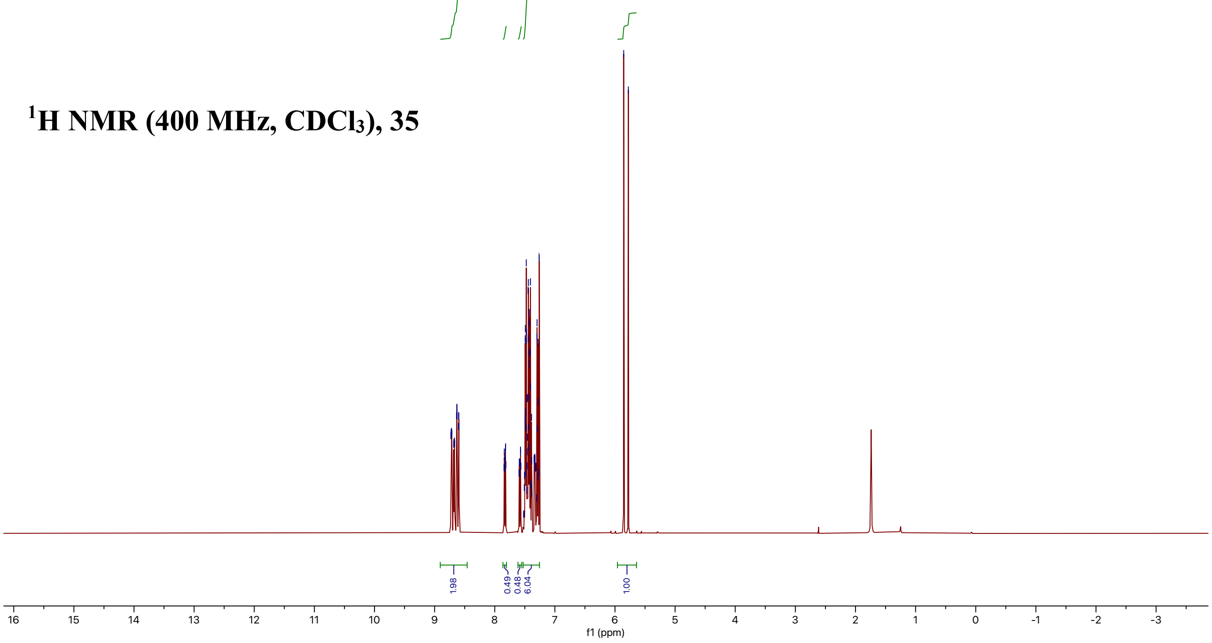

^{1}H NMR (400 MHz, CDCl$_3$), 35

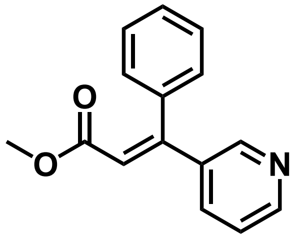

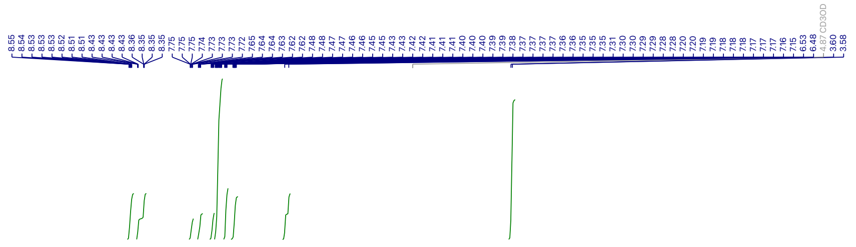

^{1}H NMR (400 MHz, MeOD), 36

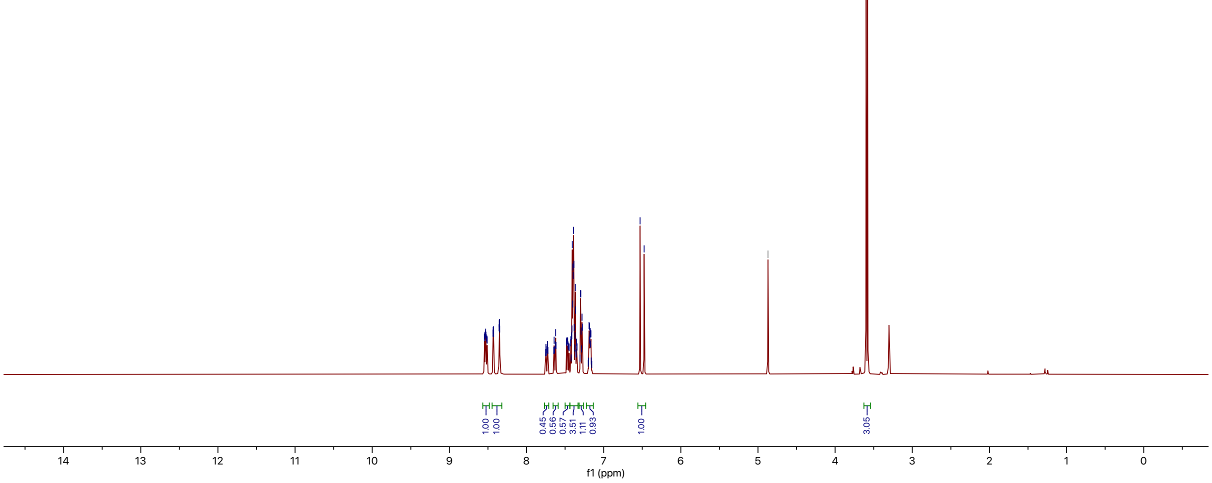

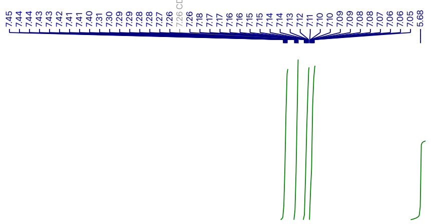

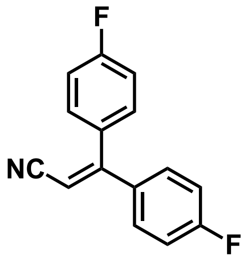

^{1}H NMR (400 MHz, $CDCl_3$), 37

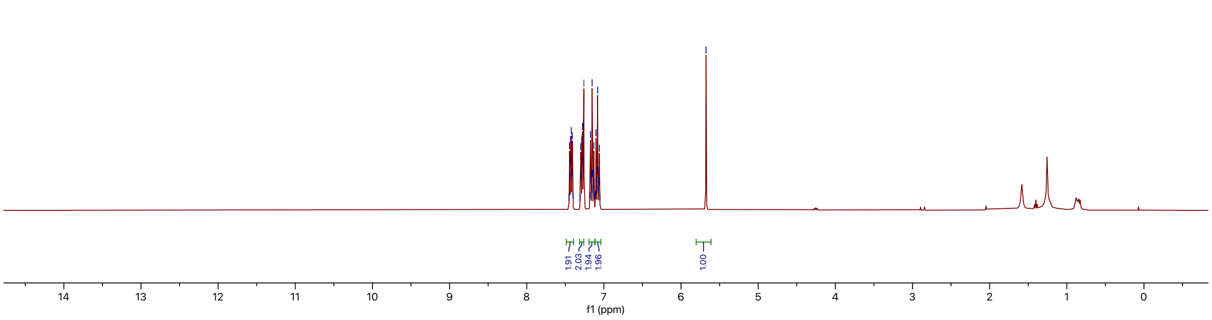

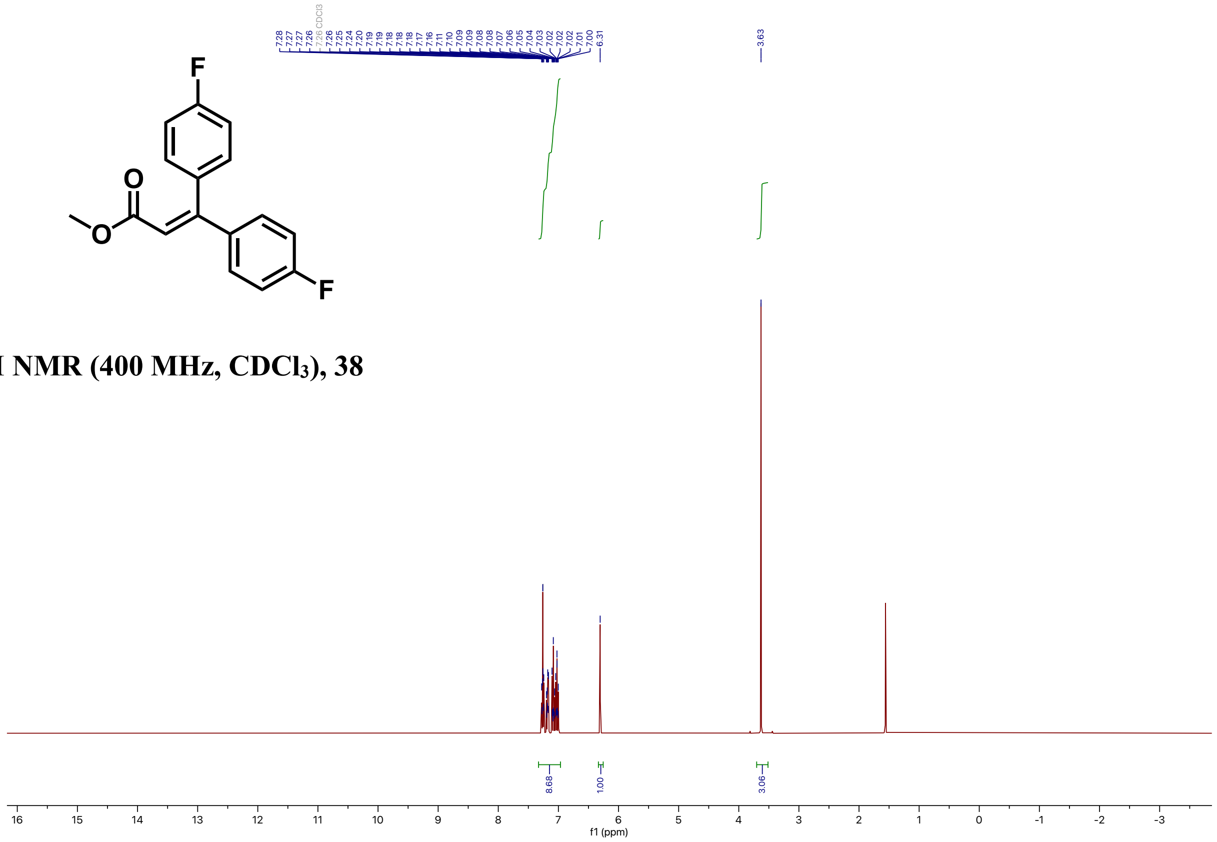

^{1}H NMR (400 MHz, $CDCl_3$), 38

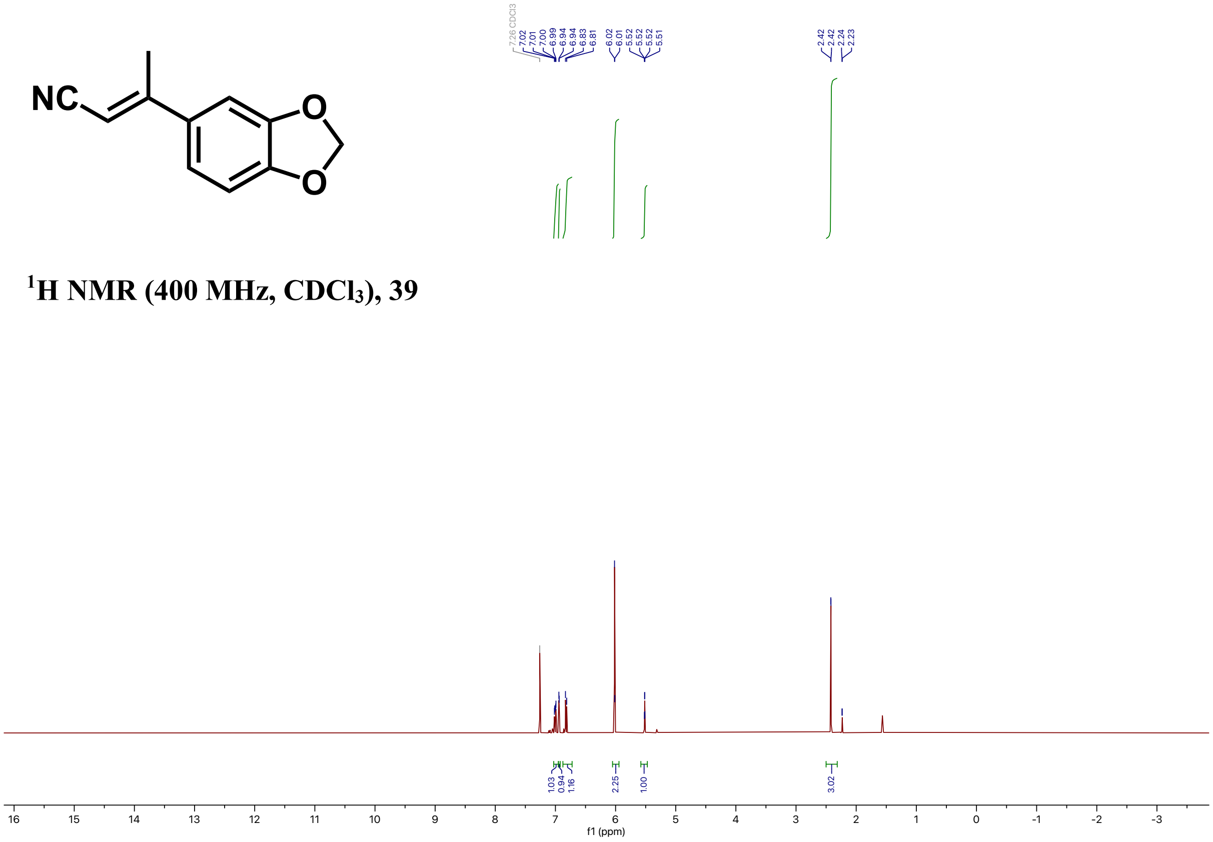

^{1}H NMR (400 MHz, $CDCl_3$), 39

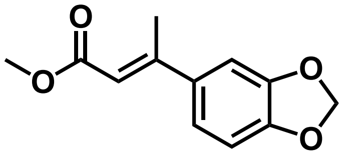

^{1}H NMR (400 MHz, CDCl$_3$), 40

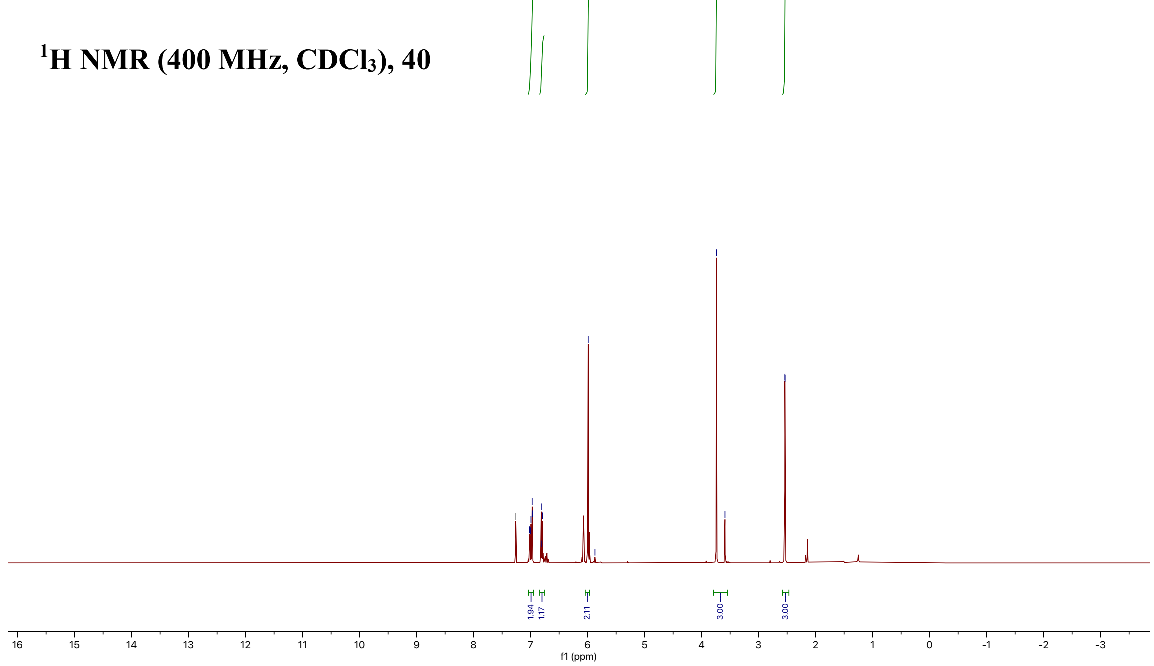

^{13}C NMR (101 MHz, CDCl$_3$), 40

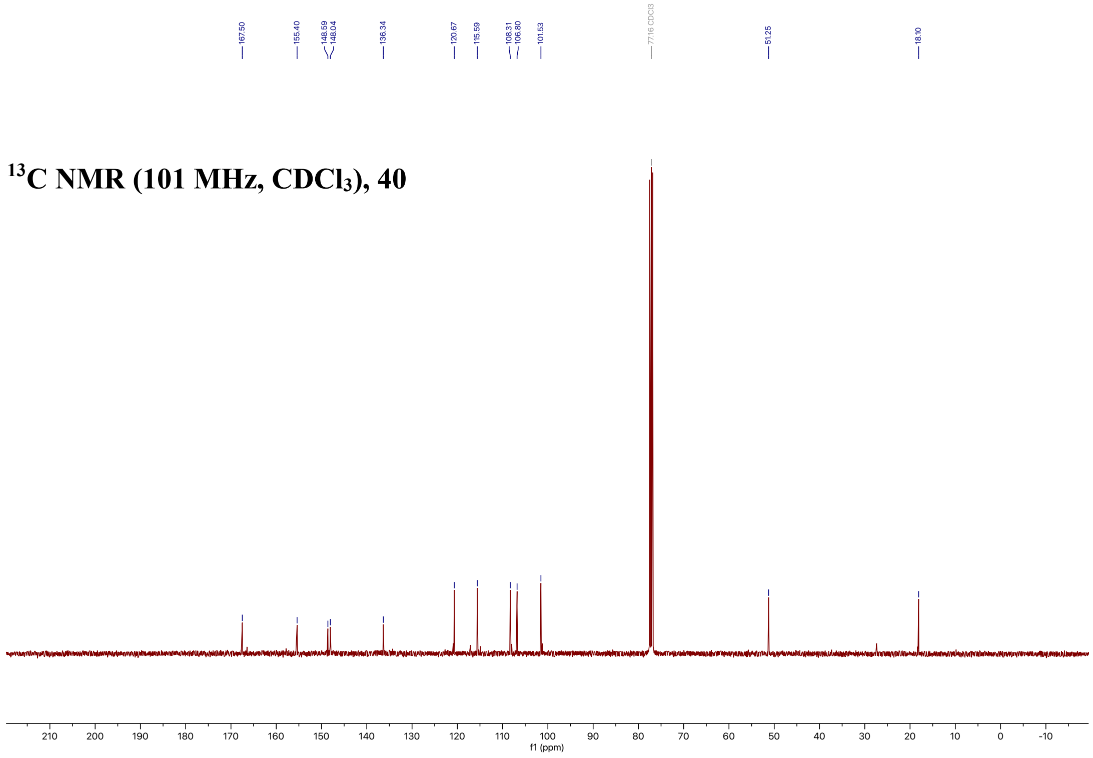

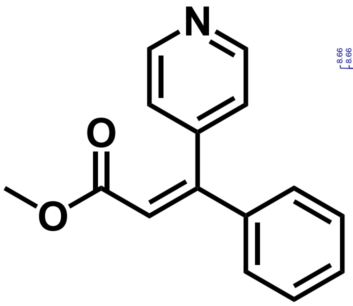

^{1}H NMR (400 MHz, CDCl$_3$), 41

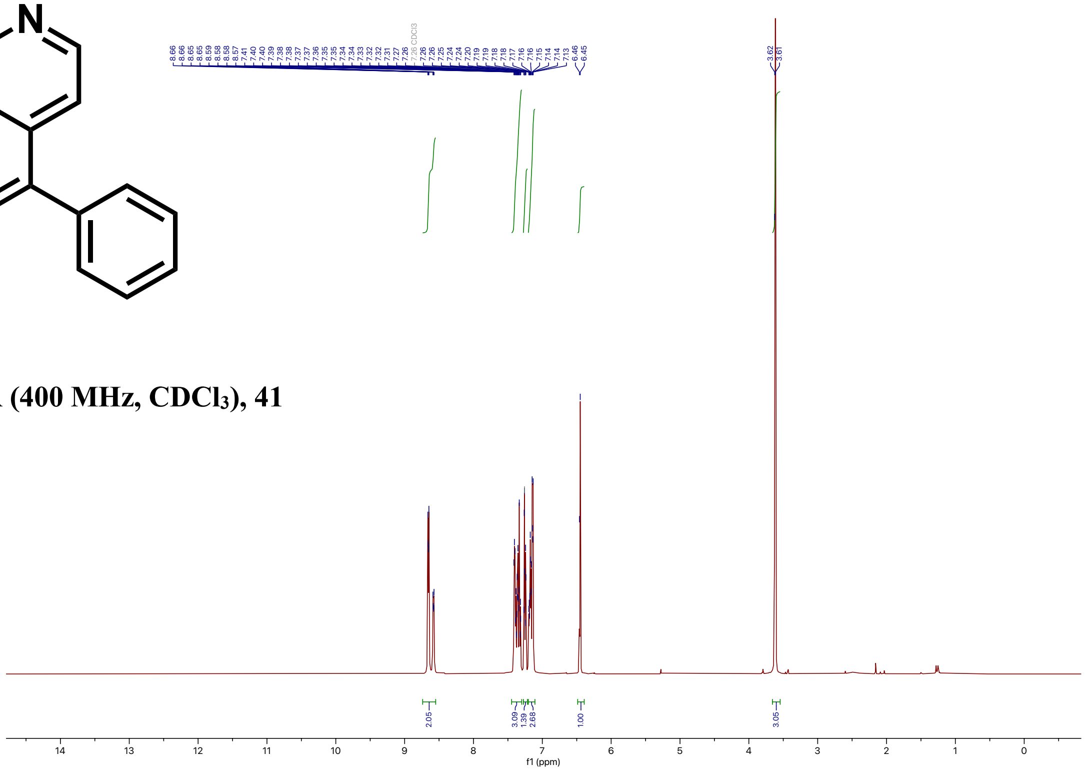

^{13}C NMR (101 MHz, CDCl$_3$), 41

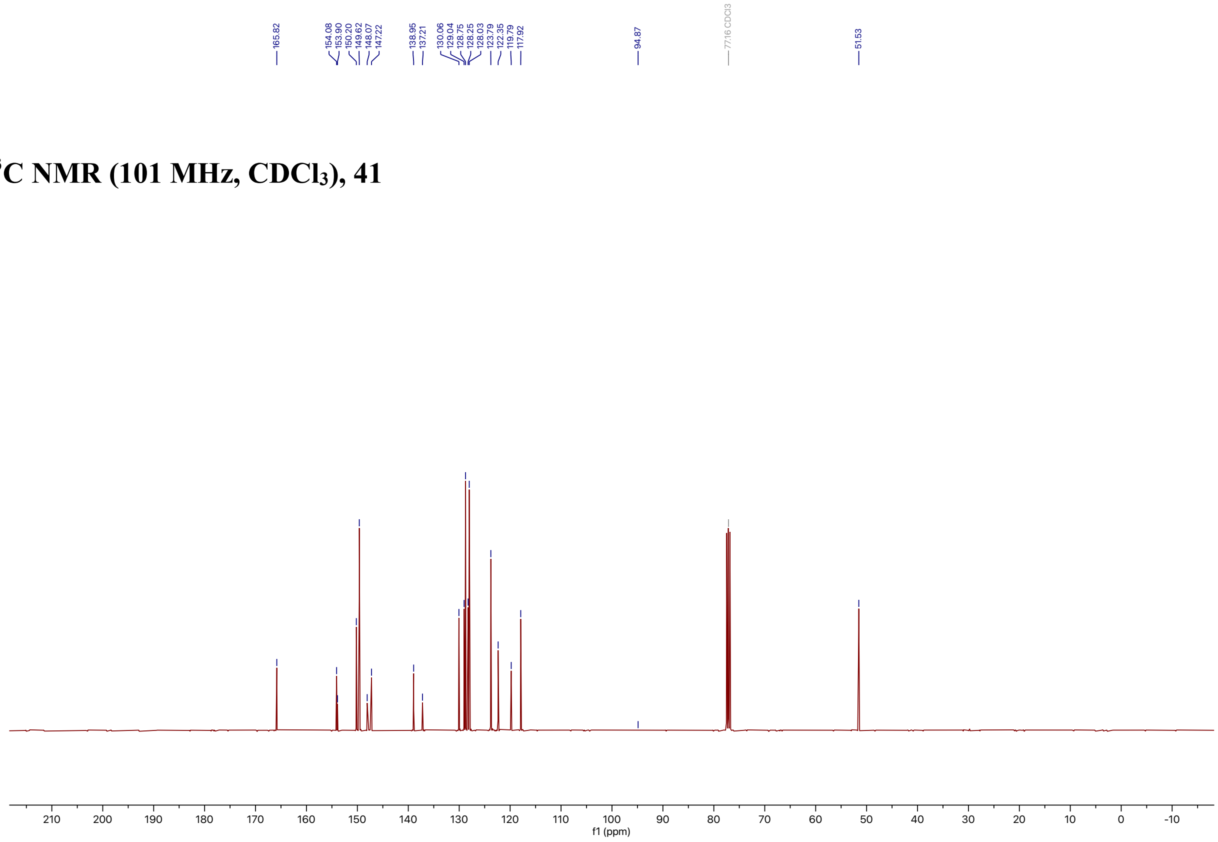

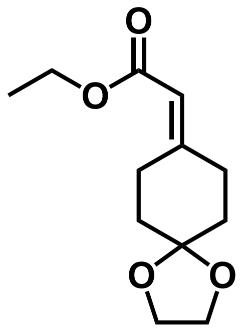

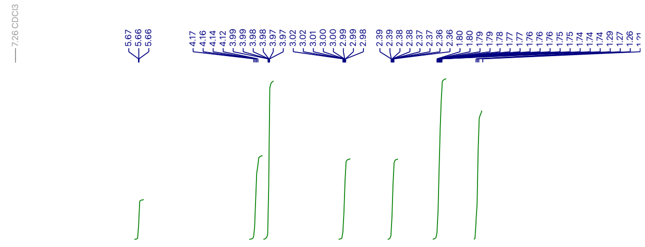

^{1}H NMR (400 MHz, $CDCl_3$), 42

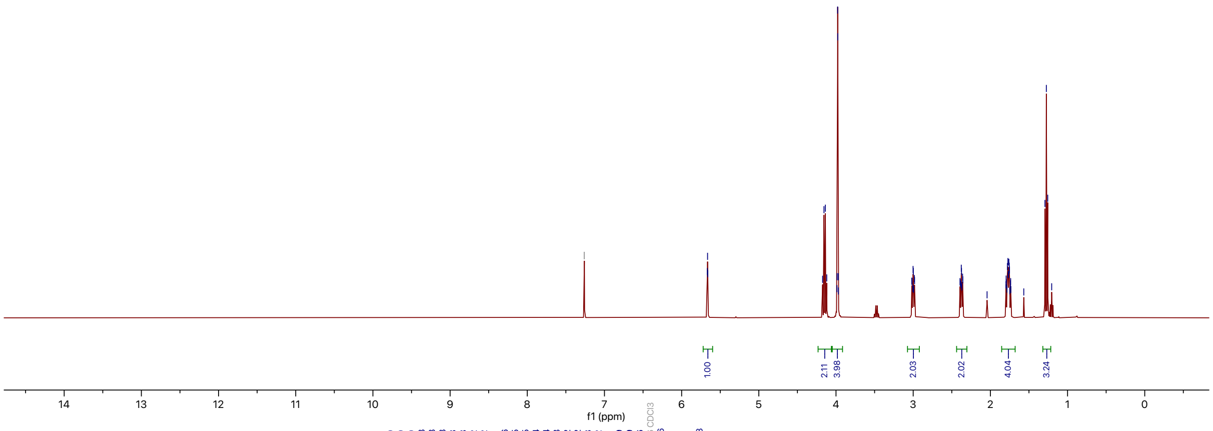

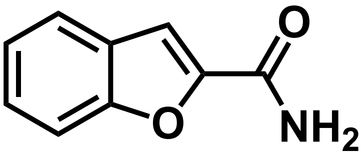

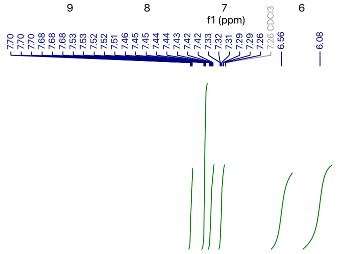

^{1}H NMR (400 MHz, $CDCl_3$), 43

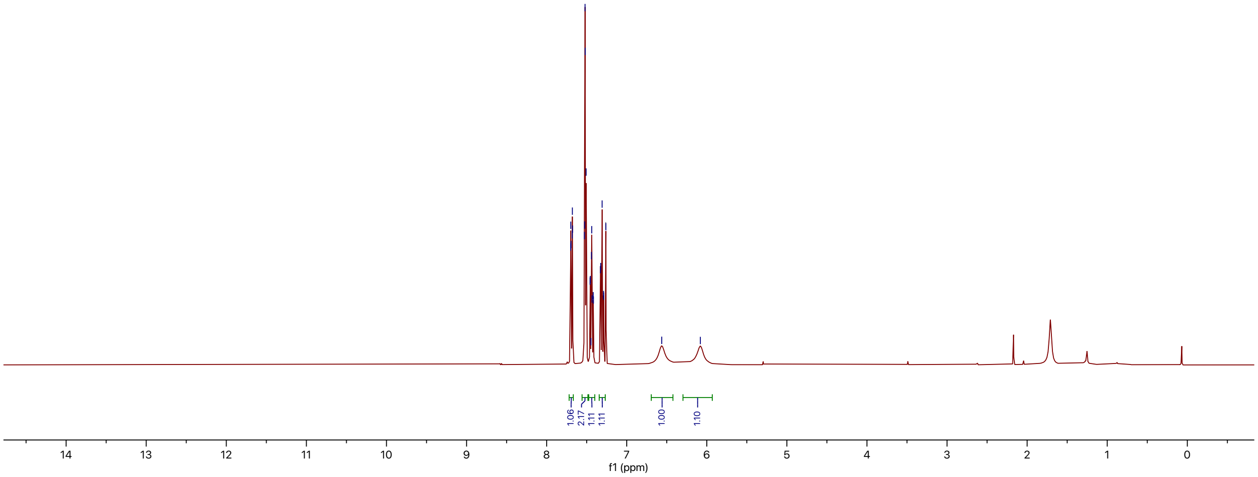

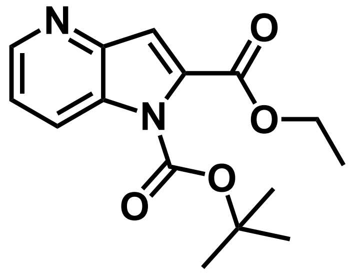

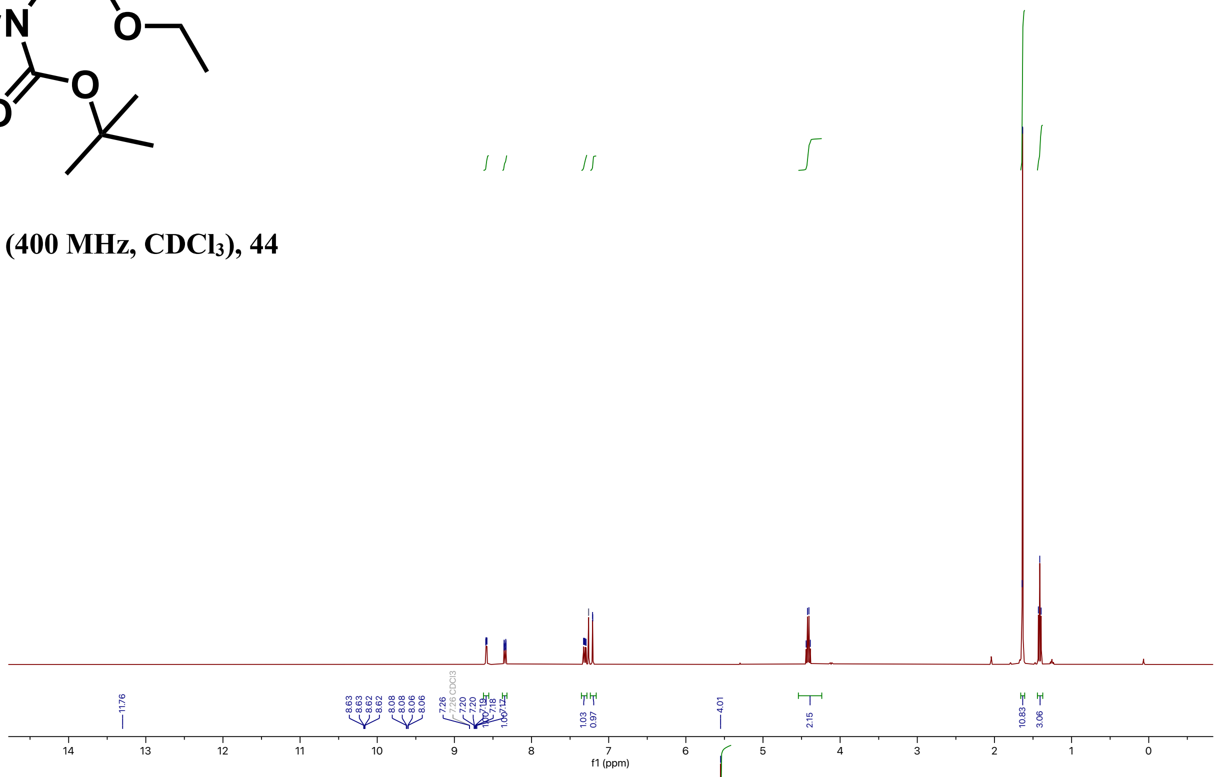

^{1}H NMR (400 MHz, $CDCl_3$), 44

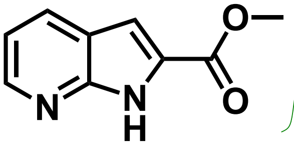

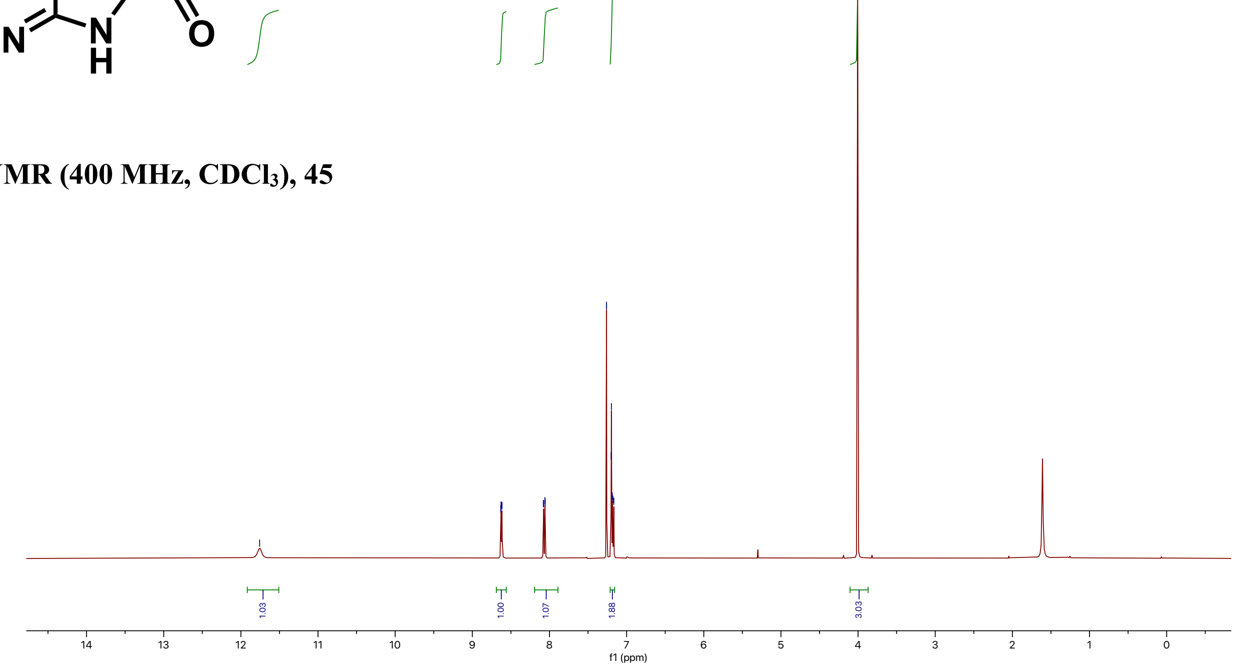

^{1}H NMR (400 MHz, $CDCl_3$), 45

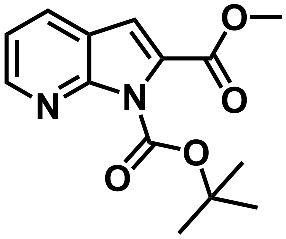

^{1}H NMR (400 MHz, CDCl$_3$), 46

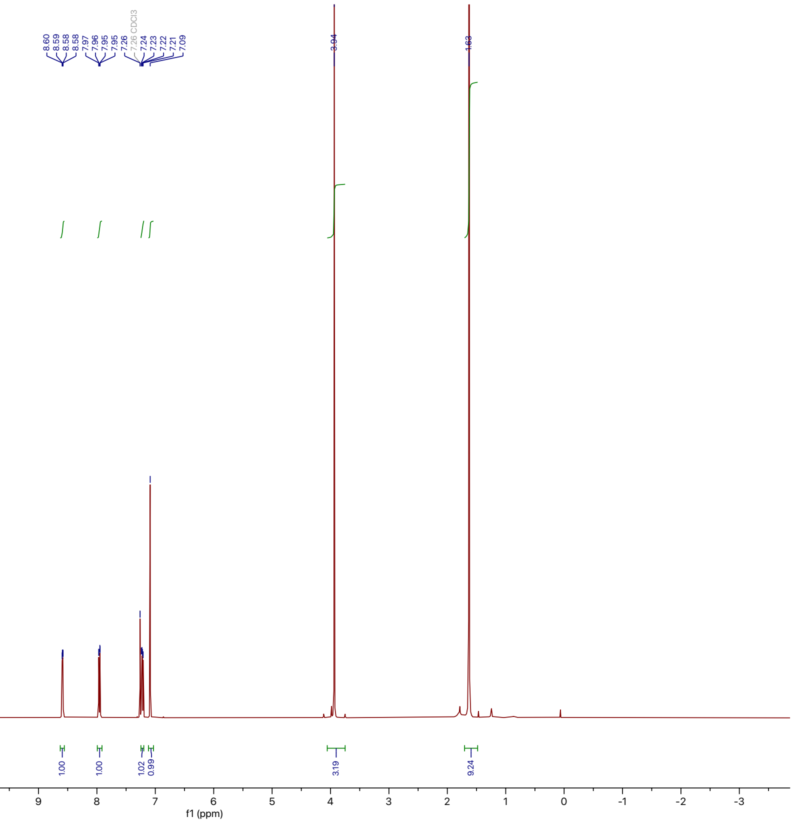

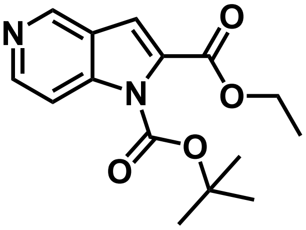

^{1}H NMR (400 MHz, CDCl$_3$), 47

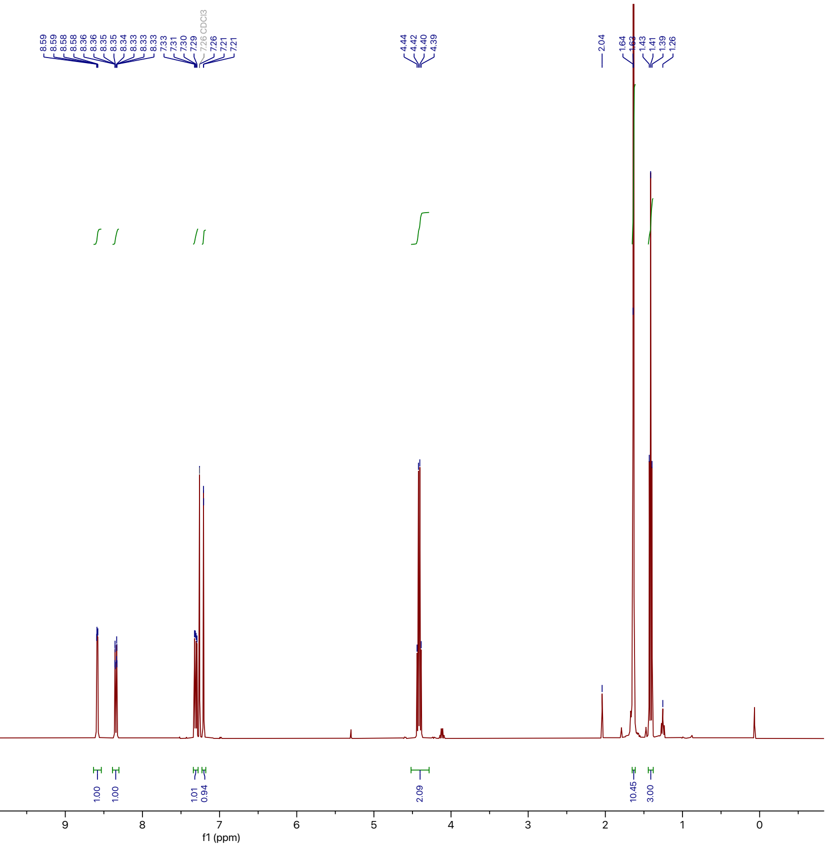

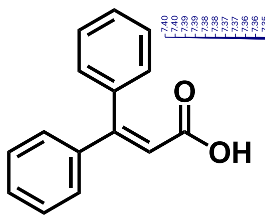

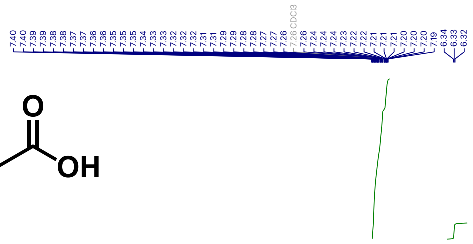

^{1}H NMR (400 MHz, $CDCl_3$), 48

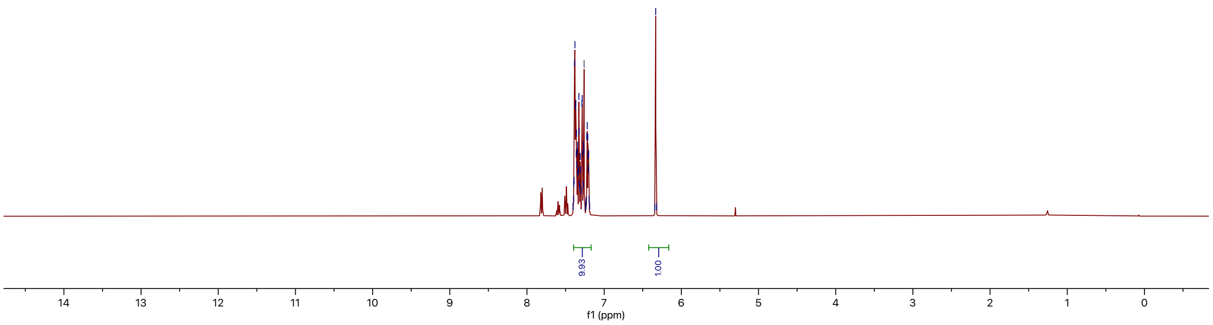

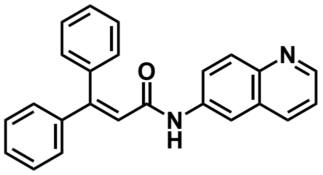

^{1}H NMR (400 MHz, $CDCl_3$), 49

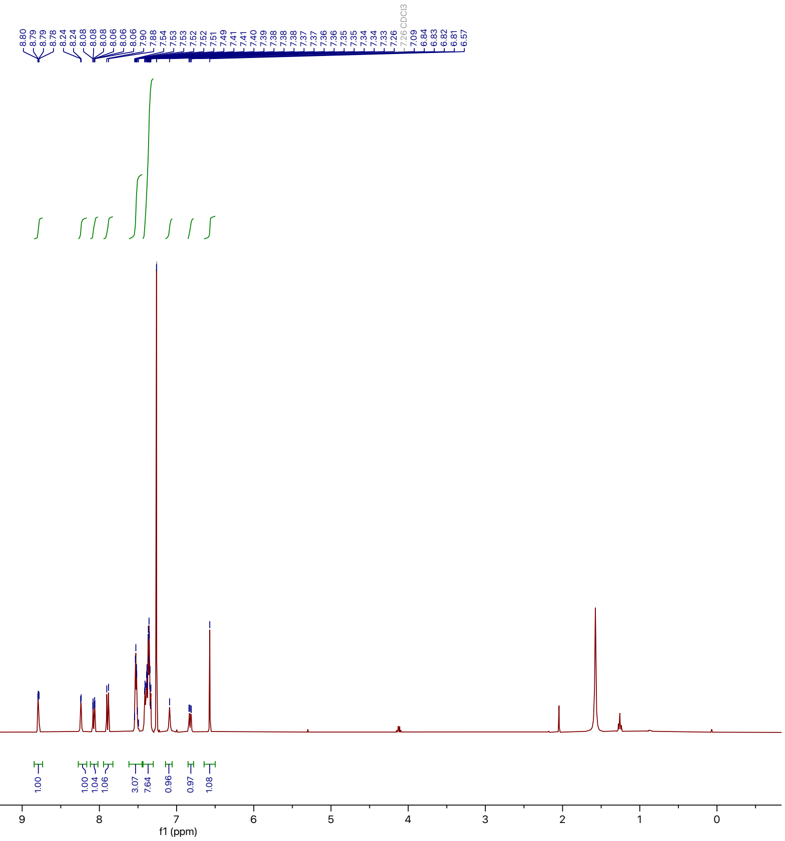

^{13}C NMR (101 MHz, $CDCl_3$), 49

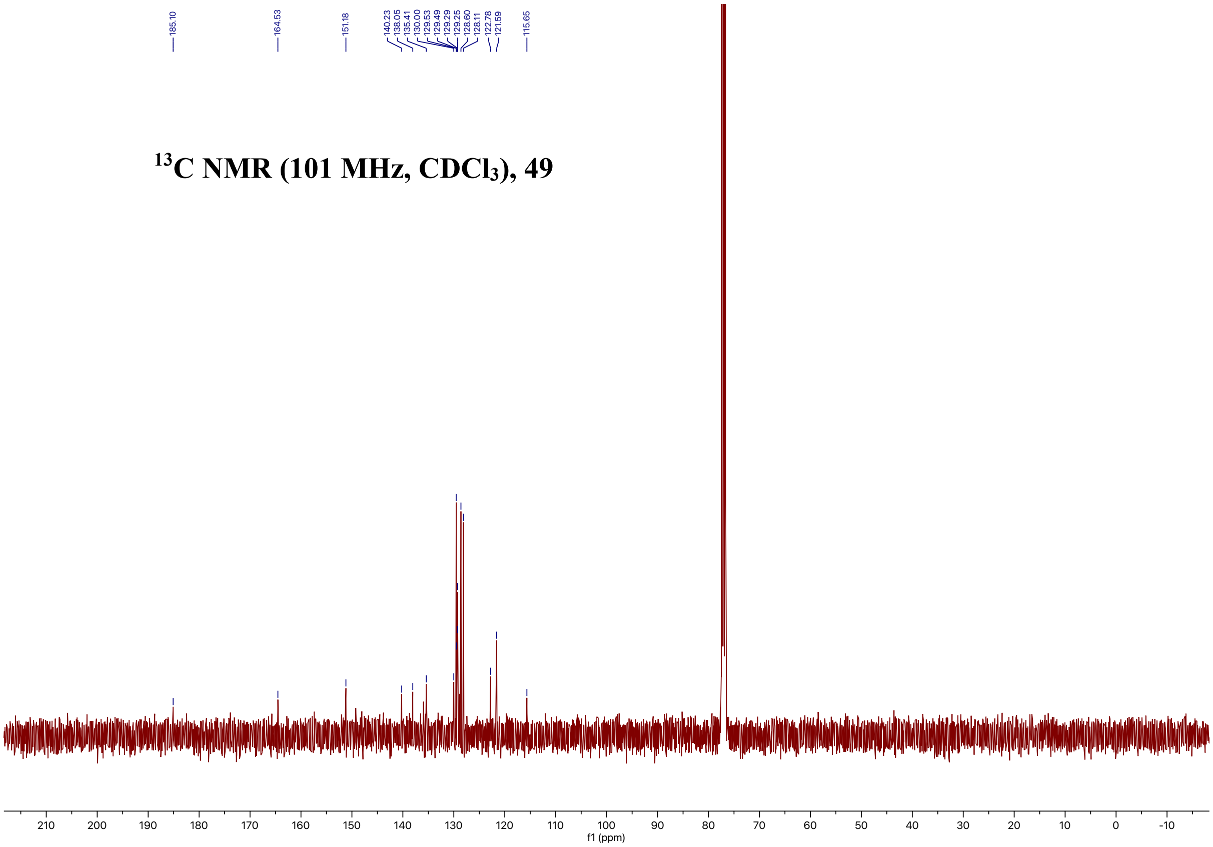

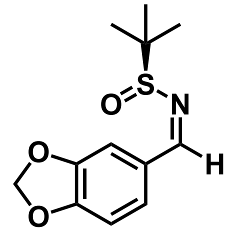

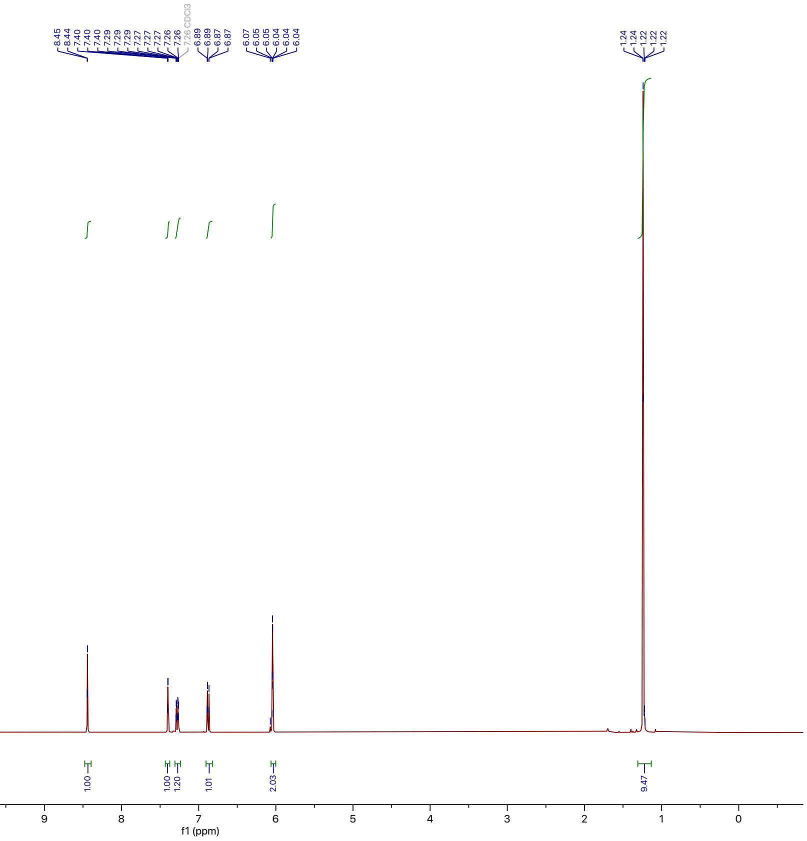

^{1}H NMR (400 MHz, $CDCl_3$), 50

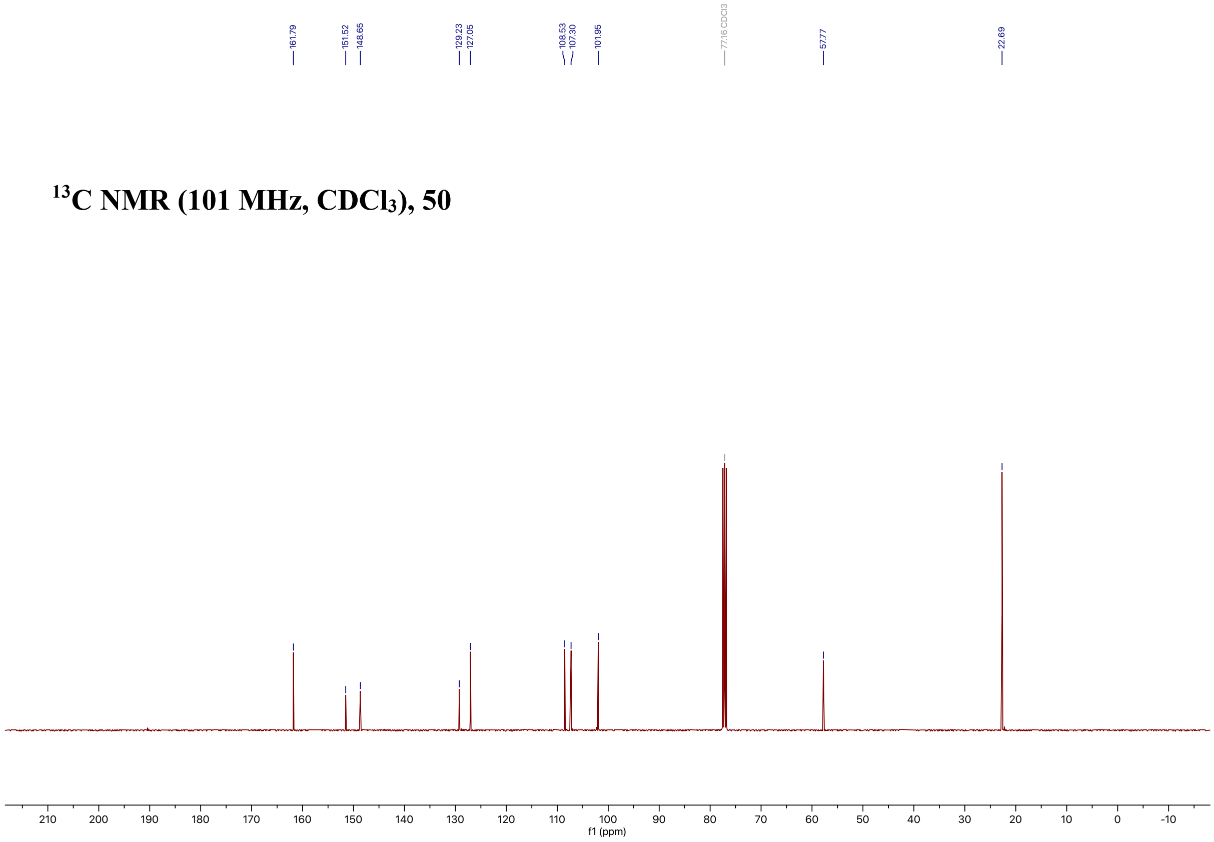

^{13}C NMR (101 MHz, $CDCl_3$), 50

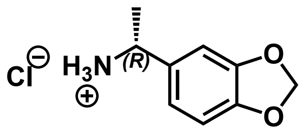

^{1}H NMR (400 MHz, DMSO), 51

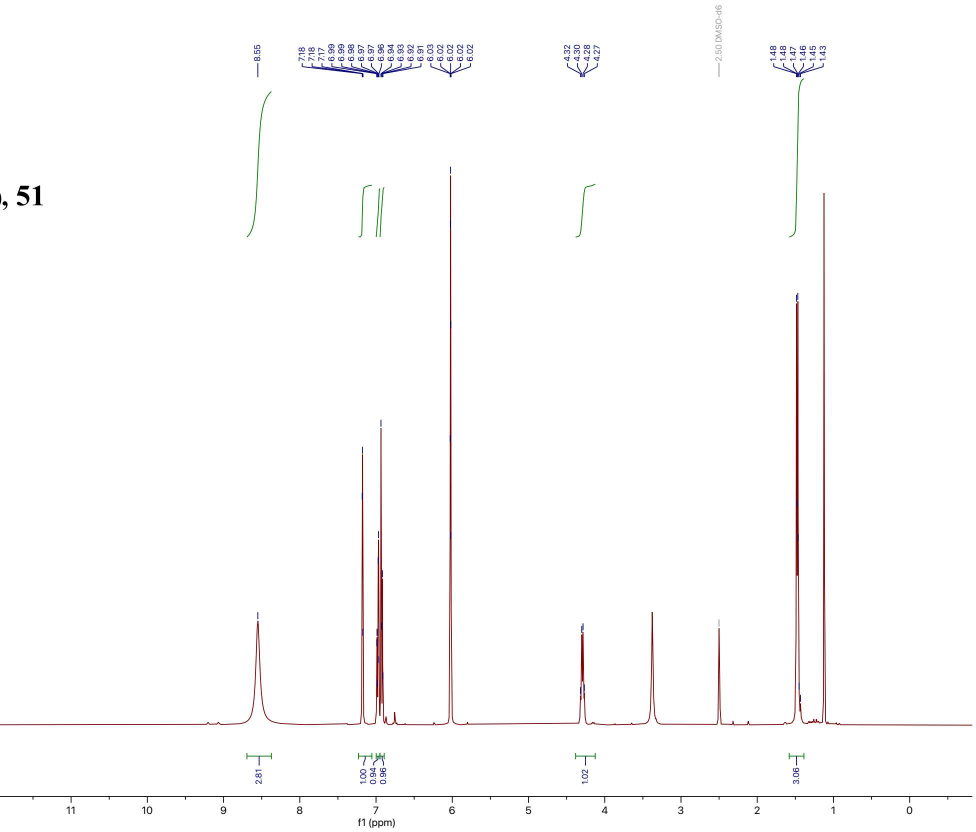

^{13}C NMR (151 MHz, DMSO), 51

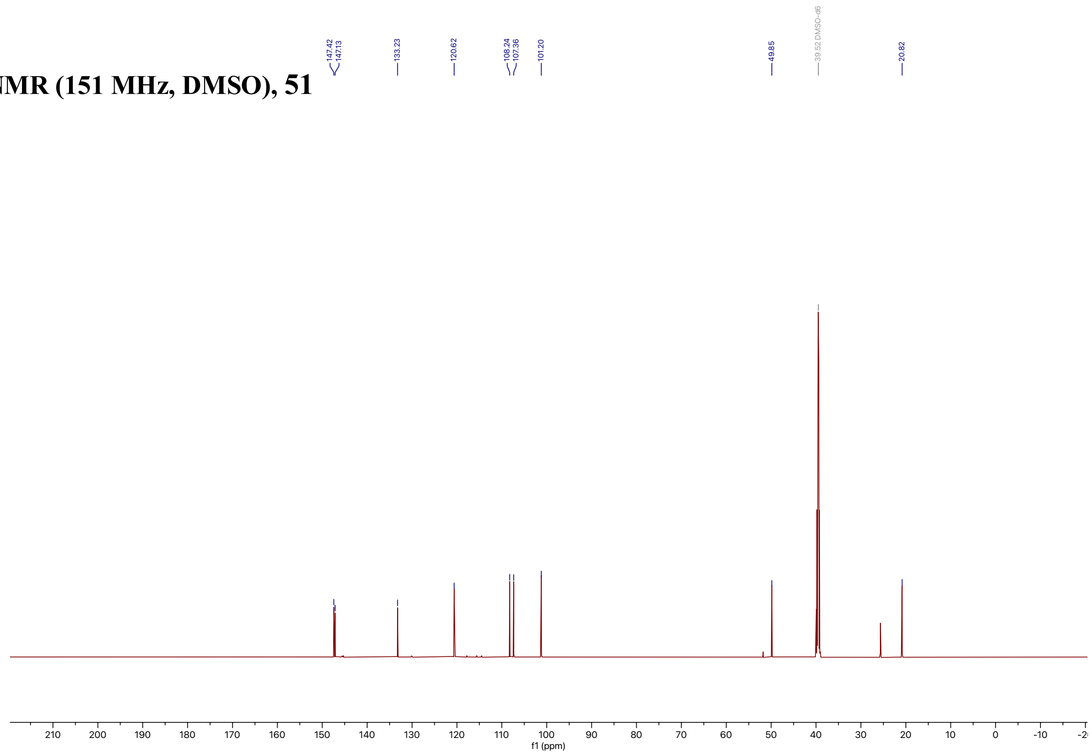

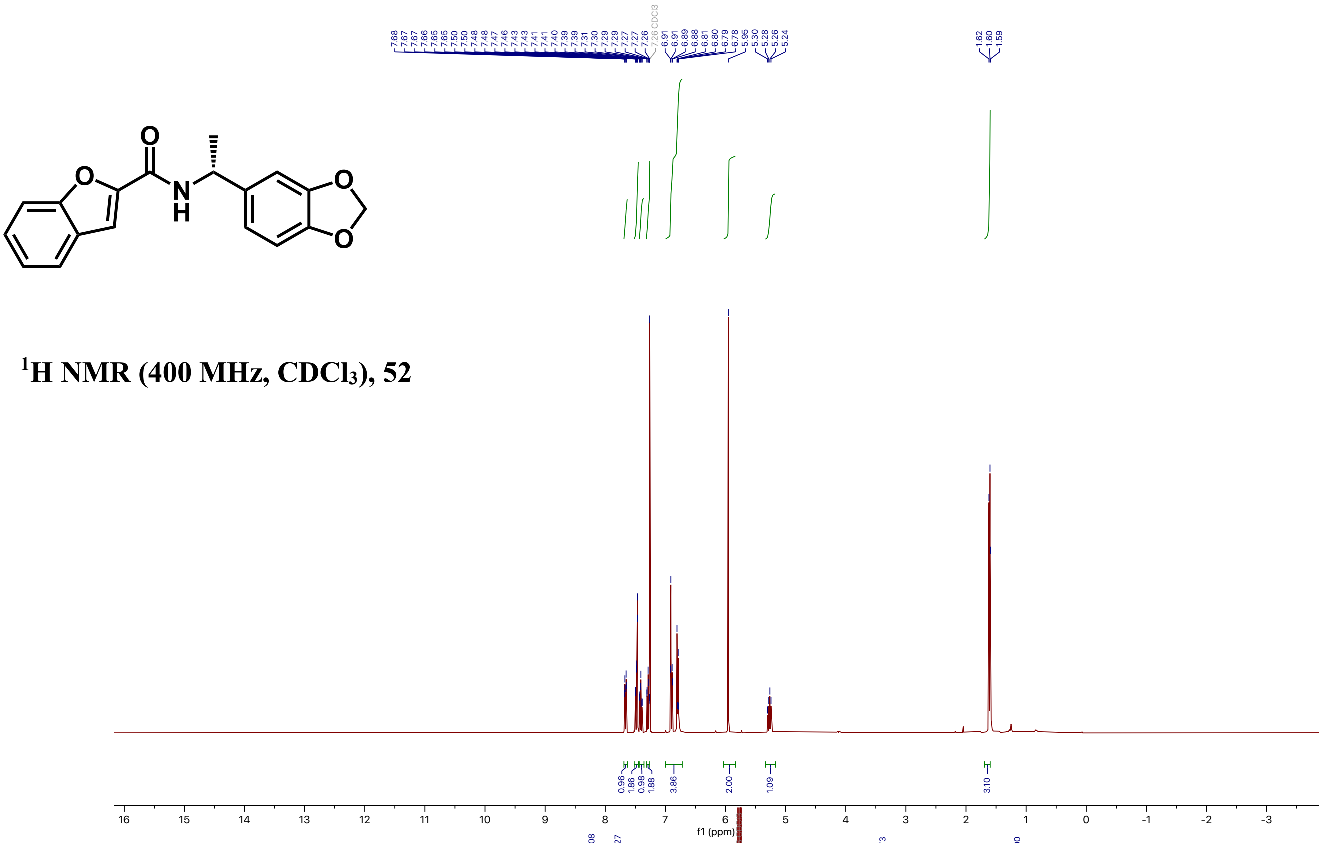

^{1}H NMR (400 MHz, $CDCl_3$), 52

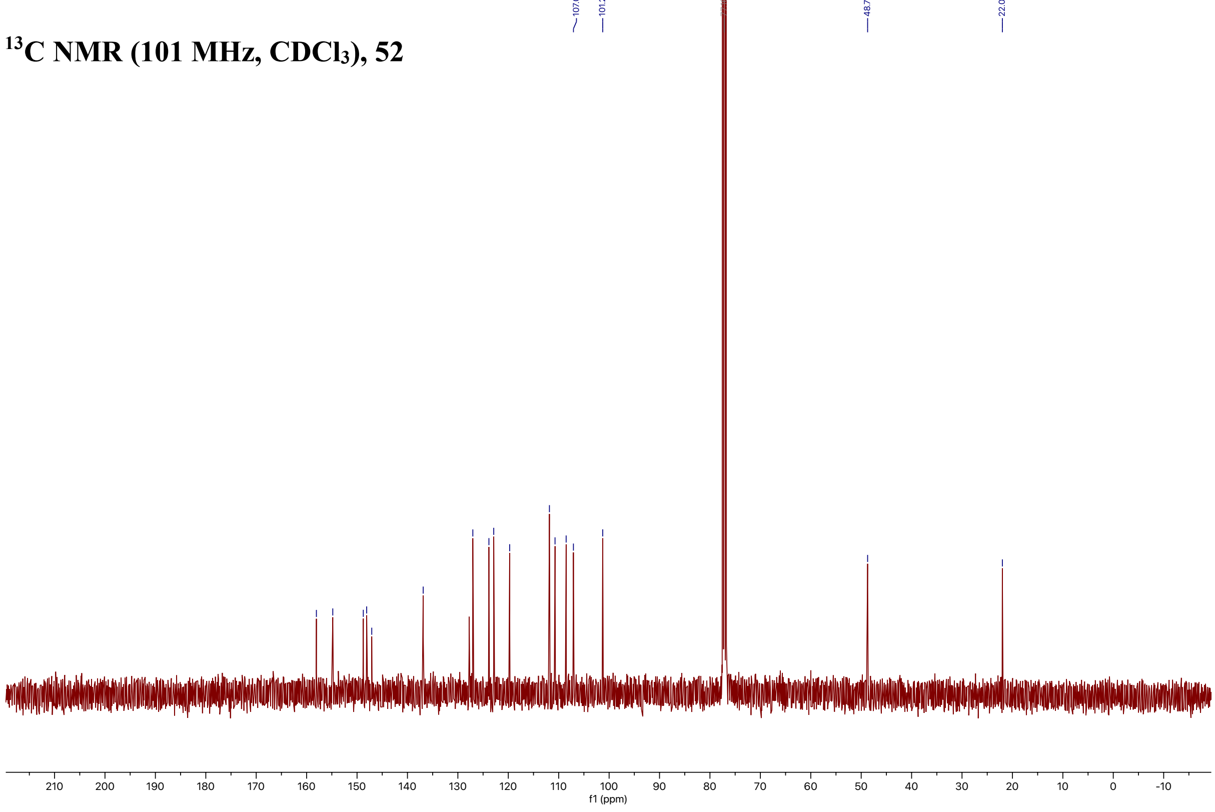

^{13}C NMR (101 MHz, $CDCl_3$), 52

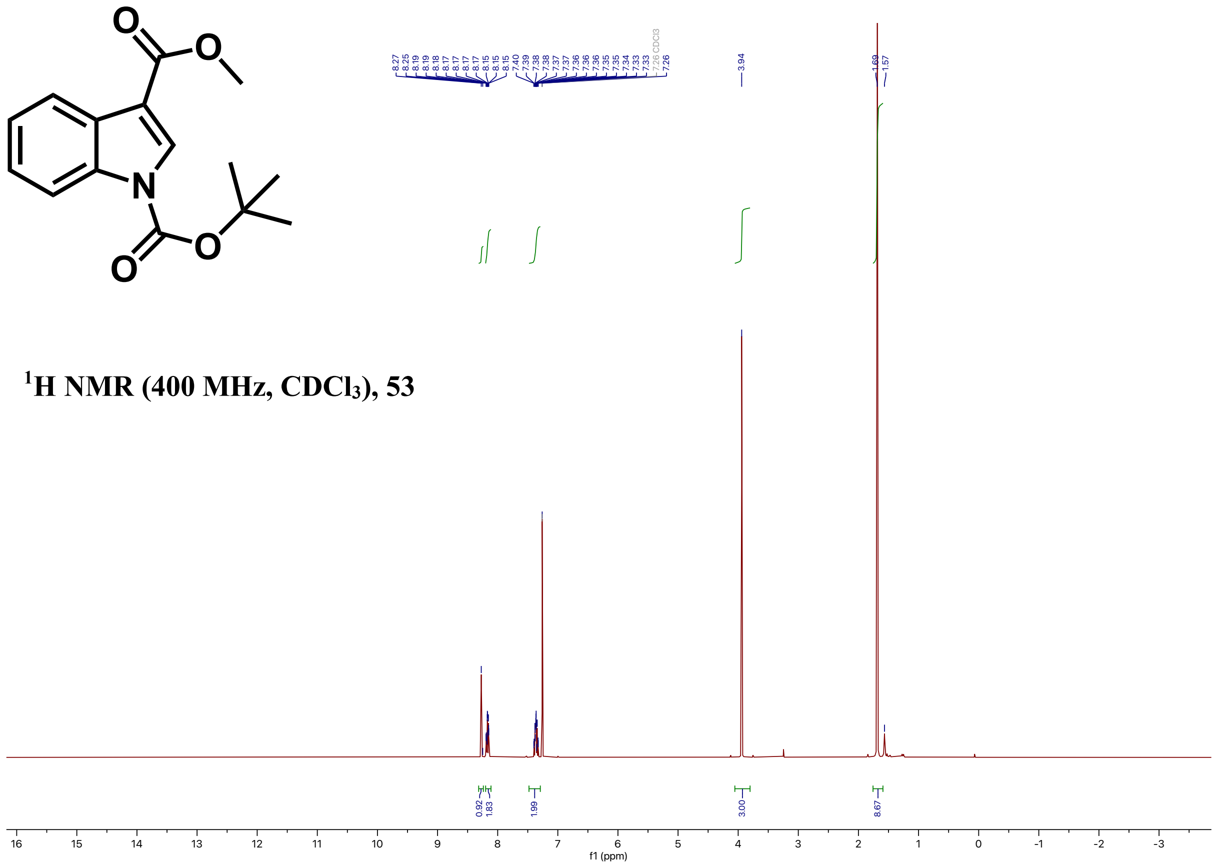

^{1}H NMR (400 MHz, $CDCl_3$), 53

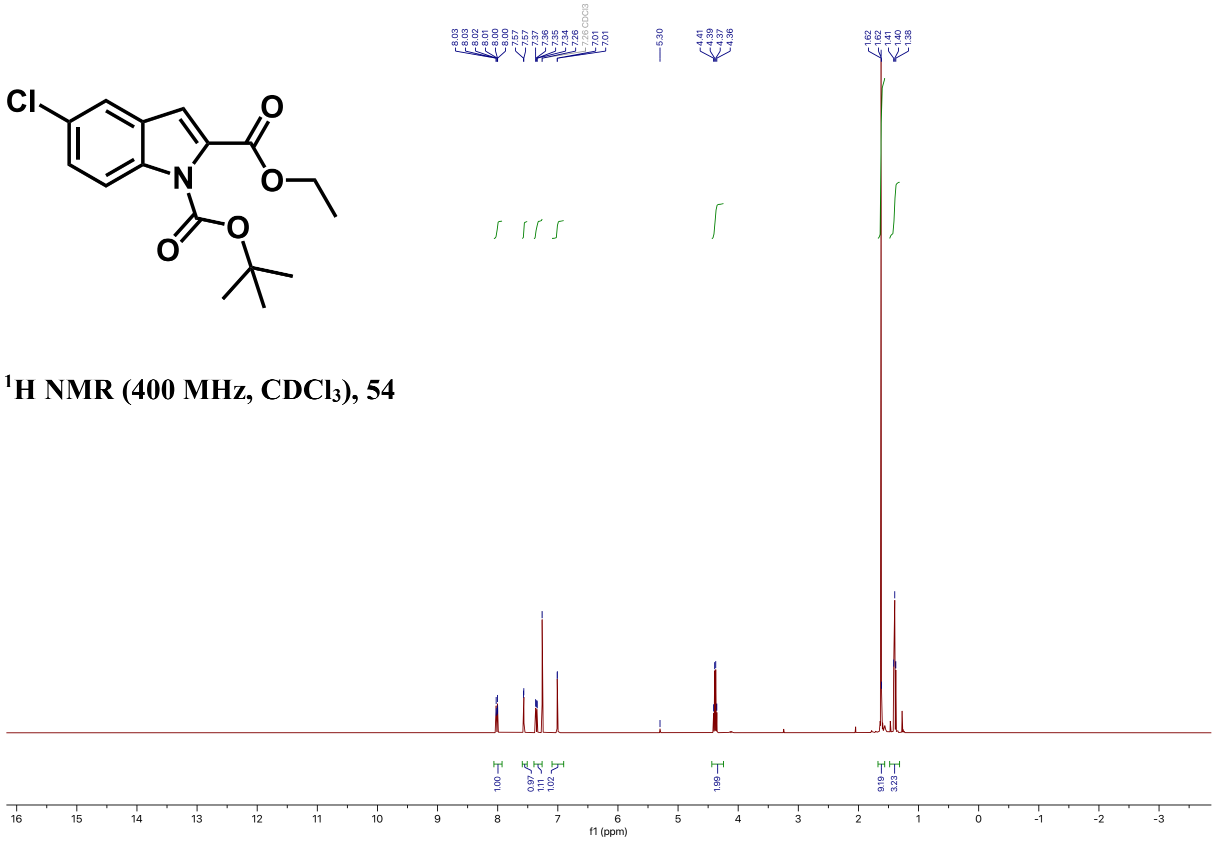

^{1}H NMR (400 MHz, $CDCl_3$), 54

10) Citation

(1) Han, J.; Haines, C. A.; Piane, J. J.; Filien, L. L.; Nacsa, E. D. An Electrochemical Design for Catalytic Dehydration: Direct, Room-Temperature Esterification without Acid or Base Additives. *J. Am. Chem. Soc.* **2023**, *145* (29), 15680–15687. https://doi.org/10.1021/jacs.3c04732.

(2) Guo, L.; Liu, Y.; Yao, W.; Leng, X.; Huang, Z. Iridium-Catalyzed Selective α-Alkylation of Unactivated Amides with Primary Alcohols. *Org. Lett.* **2013**, *15* (5), 1144–1147. https://doi.org/10.1021/ol400360g.

(3) Palao, E.; López, E.; Torres-Moya, I.; De La Hoz, A.; Díaz-Ortiz, Á.; Alcázar, J. Formation of Quaternary Carbons through Cobalt-Catalyzed C(Sp ^{3})–C(Sp ^{3}) Negishi Cross-Coupling. *Chem. Commun.* **2020**, *56* (59), 8210–8213. https://doi.org/10.1039/D0CC02734K.

(4) Vielhaber, T.; Topf, C. Manganese-Catalyzed Homogeneous Hydrogenation of Ketones and Conjugate Reduction of α,β-Unsaturated Carboxylic Acid Derivatives: A Chemoselective, Robust, and Phosphine-Free in Situ-Protocol. *Applied Catalysis A: General* **2021**, *623*, 118280. https://doi.org/10.1016/j.apcata.2021.118280.

(5) Wang, Y.; Aleiwi, B. A.; Wang, Q.; Kurosu, M. Selective Esterifications of Primary Alcohols in a Water-Containing Solvent. *Org. Lett.* **2012**, *14* (18), 4910–4913. https://doi.org/10.1021/ol3022337.

(6) Li, G.; Zatolochnaya, O. V.; Wang, X.-J.; Rodríguez, S.; Qu, B.; Desrosiers, J.-N.; Mangunuru, H. P. R.; Biswas, S.; Rivalti, D.; Karyakarte, S. D.; Sieber, J. D.; Grinberg, N.; Wu, L.; Lee, H.; Haddad, N.; Fandrick, D. R.; Yee, N. K.; Song, J. J.; Senanayake, C. H. BABIPhos Family of Biaryl Dihydrobenzooxaphosphole Ligands for Asymmetric Hydrogenation. *Org. Lett.* **2018**, *20* (7), 1725–1729. https://doi.org/10.1021/acs.orglett.8b00139.

(7) Ishikawa, F. Cyclic Guanidines. X. Synthesis of 2-(2,2-Disubstituted Ethenyl- and Ethyl)-2-Imidazolines as Potent Hypoglycemics. *Chem. Pharm. Bull.* **1980**, *28* (5), 1394–1402. https://doi.org/10.1248/cpb.28.1394.

(8) Matsuda, T.; Shiose, S.; Suda, Y. Rhodium-Catalyzed Double 1,4-Addition of Arylboronic Acids to β-Aryloxyacrylates Involving β-Oxygen Elimination. *Adv Synth Catal* **2011**, *353* (11–12), 1923–1926. https://doi.org/10.1002/adsc.201100253.

(9) Itagaki, N.; Kimura, M.; Sugahara, T.; Iwabuchi, Y. Organocatalytic Entry to Chiral Bicyclo[3. *n* .1]Alkanones via Direct Asymmetric Intramolecular Aldolization. *Org. Lett.* **2005**, *7* (19), 4185–4188. https://doi.org/10.1021/ol051569d.

(10) Whitmarsh-Everiss, T.; Olsen, A. H.; Laraia, L. Identification of Inhibitors of Cholesterol Transport Proteins Through the Synthesis of a Diverse, Sterol-Inspired Compound Collection. *Angew Chem Int Ed* **2021**, *60* (51), 26755–26761. https://doi.org/10.1002/anie.202111639.

(11) Clews, J.; Morgan, N.; Ramsden, C. Preparation of the I_3 Imidazoline Receptor Antagonist KU14R and Related 2,3-Dihydrobenzo[***b***]Furan Derivatives. *Synthesis* **2004**, *2001* (10), s-2001-16079. https://doi.org/10.1055/s-2001-16079.

(12) Hartwig, J.; Key, H.; Dydio, P.; Clack, D. ARTIFICIAL METALLOENZYMES CONTAINING NOBLE METAL - PORPHYRINS. US 2018 / 0305368 A1.

(13) Tan, E. Y. K.; Mat Lani, A. S.; Sow, W.; Liu, Y.; Li, H.; Chiba, S. Dearomatization of (Hetero)Arenes through Photodriven Interplay between Polysulfide Anions and Formate**. *Angew Chem Int Ed* **2023**, *62* (40), e202309764. https://doi.org/10.1002/anie.202309764.

(14) Pietruszka, J.; Simon, R. C. Indoline-3-Carboxylic Acid Derived Organocatalysts for the *Anti* - Mannich Reaction. *Chemistry A European J* **2010**, *16* (48), 14534–14544. https://doi.org/10.1002/chem.201002130.

(15) Doura, T.; Nonaka, H.; Sando, S. Atom Arrangement Strategy for Designing a Turn-on ^{1} H Magnetic Resonance Probe: A Dual Activatable Probe for Multimodal Detection of Hypochlorite. *Chem. Commun.* **2012**, *48* (10), 1565–1567. https://doi.org/10.1039/C1CC12044A.

(16) Kaiser, S.; Smidt, S. P.; Pfaltz, A. Iridium Catalysts with Bicyclic Pyridine–Phosphinite Ligands: Asymmetric Hydrogenation of Olefins and Furan Derivatives. *Angew Chem Int Ed* **2006**, *45* (31), 5194–5197. https://doi.org/10.1002/anie.200601529.
(17) Smith, G. C.; Zhang, D. H.; Zhang, W.; Soliven, A. H.; Wuest, W. M. Visible-Light/Nickel-Catalyzed Carboxylation of C(Sp ^{2}) Bromides via Formate Activation. *J. Org. Chem.* **2023**, *88* (13), 9565–9568. https://doi.org/10.1021/acs.joc.3c00895.
(18) Sun, X.; Duan, X.; Zheng, N.; Song, W. Gold-Catalyzed *Anti* -Markovnikov Oxidation of Au-Allenylidene to Generate Alkylidene Ketene. *Org. Lett.* **2023**, *25* (16), 2798–2805. https://doi.org/10.1021/acs.orglett.3c00682.
(19) Picard, B.; Fukuyama, T.; Bando, T.; Hyodo, M.; Ryu, I. Electron-Catalyzed Aminocarbonylation: Synthesis of α,β-Unsaturated Amides from Alkenyl Iodides, CO, and Amines. *Org. Lett.* **2021**, *23* (24), 9505–9509. https://doi.org/10.1021/acs.orglett.1c03714.
(20) Knaus, T.; Mutti, F. G.; Humphreys, L. D.; Turner, N. J.; Scrutton, N. S. Systematic Methodology for the Development of Biocatalytic Hydrogen-Borrowing Cascades: Application to the Synthesis of Chiral α-Substituted Carboxylic Acids from α-Substituted α,β-Unsaturated Aldehydes. *Org. Biomol. Chem.* **2015**, *13* (1), 223–233. https://doi.org/10.1039/C4OB02282C.
(21) Cao, H.-Q.; Liu, H.-N.; Liu, Z.-Y.; Qiao, B.; Zhang, F.-G.; Ma, J.-A. Silver-Promoted Direct Phosphorylation of Bulky C(Sp ^{2})–H Bond to Build Fully Substituted β-Phosphonodehydroamino Acids. *Org. Lett.* **2020**, *22* (16), 6414–6419. https://doi.org/10.1021/acs.orglett.0c02229.
(22) Lee, D.; Yang, Y.; Yun, J. Copper-Catalyzed Asymmetric Reduction of 3,3-Diarylacrylonitriles. *Org. Lett.* **2007**, *9* (14), 2749–2751. https://doi.org/10.1021/ol0712071.
(23) Wang, D.; Wang, Y.; Zhao, J.; Shen, M.; Hu, J.; Liu, Z.; Li, L.; Xue, F.; Yu, P. Strategic Approach to 8-Azacoumarins. *Org. Lett.* **2017**, *19* (5), 984–987. https://doi.org/10.1021/acs.orglett.6b03771.
(24) Zhang, Q.; Zhang, L.; Tang, C.; Luo, H.; Cai, X.; Chai, Y. Cascade Reaction of Propargylic Alcohols with Hydroxylamine Hydrochloride: Facile Synthesis of α,β-Unsaturated Oximes and Nitriles. *Tetrahedron* **2016**, *72* (44), 6935–6942. https://doi.org/10.1016/j.tet.2016.09.010.
(25) Zhang, M.; Shvetsova, O.; De Bo, G. Expedient Synthesis of Heterobifunctional Triarylmethane Stoppers for Macromolecular Rotaxanes. *J. Org. Chem.* **2020**, *85* (4), 2770–2774. https://doi.org/10.1021/acs.joc.9b03063.
(26) Sim, J.; Viji, M.; Rhee, J.; Jo, H.; Cho, S. J.; Park, Y.; Seo, S.; Jung, K.; Lee, H.; Jung, J. *γ* -Functionalization of *α,β* -Unsaturated Nitriles under Mild Conditions: Versatile Synthesis of 4-Aryl-2-Bromopyridines. *Adv Synth Catal* **2019**, *361* (23), 5458–5465. https://doi.org/10.1002/adsc.201901002.
(27) Rabal, O.; Sánchez-Arias, J. A.; Cuadrado-Tejedor, M.; De Miguel, I.; Pérez-González, M.; García-Barroso, C.; Ugarte, A.; Estella-Hermoso De Mendoza, A.; Sáez, E.; Espelosin, M.; Ursua, S.; Haizhong, T.; Wei, W.; Musheng, X.; Garcia-Osta, A.; Oyarzabal, J. Design, Synthesis, and Biological Evaluation of First-in-Class Dual Acting Histone Deacetylases (HDACs) and Phosphodiesterase 5 (PDE5) Inhibitors for the Treatment of Alzheimer's Disease. *J. Med. Chem.* **2016**, *59* (19), 8967–9004. https://doi.org/10.1021/acs.jmedchem.6b00908.
(28) Song, L.; Claessen, S.; Van Der Eycken, E. V. Pyridine-Enabled C–N Bond Activation for the Rapid Construction of Amides and 4-Pyridylglyoxamides by Cooperative Palladium/Copper Catalysis. *J. Org. Chem.* **2020**, *85* (12), 8045–8054. https://doi.org/10.1021/acs.joc.0c00845.
(29) Makida, Y.; Saita, M.; Kuramoto, T.; Ishizuka, K.; Kuwano, R. Asymmetric Hydrogenation of Azaindoles: Chemo- and Enantioselective Reduction of Fused Aromatic Ring Systems Consisting of Two Heteroarenes. *Angew Chem Int Ed* **2016**, *55* (39), 11859–11862. https://doi.org/10.1002/anie.201606083.
(30) O'Brien, A. G.; Lévesque, F.; Seeberger, P. H. Continuous Flow Thermolysis of Azidoacrylates for the Synthesis of Heterocycles and Pharmaceutical Intermediates. *Chem. Commun.* **2011**, *47* (9), 2688–2690. https://doi.org/10.1039/C0CC04481D.

(31) Kong, W.-J.; Liu, Y.-J.; Xu, H.; Chen, Y.-Q.; Dai, H.-X.; Yu, J.-Q. Pd-Catalyzed α-Selective C–H Functionalization of Olefins: En Route to 4-Imino-β-Lactams. *J. Am. Chem. Soc.* **2016**, *138* (7), 2146–2149. https://doi.org/10.1021/jacs.5b13353.
(32) Jagadeesh, R. V.; Murugesan, K.; Alshammari, A. S.; Neumann, H.; Pohl, M.-M.; Radnik, J.; Beller, M. MOF-Derived Cobalt Nanoparticles Catalyze a General Synthesis of Amines. *Science* **2017**, *358* (6361), 326–332. https://doi.org/10.1126/science.aan6245.
(33) Lee, W.-I.; Jung, J.-W.; Sim, J.; An, H.; Suh, Y.-G. Microwave-Assisted Synthesis of 3-Substituted Indoles via Intramolecular Arene–Alkene Coupling of o-Iodoanilino Enamines. *Tetrahedron* **2013**, *69* (35), 7211–7219. https://doi.org/10.1016/j.tet.2013.06.101.
(34) Gazvoda, M.; Krivec, M.; Časar, Z.; Košmrlj, J. En Route to 2-(Cyclobuten-1-Yl)-3-(Trifluoromethyl)-1 *H* -Indole. *J. Org. Chem.* **2018**, *83* (4), 2486–2493. https://doi.org/10.1021/acs.joc.8b00100.
